# Induced proximity to PML protects TDP-43 from aggregation via SUMO–ubiquitin networks

**DOI:** 10.1038/s41589-025-01886-4

**Published:** 2025-04-17

**Authors:** Kristina Wagner, Jan Keiten-Schmitz, Bikash Adhikari, Upayan Patra, Koraljka Husnjak, François McNicoll, Dorothee Dormann, Michaela Müller-McNicoll, Georg Tascher, Elmar Wolf, Stefan Müller

**Affiliations:** 1 Institute of Biochemistry II, Goethe University Frankfurt, Faculty of Medicine, Frankfurt am Main, Germany; 2 Biochemisches Institut, Christian-Albrechts-Universität, Kiel, Germany; 3 Institute of Molecular Biosciences, Goethe University Frankfurt, Frankfurt am Main, Germany; 4 Biocenter, Institute of Molecular Physiology, Johannes Gutenberg-Universität (JGU), Mainz, Germany; 5 Institute of Molecular Biology (IMB), Mainz, Germany; 6 Present Address: BioNTech AG, Mainz, Germany

**Keywords:** Cell biology, Biochemistry

## Abstract

The established role of cytosolic and nuclear inclusions of TDP-43 in the pathogenesis of neurodegenerative disorders has multiplied efforts to understand mechanisms that control TDP-43 aggregation and has spurred searches for approaches limiting this process. Formation and clearance of TDP-43 aggregates are controlled by an intricate interplay of cellular proteostasis systems that involve post-translational modifications and frequently rely on spatial control. We demonstrate that attachment of the ubiquitin-like SUMO2 modifier compartmentalizes TDP-43 in promyelocytic leukemia protein (PML) nuclear bodies and limits the aggregation of TDP-43 in response to proteotoxic stress. Exploiting this pathway through proximity-inducing recruitment of TDP-43 to PML triggers a SUMOylation–ubiquitylation cascade protecting TDP-43 from stress-induced insolubility. The protective function of PML is mediated by ubiquitylation in conjunction with the p97 disaggregase. Altogether, we demonstrate that SUMO–ubiquitin networks protect cells from insoluble TDP-43 inclusions and propose the functionalization of PML as a potential future therapeutic avenue countering aggregation.

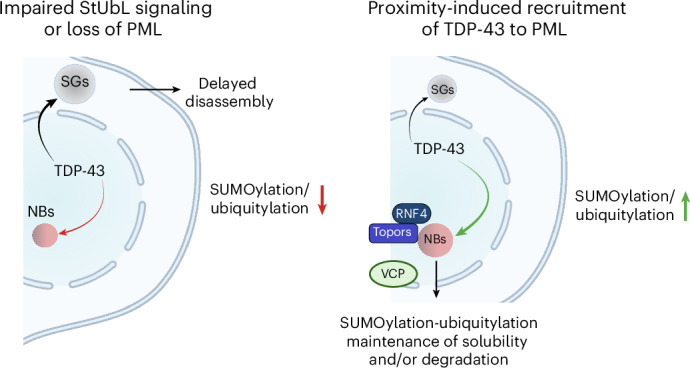

## Abstract (web summary)

TDP-43 aggregates are a hallmark of neurodegeneration. Wagner et al. show that SUMOylation/ubiquitylation of TDP-43, mediated by its recruitment to the promyelocytic leukemia protein nuclear bodies, prevents its aggregation under proteotoxic stresses.

## Main

Protein inclusions are a hallmark of neurodegenerative disease, and TAR DNA-binding protein 43 (TDP-43) inclusions are associated with the pathogenesis of amyotrophic lateral sclerosis (ALS), frontotemporal dementia (FTD) and Alzheimer’s disease (AD)^1^. TDP-43 is an RNA-binding protein involved in RNA transactions, such as RNA transport, polyadenylation and splicing^2^. TDP-43 shuttles between the nuclear and cytosolic compartments and comprises an N-terminal oligomerization domain, two tandem RNA recognition motifs (RRMs) and a C-terminal low-complexity domain exhibiting prion-like properties^3^. Under physiological conditions, TDP-43 is primarily found in the nucleus, where it binds to pre-mRNA and is associated with subnuclear membrane-less compartments, for example, paraspeckles and Cajal bodies. In response to cellular stress, a fraction of TDP-43 is released from the nucleus and transiently accumulates in cytosolic stress granules (SGs) and other cytosolic droplets^3^. Following the resolution of stress and SG disassembly, TDP-43 relocates to the nucleus. However, it has been proposed that when SGs persist upon removal of stress, they can be converted to solid inclusions as found in ALS or FTD^4,5^. Proteotoxic stress not only affects the nuclear-cytosolic distribution of TDP-43 but also triggers the compartmentalization of TDP-43 in nuclear condensates, such as promyelocytic leukemia protein (PML) nuclear bodies (NBs), paraspeckles or anisosomes^6–8^. While TDP-43 is initially sheltered from aggregation in these liquid-like structures, persistent stress or a failure in cellular proteostasis systems can convert TDP-43 condensates into amyloid-type insoluble structures^9,10^. Insoluble cytosolic and nuclear TDP-43 inclusions are found in the degenerating brain regions of most patients suffering from ALS or FTD^11,12^, suggesting that the inclusions contribute to cytotoxicity in these pathologies. Furthermore, nuclear and cytosolic TDP-43 aggregates are found in a large fraction of patients with AD and a more recently recognized age-associated dementia termed limbic-predominant age-related TDP-43 encephalopathy^13^.

Post-translational modifications have emerged as critical switches governing TDP-43 transitions from a liquid-like phase to a solid state. TDP-43 undergoes phosphorylation in its C-terminal domain, and this modification has generally been associated with an enhanced aggregation propensity. In line with this idea, hyperphosphorylated TDP-43 was identified as a typical constituent of the insoluble cytoplasmic inclusions in the patient brain samples^12^. However, recent work has challenged this view by providing compelling evidence that the C-terminal hyperphosphorylation limits the aggregation of TDP-43 by keeping TDP-43 condensates in a more liquid state^14^. Acetylation of lysine residues within the RRM domains of TDP-43 compromises its RNA binding and has been linked to nuclear phase separation^9,15^. TDP-43 also undergoes exquisite modification by ubiquitin, and pathogenic inclusions are generally characterized by the presence of ubiquitylated TDP-43 (refs. ^11,12,16^). The canonical ubiquitin-proteasome system (UPS) typically targets proteins for degradation by the formation of ubiquitin chains, but depending on the chain topology ubiquitylation can also mediate nonproteolytic functions^17^. The chain topology, pathophysiological significance and functional consequences of TDP-43 ubiquitylation are not well understood.

We and others have shown that TDP-43 also undergoes modification by the small ubiquitin-like modifier (SUMO)^18–20^. SUMO (SUMO1 or the highly related SUMO2/SUMO3 proteins) is covalently attached to lysine residues of target proteins in a process involving an E1 activating enzyme, an E2 conjugating enzyme and E3 SUMO ligases^21^. SUMOylation generally controls the dynamics of nuclear protein assemblies by either preventing or promoting distinct protein–protein interactions^22^. A paradigmatic example of SUMO-dependent formation of a nuclear multiprotein assembly is PML NBs^23,24^. Like ubiquitin, SUMO, in particular the SUMO2/SUMO3 paralogs, can form polymeric chains via internal lysine residues, and chain formation is typically triggered upon exposure of cells to proteotoxic or genotoxic insults^25^. SUMO chains can prime proteins for ubiquitylation by distinct SUMO-targeted ubiquitin ligases (StUbLs)^25^. The prototypical StUbL RING finger protein 4 (RNF4) binds to SUMO2 chains and ubiquitylates proteins that are marked by polySUMO chains^26,27^. RNF4 can trigger the formation of canonical proteasome-targeting K48-linked Ub chains, nonproteolytic K63-linked chains as well as hybrid SUMO-Ub chains that are not well characterized yet^25,28^. Very recent work revealed that the E3 ubiquitin ligase Topors functions as a StUbL in parallel to RNF4 (refs. ^29,30^). Our published work demonstrated that stress-induced SUMOylation of TDP-43 primes it for ubiquitylation by RNF4 (ref. ^18^). The direct functional consequences of SUMO-primed ubiquitylation on TDP-43 were not fully uncovered, but we found that the nuclear StUbL pathway is generally involved in the disassembly of cytosolic SGs, supporting the idea that it counters the build-up of cellular aggregates.

Following this idea, we aimed to functionalize the SUMO pathway to potentially limit TDP-43 aggregation. In proof of concept experiments, we validated that the fusion of SUMO2 to TDP-43 enhances its solubility in response to proteotoxic stress, correlating with its compartmentalization in PML nuclear condensates. By using a system for the proximity-inducing recruitment of TDP-43 to PML, we demonstrated that PML triggers a SUMOylation–ubiquitylation cascade on WT TDP-43 and ALS-associated TDP-43 variants. We further demonstrated that PML-induced ubiquitylation protects TDP-43 from stress-induced aggregation in a pathway that requires the p97 disaggregase. Altogether, these data pave the way to develop innovative proximity-inducing pharmacology limiting TDP-43 aggregation^31^.

## Results

### Attachment of SUMO2 protects TDP-43 from stress-induced aggregation

**Fig. 1:**
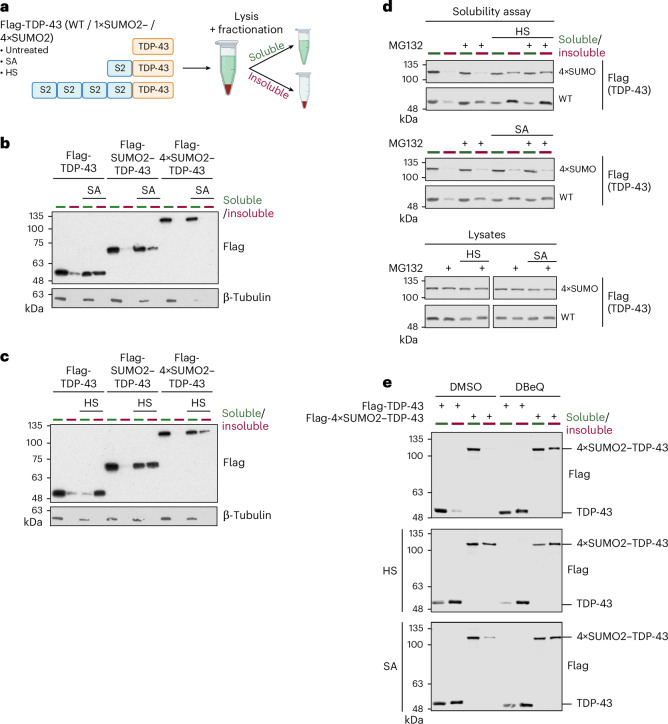
Attachment of SUMO2 protects TDP-43 from stress-induced aggregation. **a**, Schematic representation of the solubility assay. HEK293T cells transiently expressing Flag-TDP-43, Flag-SUMO2–TDP-43 or Flag-4×SUMO2–TDP-43 were lysed in an NP-40-containing lysis buffer. Before lysis, cells were exposed to HS (43 °C, 1 h), SA (0.5 mM, 1 h) or left untreated. Lysates were fractionated into an NP-40-insoluble pellet and an NP-40-soluble supernatant fraction and analyzed by western blotting. Panel **a** is created with BioRender.com. **b**, Solubility assay. HEK293T cells expressing Flag-TDP-43, Flag-SUMO2–TDP-43 or Flag-4×SUMO2–TDP-43 for 48 h were treated with SA (0.5 mM, 1 h) or left untreated before lysis and fractionation (as described in **a**). NP-40-soluble/NP-40-insoluble fractions are indicated by green and red bars. Statistical analysis of three independent replicates is provided in Extended Data Fig. 1a. **c**, As in **b**, but cells were subjected to HS (43 °C, 1 h) or left untreated. Statistical analysis of three independent replicates is provided in Extended Data Fig. 1a. **d**, As in **b** and **c**, but cells were additionally treated with MG132 (10 µm, 4 h) or DMSO. Statistical analysis of three independent replicates is provided in Extended Data Fig. 1c. **e**, As in **b**–**d**, but cells were treated with DBeQ (20 µm, 4 h) or DMSO. Statistical analysis of three independent replicates is provided in Extended Data Fig. 1d. HS, heat stress; SA, sodium arsenite. Source data

**Extended Data Fig. 1:**
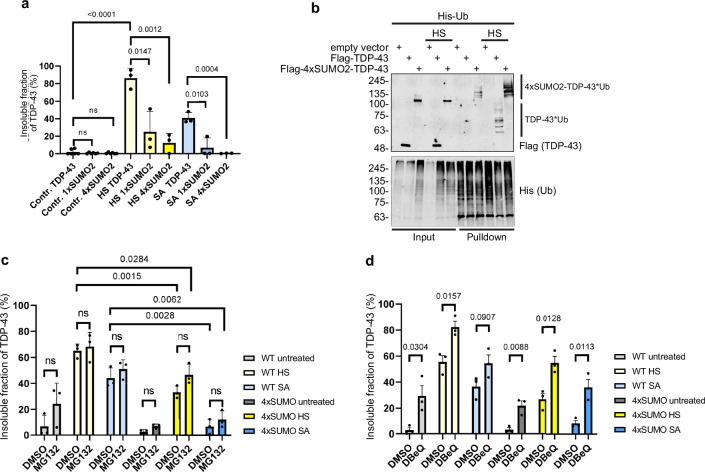
Attachment of SUMO2 protects TDP-43 from stress-induced aggregation. **a**, Statistical analysis of TDP-43–FKBP insolubility in response to heat stress or treatment with sodium arsenite. The insoluble fraction (in % of total Flag-TDP-43) of Flag-TDP-43–FKBP (WT, 1×SUMO2 or 4×SUMO2) in solubility assays as shown in Fig. 1b,c. P values of two-tailed, unpaired Student’s t-tests are indicated; error bars show the standard deviation of the mean (n = 3). **b**, Enrichment of ubiquitylated TDP-43 by Ni-NTA pull down. HEK293T cells were transfected with His-Ub together with Flag-TDP-43, Flag-4×SUMO2–TDP-43 or an empty vector for 48 h and were treated with heat stress (43 °C, 1 h) or left untreated prior to lysis. Ubiquitylated proteins were enriched by denaturing Ni-NTA pull down. **c**, Statistical analysis of Flag-TDP-43 (WT, 1×SUMO2 or 4×SUMO2) insolubility in response to heat stress or treatment with sodium arsenite and MG132 (10 µm, 4 h) as shown in Fig. 1d. P values of two-tailed unpaired Student’s t-tests are indicated; error bars show the standard deviation of the mean (n = 3). **d**, Statistical analysis of Flag-TDP-43 (WT, 1×SUMO2 or 4×SUMO2) insolubility in response to heat stress or treatment with sodium arsenite and DBeQ (20 µm, 4 h) as shown in Fig. 1e. P values of two-tailed unpaired Student’s t-tests are indicated; error bars show the standard deviation of the mean (n = 3). Source data

Previous work by our laboratory and others has demonstrated SUMO modification of TDP-43 in response to proteotoxic stress^18,32^. Mass spectrometry (MS) data show that TDP-43 undergoes extensive SUMOylation at up to 15 different lysine residues, with at least ten sites showing enhanced modification by SUMO2 in response to proteotoxic stress^33^. The modification of multiple SUMO sites within TDP-43 complicates functional studies that rely on SUMO-deficient mutants. We, therefore, chose an alternative approach by mimicking SUMOylation via fusing SUMO2 either as a monomer (SUMO2–TDP-43) or as a linear chain of four tandemly repeated SUMO2 moieties (tetra-SUMO2–TDP-43/4×SUMO2–TDP-43) to the N terminus of TDP-43 (Fig. 1a). Next, we explored how the attachment of SUMO alters the aggregation propensity of TDP-43 in response to proteotoxic stress. To this end, we transiently expressed Flag-tagged wild-type (WT) TDP-43, Flag-SUMO2–TDP-43 and Flag-tetra-SUMO2–TDP-43 in HEK293T cells and exposed cells 1 h before lysis to heat stress at 43 °C or treated them with sodium arsenite, an inducer of oxidative stress. Upon lysis, cellular proteins were separated into a detergent-soluble and detergent-insoluble fraction (Fig. 1a), and TDP-43 variants in both fractions were analyzed by anti-Flag immunoblotting (Fig. 1b,c and Extended Data Fig. 1a). In control cells, WT TDP-43 as well as the SUMO2 fusion proteins are predominantly found in the soluble fraction. However, when cells were exposed to sodium arsenite, around 40% of WT TDP-43 was shifted to the insoluble fraction. This shift to the insoluble fraction is limited to less than 10% in case of the mono-SUMO2–TDP-43 protein and almost completely abrogated in case of the tetra-SUMO2–TDP-43 variant (Fig. 1b and Extended Data Fig. 1a). Upon exposure of cells to heat stress, 80% of WT TDP-43 is found in the insoluble fraction, while mono-SUMO2–TDP-43 remains partly soluble and tetra-SUMO2–TDP-43 stays predominantly in the soluble fraction (Fig. 1c and Extended Data Fig. 1a). This data indicate that the attachment of SUMO2, and in particular of SUMO2 chains, to TDP-43 limits its aggregation propensity under stress. In line with previous work, defining SUMOylation as a primer for TDP-43 ubiquitylation, the tetra-SUMO2–TDP-43 variant undergoes ubiquitylation under basal conditions and exhibits more pronounced stress-induced ubiquitylation when compared to the WT protein (Extended Data Fig. 1b). Notably, however, the improved solubility observed upon SUMO-primed ubiquitylation of TDP-43 under heat or arsenite stress persists in the presence of the proteasome inhibitor MG132, indicating that it is not primarily mediated by canonical degradative UPS signaling (Fig. 1d and Extended Data Fig. 1c). Ubiquitylation can mediate the extraction of proteins from larger protein assemblies in a pathway that often involves the ubiquitin-selective segregase p97 (alias valosin-containing protein, VCP)^34^. Intriguingly, inhibition of p97 activity by the small molecule inhibitor N2,N4-dibenzylquinazoline-2,4-diamine (DBeQ) abrogates the solubilizing function of the SUMO2 chain fusion in response to heat or arsenite stress, indicating involvement of p97 in this process (Fig. 1e and Extended Data Fig. 1d). Altogether, these observations suggest that SUMO2, possibly in conjunction with ubiquitylation, promotes solubility of TDP-43 in response to heat or oxidative stress.

### SUMO2-modified TDP-43 is associated with PML NBs

**Fig. 2:**
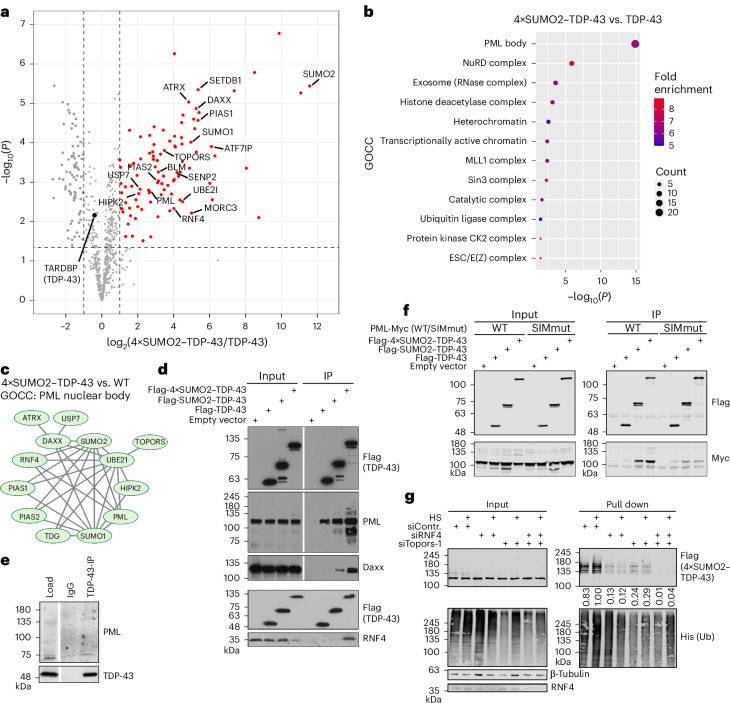
SUMO2-modified TDP-43 is associated with PML NBs. **a**, Volcano plot displaying the results of IP-MS in HEK293T cells expressing Flag-4×SUMO2–TDP-43 or Flag-TDP-43. The difference in the log_2_ mean LFQ intensities between IPs from Flag-4×SUMO2–TDP-43 and Flag-TDP-43 expressing cells was plotted against the negative logarithmized *P* values of a two-sided Student’s *t* test (*n* = 3). Proteins significantly enriched in IPs from cells expressing Flag-4×SUMO2–TDP-43 (log_2_(4×SUMO2–TDP-43/TDP-43) > 1 and *P* < 0.05) are highlighted in red. TDP-43 and proteins associated with PML NBs are indicated. **b**, GOCC enrichment analysis of proteins interacting with Flag-4×SUMO2–TDP-43. Proteins that were exclusively identified in all three replicates of IPs from Flag-4×SUMO2–TDP-43 expressing cells and proteins that were significantly enriched in IPs from Flag-4×SUMO2–TDP-43 expressing cells (versus Flag-TDP-43) were combined into a dataset and used for GOCC enrichment analysis. Greater than fivefold enriched GOCC terms are shown. **c**, STRING cluster of proteins that were significantly enriched in **a** and are associated with the GOCC term ‘PML body’. **d**, HEK293T cells were transfected with Flag-4×SUMO2–TDP-43, Flag-SUMO2–TDP-43, Flag-TDP-43 or an empty vector. Flag-IP was performed to enrich TDP-43 variants and interacting proteins. Coprecipitated PML, Daxx and RNF4 were detected. **e**, Endogenous TDP-43 was immunoprecipitated from HEK293T cell lysates, and coprecipitation of PML was detected. Two major isoforms of PML are detected. **f**, HEK293T cells were transfected with Myc-tagged PML (WT or SIMmut) in combination with Flag-TDP-43 (WT, 1×SUMO, 4×SUMO) or an empty vector. TDP-43 was enriched by Flag-IP, and co-immunoprecipitated PML variants were detected. **g**, Enrichment of ubiquitylated Flag-4×SUMO2–TDP-43 by Ni-NTA pull down. HEK293T cells were depleted of RNF4 and Topors by siRNA. Subsequently, cells were transfected with His-Ub and Flag-4×SUMO2–TDP-43 and exposed to HS (43 °C, 1 h) or left untreated. Ubiquitylated proteins were enriched by denaturing Ni-NTA pull down. Signal intensities of high-molecular-weight bands (running above the height of unmodified Flag-4×SUMO2–TDP-43) in pull-down samples (anti-Flag) were quantified and normalized to the matching input intensities. Source data

**Extended Data Fig. 2:**
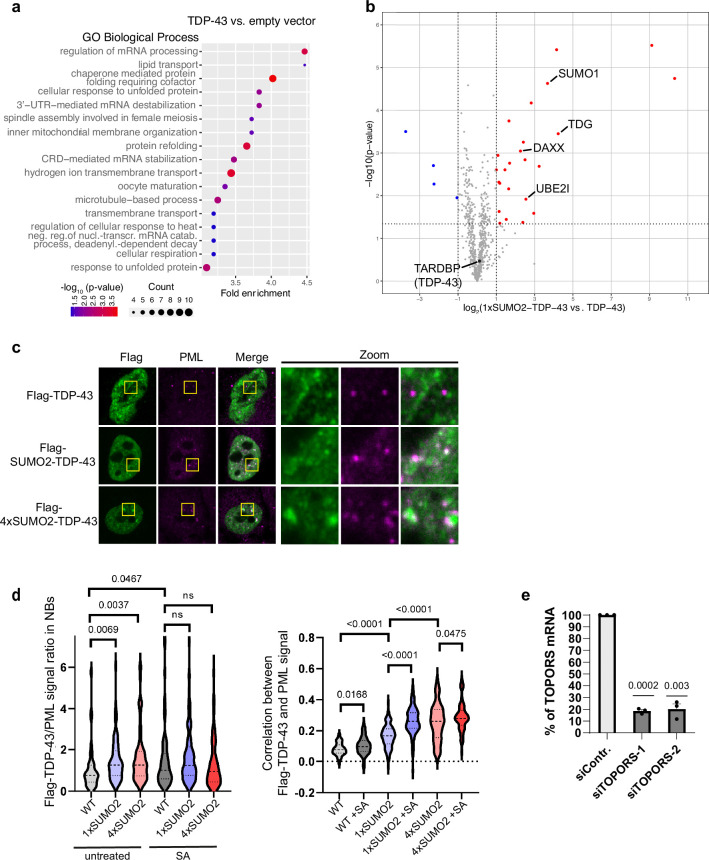
SUMO2-modified TDP-43 is associated with PML NBs. **a**, GO biological process (GOBP) enrichment analysis of proteins interacting with Flag-TDP-43. Proteins that were exclusively identified in all three replicates of IPs from Flag-TDP-43 expressing cells and proteins that were significantly enriched in IPs from Flag-TDP-43 expressing cells (vs. Flag-TDP-43) were combined into a dataset and used for GOBP enrichment analysis. Selected GOBP terms with at least threefold enrichment are shown. **b**, Volcano plot displaying the results of interactomes performed in HEK293T cells expressing Flag-SUMO2–TDP-43 or Flag-TDP-43. The difference of the log_2_ mean LFQ intensities between Flag-1×SUMO2–TDP-43 and Flag-TDP-43 expressing cells was plotted against the negative logarithmised p values of a Student’s t-test. Proteins significantly enriched in IPs from cells expressing Flag-SUMO2–TDP-43 (log_2_(SUMO2–TDP-43/TDP-43) > 1 and p value < 0.05) are highlighted in red. TDP-43 and proteins associated with PML NBs are indicated. **c**, Immunofluorescence images of HeLa cells transfected with either Flag-TDP-43, Flag SUMO2-TDP-43 or Flag-4xSUMO2-TDP-43. Endogenous PML is shown in magenta. **d**, Statistical analysis of Flag-TDP-43 (WT, 1xSUMO2 or 4xSUMO2) enrichment in PML NBs in HeLa cells as shown in **c**. Left: The Flag-TDP-43/PML signal ratio in the combined area of all PML NBs in a cell was determined and normalised to Flag-TDP-43 expression levels in the nucleus of the same cell. Right: The correlation of Flag-TDP-43 and PML signals within the nucleus was determined. P-values of two-tailed, unpaired student’s t-tests are shown. At least 51 cells were measured per condition. **e**, The efficiency of Topors knockdowns was determined by qPCR. HEK293T cells were transfected for 72 hours with a control siRNA or one of the two siRNAs against Topors. P-values of one sample t-tests are indicated (*n* = 3). Source data

To get insights into the underlying mechanism(s), we asked whether the attachment of SUMO2 alters the interactome of TDP-43. By Flag-immunoprecipitation (IP) followed by MS (IP-MS), we identified 183 TDP-43-associated proteins that were at least twofold enriched when comparing anti-Flag-TDP-43 immunoprecipitates with immunoprecipitates from mock-transfected control cells (Supplementary Data). Consistent with published data^35^, gene ontology (GO) term analysis of TDP-43-interacting proteins reveals a significant functional enrichment of proteins involved in splicing and other RNA-related processes (Extended Data Fig. 2a). Notably, in immunoprecipitates of SUMO2–TDP-43 and tetra-SUMO2–TDP-43 variants, an additional set of 23 and 95 proteins, respectively, was at least twofold enriched when compared to WT TDP-43 (Fig. 2a, Extended Data Fig. 2b and Supplementary Data). Cellular component analysis shows the preferential association of the tetra-SUMO2–TDP-43 variant with proteins that are physically and functionally connected to PML NBs (Fig. 2b). PML NBs are subnuclear domains that have emerged as compartments of nuclear protein quality control^18,32,36–38^. STRING network analysis of the tetra-SUMO2–TDP-43 enriched proteins shows a highly interconnected network comprising PML, the defining component of PML NBs, as well as NB-associated key markers, such as SetDB1 or Daxx^39^. Furthermore, tetra-SUMO2–TDP-43 interacts with NB-associated components of the SUMO machinery, including the E3 ligases of the protein inhibitor of activated STAT (PIAS) family as well as the StUbLs RNF4 and Topors (Fig. 2c). IP followed by immunoblotting validated the association of WT TDP-43 with endogenous PML upon transient expression and also revealed a weak interaction of both proteins at their endogenous expression levels (Fig. 2d,e). Consistent with the MS data, the SUMO2-conjugated TDP-43 variants exhibit stronger binding to endogenous PML, as well as Daxx and RNF4 (Fig. 2d). Fusion of SUMO2, and in particular the tetra-SUMO2 chains to TDP-43, strongly enhances binding to WT PML, but not to a PML mutant harboring a defective SUMO interaction motif (SIM), indicating that SUMOylated TDP-43 promotes binding to the SIM domain of PML (Fig. 2f). Immunofluorescence experiments confirm compartmentalization of SUMO2–TDP-43 in PML NBs under basal conditions and in response to arsenite treatment (Extended Data Fig. 2c,d). Based on the observed binding of both RNF4 and Topors to tetra-SUMO2–TDP-43, we explored whether these StUbLs catalyze the ubiquitylation of tetra-SUMO2–TDP-43. Compared to control conditions, siRNA-mediated depletion of either RNF4 or Topors reduces ubiquitylation of tetra-SUMO2–TDP-43, while codepletion of both proteins abolishes the modification (Fig. 2g and Extended Data Fig. 2e).

Altogether, our data indicate that the linkage of SUMO2 chains to TDP-43 limits its aggregation propensity under stress, correlating with enhanced ubiquitylation and sequestration in PML NBs.

### Recruitment to PML enhances SUMOylation of TDP-43

**Fig. 3:**
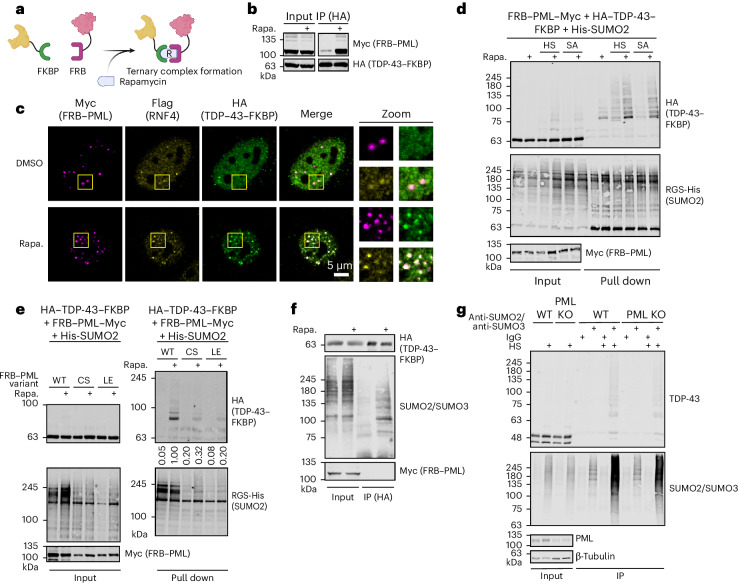
Recruitment to PML enhances SUMOylation of TDP-43. **a**, Model of the rapamycin-induced recruitment of PML to TDP-43. FKBP was fused to the C terminus of HA–TDP-43, and FRB was fused to the N terminus of PML–Myc. Upon addition of rapamycin, HA–TDP-43–FKBP is recruited into a ternary complex with FRB–PML–Myc and rapamycin. Panel **a** is created with BioRender.com. **b**, HA-IP was performed in HEK293T cells expressing FRB–PML–Myc and HA–TDP-43–FKBP for 48 h. Cells were mock-treated or treated with rapamycin (100 nM, 4 h). **c**, HeLa cells expressing endogenously tagged RNF4-3×Flag were transfected with FRB–PML–Myc and HA–TDP-43–FKBP for 24 h and either mock-treated or treated with rapamycin (100 nM, 3 h). Inset areas are indicated with yellow squares. Scale bar = 5 µm. Statistical analysis is provided in Extended Data Fig. 3a. **d**, Enrichment of SUMOylated HA–TDP-43–FKBP by Ni-NTA pull down. HEK293T cells were transfected with His-SUMO2 together with FRB–PML–Myc and HA–TDP-43–FKBP for 48 h and were treated with HS (43 °C, 1 h), SA (0.5 mM, 1 h) or left untreated. Rapamycin was added where indicated (100 nM, 4 h). SUMOylated proteins were enriched by denaturing Ni-NTA pull down. **e**, As in **d**, but cells were left untreated. HEK293T cells were transfected with FRB–PML–Myc (WT, C57,60S or L73E) together with HA–TDP-43–FKBP and His-SUMO2. Signal intensities of high-molecular-weight bands (running above the height of unmodified HA–TDP-43–FKBP) in pull-down samples (anti-HA) were quantified and normalized to the respective intensities in the matching input lane. Relative values are listed below the top right panel. **f**, HeLa cells expressing TDP-43–FKBP–HA from the endogenous TDP-43 locus together with FRB–PML–Myc were treated with DMSO or 100 nM rapamycin for 4 h. TDP-43–FKBP–HA was enriched by HA-IP under denaturing conditions and immunoblotted for SUMO2/SUMO3 conjugates. **g**, U2OS WT and PML-KO cells were subjected to 1 h of HS at 43 °C or left untreated. SUMOylated proteins were enriched by IP. Rapa., rapamycin; KO, knockout. Source data

**Extended Data Fig. 3:**
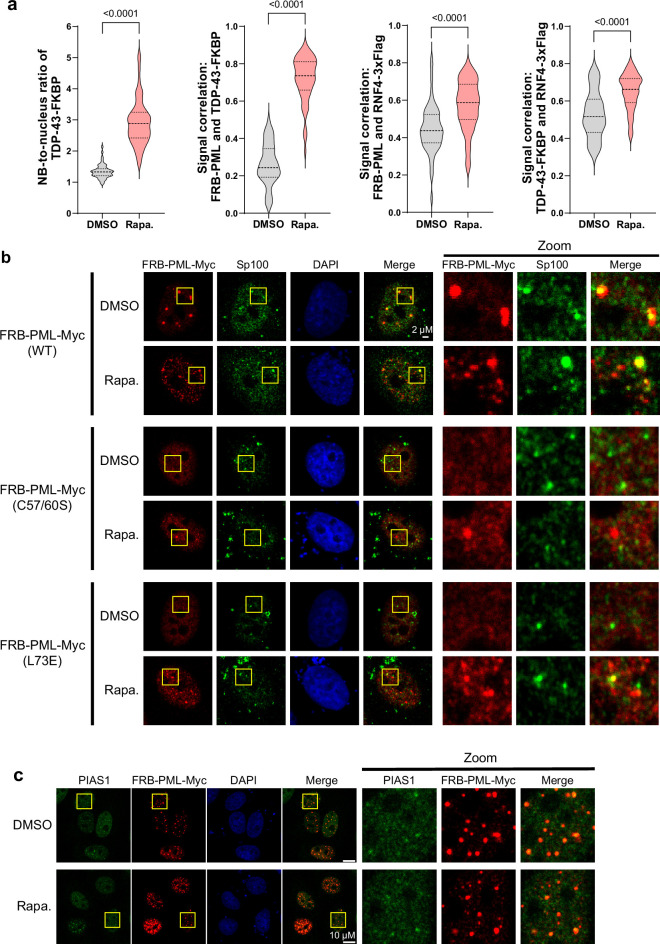
Recruitment of HA-TDP-43-FKBP to PML NBs. **a**, Statistical analysis of the immunofluorescence images shown in Fig. 3c. Enrichment of HA–TDP-43–FKBP in Myc-positive PML nuclear bodies (leftmost plot) and the correlation of the indicated signal pairs within the nucleus were analyzed. P values of two-tailed, unpaired Student’s t-tests are shown. At least 55 cells were measured per condition. **b**, HeLa cells were transfected with WT FRB–PML–Myc (WT V57,60S or L73E) and HA–TDP-43–FKBP for 24 h and either treated DMSO or rapamycin (100 nM, 4 h). Insets are marked with yellow squares. Scale bar, 2 µm. **c**, HeLa cells were transfected with WT FRB–PML–Myc and HA–TDP-43–FKBP for 24 h and either treated with DMSO or rapamycin (100 nM, 4 h). FRB–PML–Myc and PIAS1 were detected with anti-Myc and anti-PIAS1 antibodies and are shown in red and green, respectively. Inset areas are indicated with yellow squares. Scale bar, 10 µm. Source data

**Extended Data Fig. 4:**
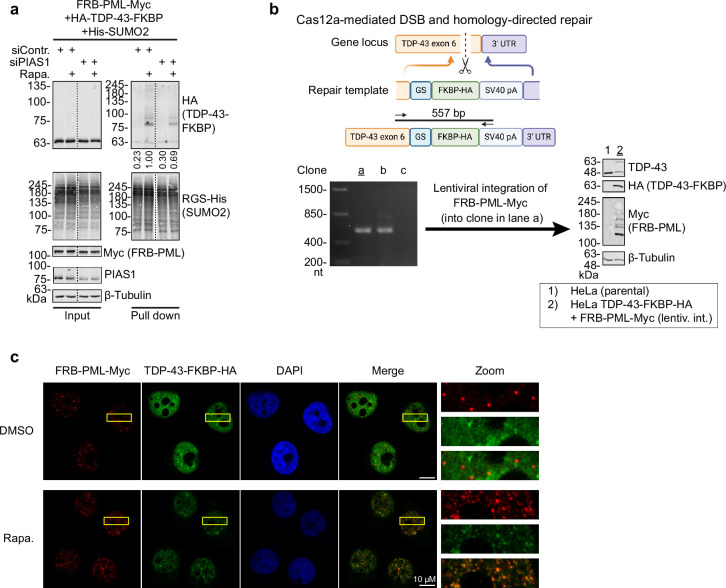
Recruitment of endogenously FKBP-tagged TDP-43 to PML NBs. **a**, Enrichment of SUMOylated HA–TDP-43–FKBP by Ni-NTA pull down. HEK293T cells were depleted of PIAS1 by siRNA transfection or transfected with a control siRNA. Cells were then transfected with FRB–PML–Myc, HA–TDP-43–FKBP and His-SUMO2. Signal intensities of high-molecular-weight bands (running above the height of unmodified HA–TDP-43VFKBP) in pull-down samples (anti-HA) were quantified and normalized to the respective intensities in the matching input lane. Relative values are listed below the top right panel. **b**, Top: HeLa cells were edited to express TDP-43–FKBP–HA from the endogenous gene locus. A double-strand break was introduced at the end of the last exon of TDP-43 using the CRISPR/LbCas12a system. A repair template harboring an FKBP12–HA and a puromycin resistance cassette (not shown in model) was then integrated by homologous recombination. Arrows indicate PCR primers used for the validation of successful editing events. Bottom left: genomic DNA was isolated and amplified with the indicated primers. PCR products were analyzed by agarose gel electrophoresis. The clone shown in lane **a** was transduced with lentiviral particles to stably express FRB–PML–Myc. The clone in lane **b** was excluded due to an additional (ca. 800 bp) PCR product. Parental HeLa cells were analyzed in lane **c**. Bottom right: expression of TDP-43–FKBP–HA and FRB–PML–Myc was assayed by western blotting. Parental HeLa cells in lane 1 served as a control. TDP-43–FKBP–HA was detected with both anti-HA and anti-TDP-43 antibodies at ca. 63 kDa. (Detection of WT TDP-43 at ca. 43 kDa with the anti-TDP-43 antibody indicated the presence of ca. 50% unedited TDP-43 alleles.). Panel **b** is created with BioRender.com. **c**, Expression of TDP-43–FKBP–HA and FRB–PML–Myc and rapamycin-induced recruitment of TDP-43 in the edited cells described in **b** were analyzed by immunofluorescence microscopy. Cells were either treated with DMSO or rapamycin (100 nM, 4 h). Scale bar, 10 µM. Source data

To expand on the abovementioned findings and to explore the potential solubility-promoting effect of TDP-43 SUMOylation–ubiquitylation in the context of PML NBs, we aimed to establish an inducible system that triggers recruitment of TDP-43 to PML. To this end, we chose a proximity-inducing approach that relies on the FKBP–FRB (FK506-binding protein and the FKBP-rapamycin binding domain of mTOR) protein pair. The association of FKBP–FRB can be induced by rapamycin, thus providing a robust tool to trigger binary molecular encounters within cells (Fig. 3a). To exploit this system, FRB (the rapamycin binding domain of mTOR) was fused to the N terminus of Myc-tagged PML and FKBP to the C-terminus of hemaglutinin-tagged (HA) TDP-43. Upon co-expression and addition of rapamycin, both proteins co-immunoprecipitate and exhibit colocalization (Fig. 3b,c and Extended Data Fig. 3a). Triple labeling also reveals costaining of the NB marker protein RNF4 upon rapamycin induction to both PML and TDP-43 as indicated by the enhanced signal correlation (Fig. 3c and Extended Data Fig. 3a). The rapamycin-induced PML foci also contain Sp100 and the E3 SUMO ligase PIAS1 (Extended Data Fig. 3b,c). Furthermore, rapamycin-mediated recruitment of TDP-43 to PML triggers its poly- or multi-SUMOylation as evidenced by the enrichment of multiple high-molecular-weight SUMO2–TDP-43 conjugates in nickel-nitrilotriacetic acid (Ni-NTA) precipitates of co-expressed His-tagged SUMO2 (Fig. 3d). Notably, addition of rapamycin stimulates SUMOylation of TDP-43 under basal conditions and further enhances its stress-induced modification. Notably, siRNA-mediated depletion of the SUMO ligase PIAS1 diminishes PML-induced SUMOylation of TDP-43, suggesting that PML engages the activity of PIAS ligases on TDP-43 within NBs (Extended Data Fig. 4a). PML is a member of the tripartite motif (TRIM) protein family comprising a RING finger domain, two B-box zinc finger domains and a coiled-coil region^40^. To see whether PML-induced SUMOylation of TDP-43 involves its NB localization, we generated two variants of PML, which do not properly form NBs^41,42^. In the PML^C57, 60S^ variant, two critical zinc-coordinating cysteine residues within the RING finger are replaced by serine residues, thereby disrupting RING integrity and NB localization of PML^41^. The PML^L73E^ variant is unable to engage RING tetramerization, a prerequisite for NB formation^42^. Both variants fail to properly localize to NBs and are strongly compromised in their activity to trigger SUMOylation of TDP-43 in the presence of rapamycin (Fig. 3e and Extended Data Fig. 3b).

To explore whether recruitment to PML also affects SUMOylation of endogenous TDP-43, we endogenously tagged TDP-43 with an FKBP–HA-tag in a cell line that stably expresses FRB–Myc–PML (Extended Data Fig. 4b,c). In this system, SUMO2 conjugates were detected in HA immunoprecipitates of rapamycin-treated cells, but not untreated controls, indicating that targeting endogenous TDP-43 to PML enhances TDP-43 SUMOylation (Fig. 3f). Notably, in PML knockout cells, the heat-induced SUMOylation of endogenous TDP-43 is significantly reduced, indicating that PML-regulated SUMOylation of TDP-43 is also relevant under physiological conditions (Fig. 3g).

### Recruitment to PML enhances ubiquitylation of TDP-43

**Fig. 4:**
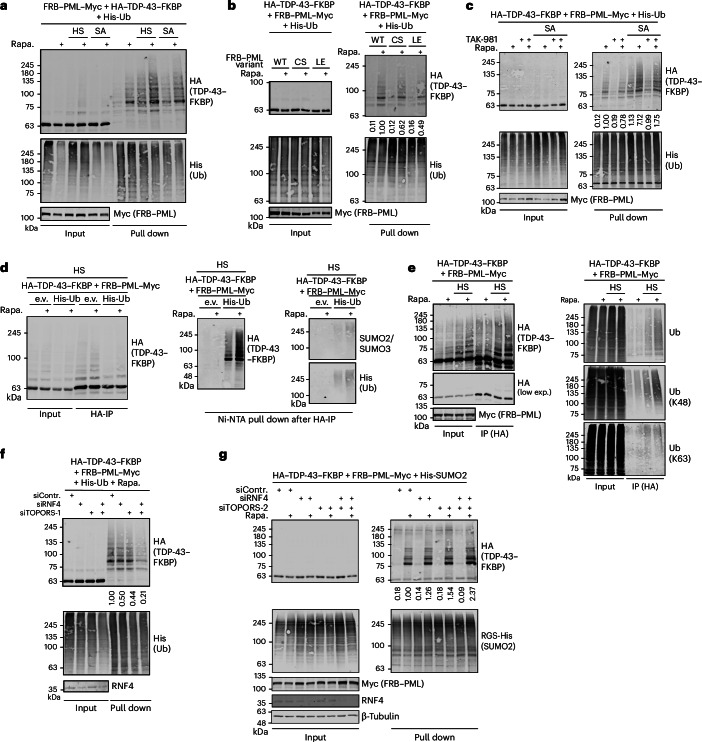
Recruitment to PML enhances ubiquitylation of TDP-43. **a**, Enrichment of ubiquitylated HA–TDP-43–FKBP by Ni-NTA pull down. As shown in Fig. 3d, but His-Ub was transfected together with FRB–PML–Myc and HA–TDP-43–FKBP. **b**, Enrichment of ubiquitylated HA–TDP-43–FKBP by Ni-NTA pull down in HEK293T cells expressing either WT or mutant (C57, 60S, L73E) FRB–PML–Myc together with HA–TDP-43–FKLBP and His-Ub. Signal intensities of high-molecular-weight bands (running above unmodified HA–TDP-43–FKBP) in pull-down samples (anti-HA) were quantified and normalized to the respective input intensities. Relative values are listed. **c**, Enrichment of ubiquitylated HA–TDP-43–FKBP by Ni-NTA pull down. HEK293T cells were transfected with His-Ub together with FRB–PML–Myc and HA–TDP-43–FKBP for 48 h and mock-treated or treated with TAK-981 (500 nM, 4 h). Rapamycin was added where indicated (100 nM, 4 h). Quantification was done as described in **b**. **d**, Enrichment of SUMO/Ub comodified HA–TDP-43–FKBP by HA-IP under denaturing conditions followed by Ni-NTA-pull down after HS (43 °C, 1 h). HEK293T cells were transfected with FRB–PML–Myc and HA–TDP-43–FKBP together with either His-Ub or an empty vector (e.v.). Rapamycin was added where indicated (100 nM, 4 h). HA–TDP-43–FKBP was enriched by HA-IP followed by denaturing Ni-NTA pull down of precipitated proteins. **e**, HEK293T cells were transfected with FRB–PML–Myc and HA–TDP-43–FKBP and treated with 100 nM rapamycin or DMSO for 4 h. TDP-43–FKBP was enriched by a denaturing HA-IP, and the ubiquitylation of TDP-43–FKBP was monitored with a pan-Ub or Ub-chain-specific (K48, K63) antibodies. **f**, Enrichment of ubiquitylated HA–TDP-43–FKBP by Ni-NTA pull down. HEK293T cells depleted of RNF4, Topors or both by siRNA or transfected with a nontargeting control siRNA followed by transfection with His-Ub, FRB–PML–Myc and HA–TDP-43–FKBP. Before lysis, cells were treated with rapamycin (100 nM, 4 h) or DMSO. Quantification was done as described in **b**. **g**, Enrichment of SUMOylated HA–TDP-43–FKBP by Ni-NTA pull down. As in **f**, but His-SUMO2 was transfected instead of His-Ub. Quantification was done as described in **b**. Source data

**Extended Data Fig. 5:**
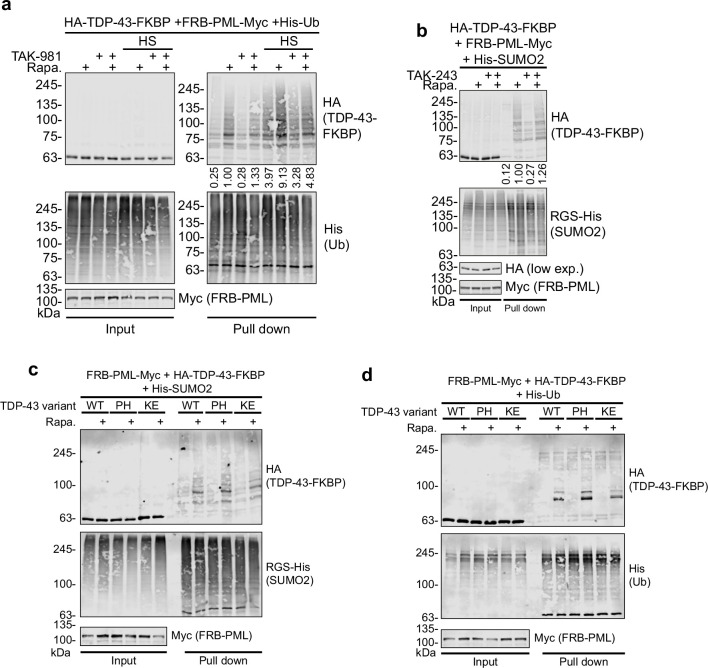
Recruitment to PML enhances SUMOylation and ubiquitylation of disease associated TDP-43 mutants. **a**, Enrichment of ubiquitylated HA–TDP-43–FKBP by Ni-NTA pull down. HEK293T cells were transfected with His-Ub together with FRB–PML–Myc and HA–TDP-43–FKBP. Cells were treated with heat stress (43 °C, 1 h) or left untreated. Where indicated, cells were treated with rapamycin (100 nM, 4 h) and TAK-981 (500 nM, 4 h). Signal intensities of high-molecular-weight bands (running above the height of unmodified HA–TDP-43–FKBP) in pull-down samples (anti-HA) were quantified and normalized to the respective intensities in the matching input lane. Relative values are listed below the top right panel. **b**, Enrichment of SUMOylated HA–TDP-43–FKBP by Ni-NTA pull down. HEK293T cells were transfected with His-SUMO2 together with FRB–PML–Myc and HA–TDP-43–FKBP. Where indicated, cells were treated with rapamycin (100 nM, 4 h) and TAK-243 (10 µm, 4 h). Relative intensities of high-molecular-weight TDP-43–FKBP were determined as described in **a**. **c**, Enrichment of SUMOylated HA–TDP-43–FKBP by Ni-NTA pull down. HEK293T cells were transfected with His-SUMO2 together with FRB–PML–Myc and HA–TDP-43–FKBP (WT, P112H or K263E). Where indicated, cells were treated with rapamycin (100 nM, 4 h). **d**, Enrichment of ubiquitylated HA–TDP-43–FKBP by Ni-NTA pull down. HEK293T cells were transfected with His-Ub together with FRB–PML–Myc and HA–TDP-43–FKBP (WT, P112H or K263E). Where indicated, cells were treated with rapamycin (100 nM, 4 h). Source data

We next asked whether enhanced SUMOylation is associated with an increase in ubiquitylation. To this end, His-tagged ubiquitin was co-expressed with FRB–PML and FKBP–TDP-43, and ubiquitin–TDP-43 conjugates were captured by Ni-NTA pull down and detected by anti-HA immunoblotting (Fig. 4a). Similar to what we observed for SUMOylation of TDP-43, rapamycin-mediated binding of PML to TDP-43 enhances TDP-43 ubiquitylation under basal and stress-induced conditions (Fig. 4a). The PML^C57, 60S^ and the PML^L73E^ variants, which do not form proper PML NBs, are compromised in their ability to induce ubiquitylation of TDP-43 in the presence of rapamycin, pointing to a SUMO-regulated ubiquitylation process (Fig. 4b). In line with this idea, inhibition of SUMOylation with the highly specific E1 inhibitor, TAK-981, limits rapamycin-induced ubiquitylation of TDP-43 upon exposure to heat or arsenite stress, suggesting that PML-induced SUMOylation stimulates the ubiquitylation of TDP-43 (Fig. 4c and Extended Data Fig. 5a). Notably, inhibition of SUMOylation does not completely abrogate ubiquitylation, indicating that forced recruitment to PML can at least partially foster ubiquitylation of TDP-43 in a SUMOylation-independent manner. Treatment of cells with the ubiquitin E1 inhibitor TAK-243 induces basal and rapamycin-induced SUMOylation of TDP-43, further indicating that both modifications are interconnected (Extended Data Fig. 5b). To explore whether recruitment to PML generates TDP-43 forms that are comodified by SUMO2 and ubiquitin, we performed tandem purification of ubiquitylated TDP-43 species. To this end, HA–TDP-43–FKBP was co-expressed with FRB–PML–Myc and His-Ub. TDP-43 was immunoprecipitated by denaturing anti-HA-IP, eluted from HA beads and His-ubiquitin–TDP-43 species were enriched by Ni-NTA pull down. This material was probed by anti-SUMO2/SUMO3 immunoblotting for the presence of endogenous SUMO2/SUMO3. In this experimental setup, we could detect more SUMO2/SUMO3 on ubiquitylated TDP-43 species upon rapamycin treatment, indicating that TDP-43 recruitment of PML fosters comodification by ubiquitin and SUMO at separate lysine residues or by the formation of hybrid SUMO-Ub chains (Fig. 4d). Using chain-specific antibodies, we detected both K48- and K63-linked ubiquitin chains in HA immunoprecipitates (Fig. 4e). To interrogate the involvement of RNF4 or Topors in the ubiquitylation of TDP-43, we compared rapamycin-induced ubiquitylation of TDP-43 in control cells and cells depleted either individually of RNF4 or Topors or codepleted of both proteins. In this setting, Ni-NTA pull-down experiments reveal a drastic impairment of ubiquitylation upon codepletion of RNF4 and Topors, indicating that both StUbLs contribute to SUMO-primed ubiquitylation of TDP-43 (Fig. 4f). In agreement with this finding, codepletion of RNF4 and Topors leads to a significant stabilization of SUMO2–TDP-43 conjugates, a characteristic feature for SUMO-primed ubiquitylation targets (Fig. 4g)^26,43^. We next asked whether recruitment to PML can also affect the modification of ALS-associated mutants of TDP-43. We chose FKBP-tagged TDP-43^P112H^ or TDP-43^K263E^, which are both devoid of RNA binding and were described to phase-separate into nuclear condensates^9^. Both variants were chosen because preliminary data indicate that they exhibit enhanced SUMOylation. When co-expressed with FRB–PML and His-Ub/SUMO, both mutants, like the WT protein, exhibit enhanced SUMOylation as well as ubiquitylation in the presence of rapamycin (Extended Data Fig. 5c,d). Altogether, these data show that PML can be exploited to trigger a SUMOylation–ubiquitylation cascade on TDP-43 and ALS-associated TDP-43 variants in a process that depends on NB localization.

### Recruitment to PML limits the aggregation of TDP-43

**Fig. 5:**
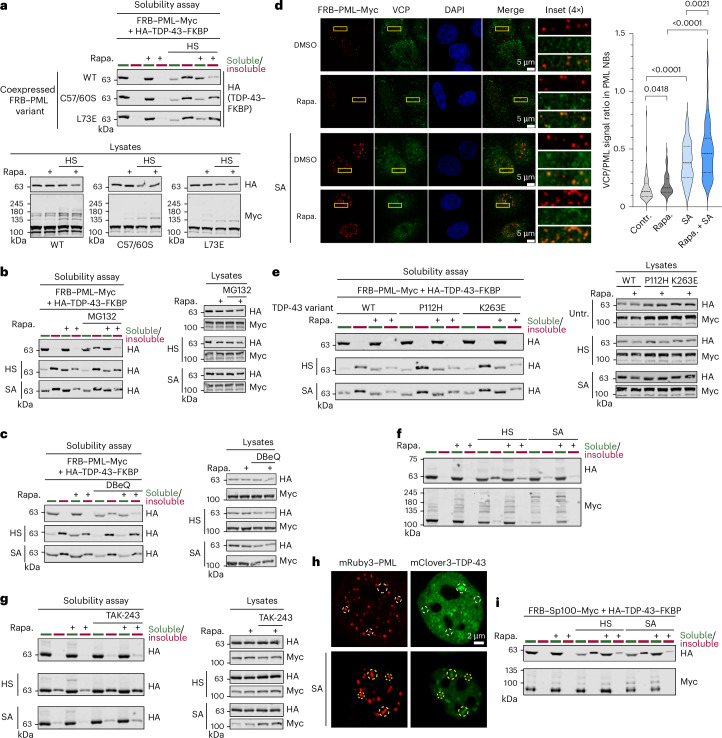
Recruitment to PML limits the aggregation of TDP-43. **a**, Top, solubility assay. HEK293T cells were transfected as indicated for 48 h, exposed to HS (43 °C, 1 h) or left untreated. Where indicated, rapamycin was added (100 nM, 4 h). Bottom, unfractionated lysates. Statistical analysis is provided in Extended Data Fig. 6a (*n* = 3). **b**, Left, solubility assay as in **a**, but cells were treated with MG132 (25 µm, 4 h). Right, unfractionated lysates. Statistical analysis is provided in Extended Data Fig. 6b (*n* = 3). **c**, Left, solubility assay as in **a** and **b**, but cells were treated with DBeQ (20 µm, 4 h). Right, unfractionated lysates. Statistical analysis is provided in Extended Data Fig. 6c (HS, *n* = 5; SA, *n* = 4). **d**, Left, immunofluorescence images of pre-extracted HeLa cells transfected with FRB–PML–Myc and HA–TDP-43–FKBP. Treatment with rapamycin (100 nM, 4 h), DMSO and SA (0.5 mM, 1 h) where indicated. Scale bar, 5 µm. Right, VCP/FRB-signal ratio in PML NBs was determined per cell. At least 71 cells per condition were measured. *P* values of two-tailed, unpaired Student’s *t* tests are indicated. **e**, Left, solubility assay as in **a**–**c**, but cells were expressing ALS-associated TDP-43 variants as indicated. Right, unfractionated lysates. Statistical analysis is provided in Extended Data Fig. 6d (*n* = 3). **f**, Solubility assay in engineered HeLa cells expressing TDP-43–FKBP–HA from the endogenous TDP-43 locus together with FRB–PML–Myc. Treatments—rapamycin (100 nM, 4 h), HS (43 °C, 1 h) and SA (0.5 mM, 1 h). Statistical analysis is provided in Extended Data Fig. 6e (*n* = 3). **g**, Left, solubility assay as in **f**, but where indicated cells were treated with TAK-243 (10 µm, 4 h). Right, unfractionated lysates. Statistical analysis is provided in Extended Data Fig. 7a (*n* = 3). **h**, Fluorescence microscopy. mRuby3–PML and mClover3–TDP-43 expression from a lentiviral expression cassette (HeLa) was induced by doxycycline (1 µg ml^−1^, 24 h). Colocalizing and noncolocalizing foci of mRuby3–PML and mClover3–TDP-43 are indicated by yellow and white circles, respectively. Scale bar, 2 µm. Statistical analysis is provided in Extended Data Fig. 7b. **i**, Solubility assay as in **a**, but in HEK293T cells expressing FRB–Sp100–Myc (instead of FRB–PML–Myc) and HA–TDP-43–FKBP for 48 h. Statistical analysis is provided in Extended Data Fig. 8c (*n* = 3). Untr., untreated. Source data

**Extended Data Fig. 6:**
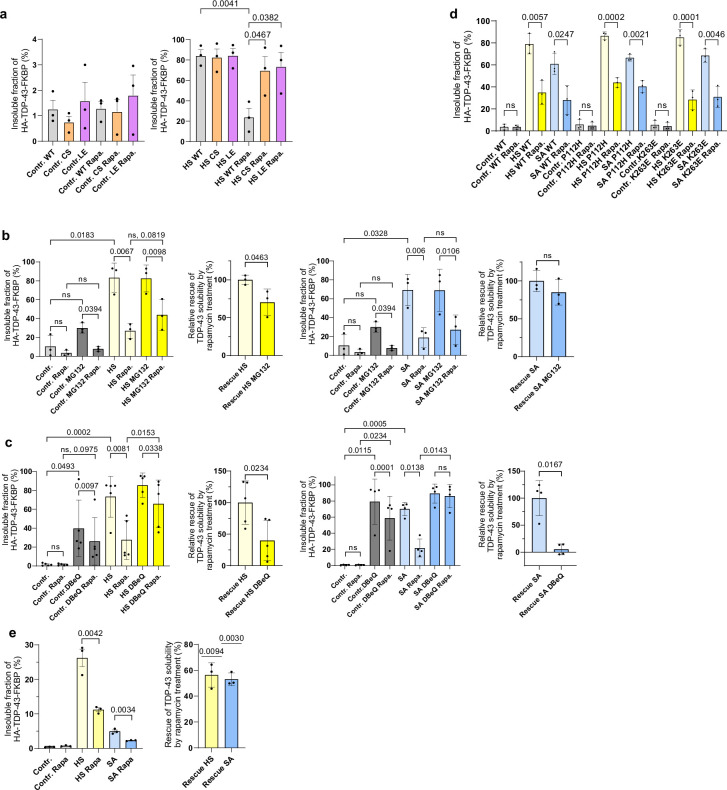
Statistical analysis of the protective role of TDP-43 recruitment into PML NBs against stress-induced TDP-43 insolubility. **a**, Statistical analysis of HA–TDP-43–FKBP insolubility. The insoluble fraction of HA–TDP-43–FKBP (in % of total HA–TDP-43–FKBP) was determined in solubility assays, as shown in Fig. 5a. P values of two-tailed, unpaired Student’s t-tests are indicated; error bars show the standard deviation of the mean (n = 3). **b**, Statistical analysis of HA–TDP-43–FKBP insolubility. First and third graphs: the insoluble fraction of HA–TDP-43–FKBP (in % of total HA–TDP-43–FKBP) was determined in solubility assays as shown in Fig. 5b. Second and fourth graphs: the relative decrease in insoluble HA–TDP-43–FKBP upon treatment with rapamycin (=rescue) in samples treated with DMSO or MG132 is shown. The mean rescue in DMSO-treated cells was set to 100%. P values of two-tailed, paired Student’s t-tests are indicated; error bars show the standard deviation of the mean (n = 3). **c**, Statistical analysis of HA–TDP-43–FKBP insolubility as in **b**, but samples were treated with DBeQ instead of MG132. Solubility assays were performed as shown in Fig. 5c. P values of two-tailed, paired Student’s t-tests are indicated; error bars show the standard deviation of the mean (HS, n = 5; SA, n = 4). **d**, Statistical analysis of HA–TDP-43–FKBP (WT, P112H and K263E) insolubility. The insoluble fraction of HA–TDP-43–FKBP variants (in % of total HA–TDP-43–FKBP) was determined in solubility assays, as shown in Fig. 5e. P values of two-tailed, unpaired Student’s t-tests are indicated; error bars show the standard deviation of the mean (n = 3). **e**, Statistical analysis of TDP-43–FKBP–HA insolubility. Assays as shown in Fig. 5f were performed with HeLa cells engineered to express TDP-43–FKBP–HA from the endogenous TDP-43 locus and transduced with lentiviral particles to stably express FRB–PML–Myc. The insoluble fraction of HA–TDP-43–FKBP variants (in % of total TDP-43–FKBP–HA) was determined. P values of two-tailed, unpaired (left) and one-sample (right) Student’s t-tests are indicated; error bars show the standard deviation of the mean (n = 3). Source data

**Extended Data Fig. 7:**
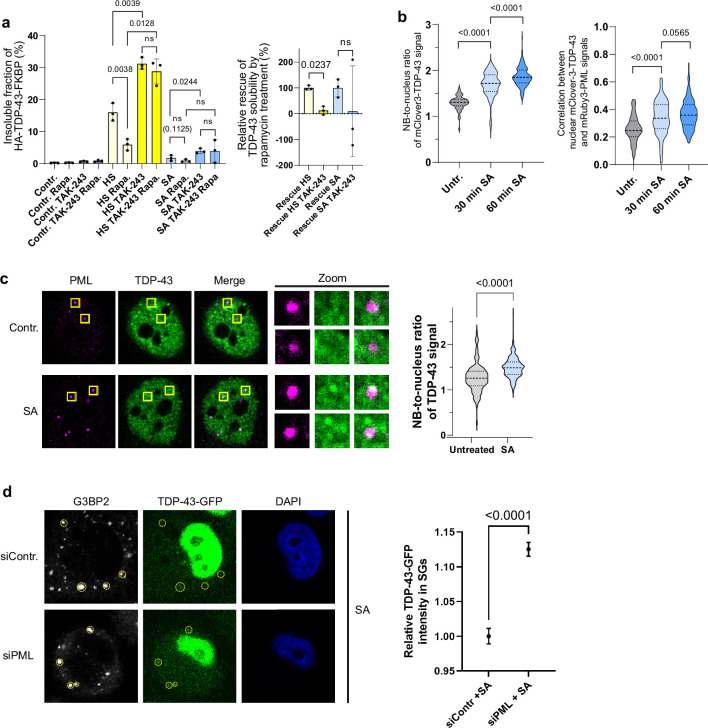
Stress enhances TDP-43 association with PML NBs and stress granules, with increased TDP-43 in stress granules upon PML depletion. **a**, Statistical analysis of HA–TDP-43–FKBP insolubility assays as shown in Fig. 5g with HeLa cells engineered to express TDP-43–FKBP–HA from the endogenous TDP-43 locus and FRB–PML–Myc from a lentiviral expression cassette. Treatments: heat stress (43 °C, 1 h), sodium arsenite (0.5 mM, 1 h) or no stress treatment combined with rapamycin (100 nM, 4 h) or DMSO. P values of two-tailed, unpaired Student’s t-tests are indicated (n = 3). **b**, Statistical analysis of the enrichment of mClover3–TDP-43 in PML NBs in HeLa cells as shown in Fig. 5h. Cells were treated with sodium arsenite (0.5 mM, 30 or 1 h) or left untreated. Left: the mean intensity of the TDP-43–mClover3 within the area PML NBs was divided by the mean intensity within the nucleus of the same cell. Right: correlation between mClover3–TDP-43 and mRuby3–PML within the nucleus of transfected cells. At least 65 cells per condition were analyzed. P values of two-tailed, unpaired Student’s t-tests are indicated. **c**, Left: immunofluorescence images of endogenous PML and TDP-43. HeLa cells were left untreated or treated with sodium arsenite (0.5 mM, 1 h). Inset areas are indicated. Right: statistical analysis of the TDP-43 signal enrichment within PML NBs upon sodium arsenite treatment. P values of two-tailed, unpaired Student’s t-tests are indicated. At least 120 cells per condition were analyzed. **d**, Immunofluorescence images of G3BP2 in HeLa cells stably expressing TDP-43–GFP. Cells were transfected with control siRNA or with an siRNA directed against PML for 48 hours. Treatment: sodium arsenite (0.5 mM, 1 h). Left: yellow circles indicate large stress granules. Right: the relative intensity of TDP-43–GFP within the area of SGs was determined and normalized to the mean value in cells transfected with control siRNA. At least 96 cells/condition were analyzed. Error bars indicate the standard error of the mean. The p value of a two-tailed, unpaired Student’s t-test is indicated. Source data

**Extended Data Fig. 8:**
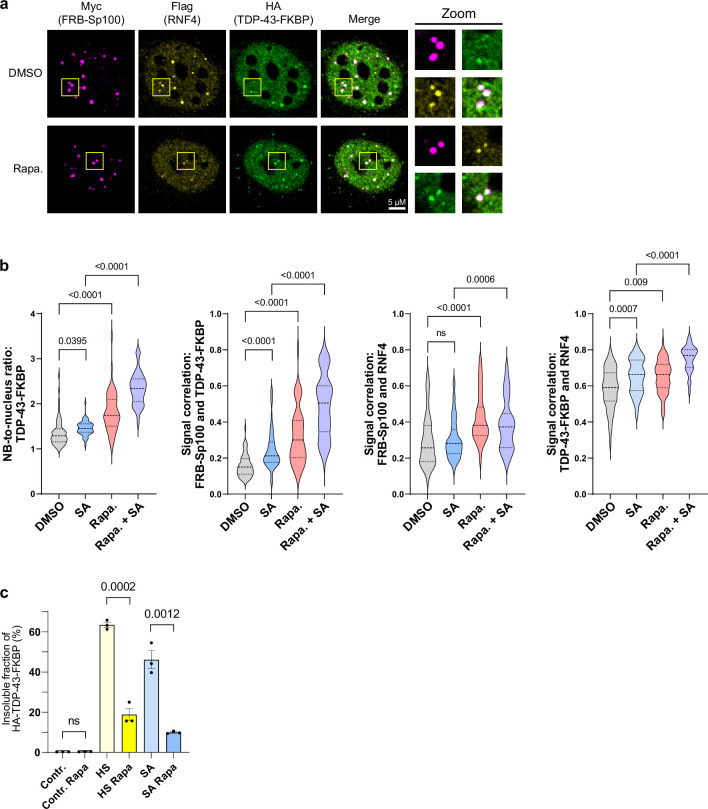
Recruitment to the PML NB core component Sp100 protects TDP-43 from stress-induced aggregation. **a**, HeLa cells were genetically engineered to express RNF4-3×Flag from the endogenous RNF4 locus. Cells were transfected with FRB–Sp100–Myc and HA–TDP-43–FKBP for 24 h and either mock-treated or treated with rapamycin (100 nM, 3 h). Inset areas are indicated with yellow squares. Scale bar, 5 µm. **b**, Statistical analysis of the images shown in **a**. Enrichment of HA–TDP-43–FKBP in FRB–Sp100–Myc-positive nuclear bodies (leftmost plot) and the correlation of the indicated signal pairs within the nucleus were analyzed. At least 55 cells per condition were measured. The p values of two-tailed, unpaired Student’s t-tests are indicated. **c**, Statistical analysis of HA–TDP-43–FKBP insolubility in HEK293T cells transfected with HA–TDP-43–FKBP and FRB–Sp100–Myc. The insoluble fraction of HA–TDP-43–FKBP (in % of total HA–TDP-43–FKBP) was determined in solubility assays, as shown in Fig. 5i. P values of two-tailed, unpaired Student’s t-tests are indicated; error bars show the standard deviation of the mean (n = 3). Source data

Based on our finding that fusion of SUMO2 limits the aggregation of TDP-43 (Fig. 1), we were intrigued by the question of whether PML-induced SUMOylation–ubiquitylation of TDP-43 can recapitulate these effects. To address this point, we monitored the solubility of TDP-43 in HEK293T cells co-expressing FRB–PML and FKBP–TDP-43 upon exposure to heat stress or arsenite (Fig. 5a and Extended Data Fig. 6a). In the absence of rapamycin, heat stress induces the shift of 80% of total TDP-43 from the soluble to the insoluble fraction. However, this can be largely reversed to 20% of TDP-43 in the insoluble fraction upon treatment of cells with rapamycin. Notably, the RING-deficient PML variants that cannot properly form NBs and are compromised in SUMOylation/ubiquitylation of TDP-43 largely fail to prevent its accumulation in the insoluble fraction (Fig. 5a), suggesting a direct link between ubiquitylation and enhancement of solubility. Notably, the inability of the RING mutants to shield TDP-43 from insolubility in the presence of rapamycin also rules out that the protective effect is caused by the addition of rapamycin. To determine whether proteasome or p97 activity is involved in the PML-mediated protection of TDP-43 from aggregation, cells were treated with the proteasome inhibitor MG132 or the p97 inhibitor DBeQ. Upon proteasome inactivation, rapamycin-induced recruitment of TDP-43 to PML still limits the accumulation of TDP-43 in the insoluble fraction. Notably, however, proteasome inhibition diminishes the protective effect of PML-induced ubiquitylation under heat stress, suggesting that both proteolytic and nonproteolytic ubiquitylation events are involved in solubility enhancement under these conditions (Fig. 5b and Extended Data Fig. 6b). Compared to MG132, DBeQ more drastically compromises the protective effect of PML recruitment on TDP-43 under both heat and arsenite stress (Fig. 5c and Extended Data Fig. 6c). In line with this finding, rapamycin-induced recruitment of TDP-43 to PML enhances colocalization of p97 with PML NBs under both basal and stress conditions (Fig. 5d). PML also protects the pathogenic ALS-associated variants TDP-43^P112H^ or TDP-43^K263E^ from aggregation, which become highly insoluble under stress in the absence of rapamycin but can be kept in the soluble fraction in the presence of rapamycin (Fig. 5e and Extended Data Fig. 6d). To expand on these findings, we aimed to validate whether proximity-induced recruitment to PML can limit the aggregation of endogenously FKBP–HA-tagged TDP-43 in the cellular system described above (Extended Data Fig. 4b). Notably, rapamycin-induced FRB–PML recruitment reduces the amount of endogenously tagged TDP-43–FKBP–HA in the insoluble fraction in response to heat or arsenite stress (Fig. 5f and Extended Data Fig. 6e). Treatment of cells with the ubiquitin E1 inhibitor TAK-243 almost completely abolishes the ability of PML to protect cells from TDP-43 aggregates in response to either heat or arsenite-induced stress, validating the importance of ubiquitylation for protecting TDP-43 aggregation (Fig. 5g and Extended Data Fig. 7a). Altogether, our data indicate that proximity-induced recruitment to PML protects TDP-43 from stress-triggered insolubility. To further explore whether TDP-43 associates with PML NBs in the course of a physiological stress response, cells stably expressing mClover3–TDP-43 and mRuby3–PML were exposed to arsenite and localization of both proteins was followed by confocal fluorescence imaging. While in unstressed cells TDP-43 is only occasionally found in association with PML NBs, a significant fraction of TDP-43 is recruited to PML NBs upon exposure to arsenite (Fig. 5h and Extended Data Fig. 7b). Upon exposure of cells to arsenite, a subfraction of endogenous TDP-43 foci can also be detected in or directly adjacent to PML NBs (Extended Data Fig. 7c). Although antibody-based costaining of both proteins under endogenous expression levels is far less pronounced than colocalization of the fluorescence-tagged proteins at elevated expression, the data support the idea that PML NBs contribute to the protection of TDP-43 from aggregation. In line with this idea, the knockdown of PML slightly but significantly induces the stress-induced relocalization of TDP-43 to SGs (Extended Data Fig. 7d). To strengthen the idea that PML NBs represent a safe haven for TDP-43, we determined whether proximity-induced recruitment of TDP-43 to Sp100, another NB core component, recapitulates the effects observed by PML recruitment. Rapamycin induced the colocalization of TDP-43 with Sp100 and the NB marker RNF4 (Extended Data Fig. 8a,b). Strikingly, this also limits the accumulation of TDP-43 in the insoluble fraction in response to both heat and arsenite stress (Fig. 5i and Extended Data Fig. 8c). Altogether, these data indicate that recruitment to NBs in conjunction with a SUMOylation–ubiquitylation cascade exerts a protective role against TDP-43 insolubility and hence might limit TDP-43 inclusions.

## Discussion

**Fig. 6:**
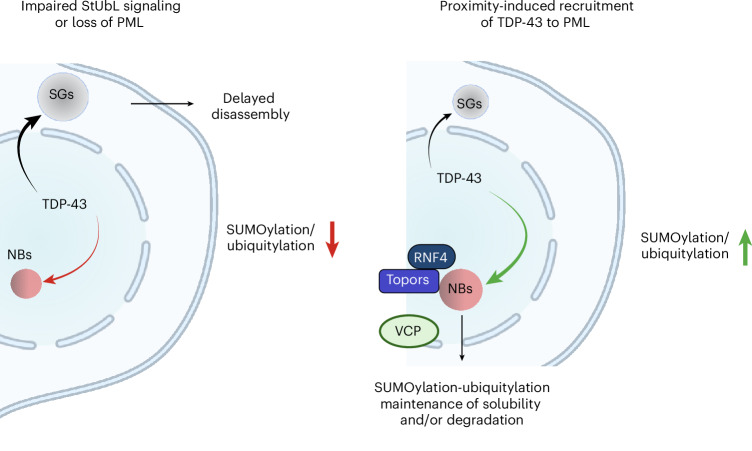
Model. Model depicting the interconnection of StUbL signaling and PML NBs with cytosolic SGs in proteostasis of TDP-43. The figure is created with BioRender.com.

Cytosolic and nuclear aggregates of TDP-43 are associated with the pathogenesis of ALS, FTD and AD. Formation and clearance of these aggregates are controlled by an intricate interplay of protein quality control systems. Understanding the impact of these systems on TDP-43 is a prerequisite to exploiting these systems for developing rational therapeutic approaches limiting TDP-43 aggregation^44^. Here we provide compelling evidence that SUMO–ubiquitin networks that are spatially associated with PML NBs prevent TDP-43 aggregation upon proteotoxic stress and demonstrate that PML can be functionalized to induce a SUMOylation–ubiquitylation cascade on TDP-43 to limit its aggregation propensity in response to proteotoxic stress. We propose a model where PML NBs orchestrate StUbL signaling to protect cells from TDP-43 aggregates and accumulation in cytosolic SGs (Fig. 6). By adapting the concept of targeted degradation by proteolysis targeting chimeras (PROTACs) and molecular glues that rely on the harnessing of ubiquitin ligases, we propose that bifunctional molecules or molecular glues that recruit PML to TDP-43 can be used in the future to limit aggregation of TDP-43.

Previous work indicates that TDP-43 modification by SUMO1 at lysine 136 controls its splicing activity, nuclear-cytoplasmatic shuttling and the formation of cytoplasmic TDP-43 aggregates^19,20^. Here we concentrated on stress-induced SUMOylation by SUMO2, which targets multiple residues and leads to the formation of SUMO2 chains. Our data clearly show that polySUMOylation in conjunction with ubiquitylation limits the formation of TDP-43 aggregates, indicating that SUMO1 and SUMO2 may exert distinct effects on TDP-43. Notably, the major SUMO1 attachment site (K136) is also targeted by acetylation and resides in a critical structural position that does not tolerate conservative amino acid substitutions^15^, suggesting a somewhat cautious interpretation of data obtained with mutants harboring lysine to arginine mutations at K136. To explore the role of SUMO2 modification on TDP-43, we, therefore, opted for a different approach by either fusing tetra-SUMO chains directly to TDP-43 or by inducing SUMOylation through recruitment of PML. PML functions as the scaffold and organizer of PML NBs. These nuclear condensates, which are formed by liquid–liquid phase separation, have emerged as hubs for post-translational modifications and centers of nuclear protein quality control^32,45,46^. The current view is that aberrant or misfolded proteins, such as defective ribosomal products or extended polyQ proteins, are transiently sequestered in PML NBs, where they are handled by the cellular proteostasis machinery^32,37,38,45,47^. The crucial role of PML NBs in the maintenance of nuclear protein quality control is further supported by the observation that PML NBs are lost in cases of familial ALS/FTD^36^. Recent work proposed a transfer of TDP-43 between different stress-induced nuclear condensates, including PML, suggesting that these condensates exert overlapping functions in countering TDP-43 aggregation^6^. This likely explains why the loss of PML alone only moderately enhances TDP-43 aggregation.

There is accumulating evidence that the function of PML NBs as centers of protein quality control is tightly linked to their role as hubs of SUMOylation and ubiquitylation^46^. PML NBs serve as a platform for nuclear SUMOylation and SUMO-dependent protein–protein interactions. Multiple components of the SUMO machinery, including the E2 enzyme Ubc9, members of the PIAS family of E3 SUMO ligases^48^ and StUbLs (RNF4, RNF111 and Topors) are associated with PML NBs^49–51^. Furthermore, PML and most other core components of NBs, such as Daxx and Sp100, represent cellular targets for covalent modification by SUMO and also harbor SIMs for noncovalent SUMO binding^52^. Heterotypic multivalent interactions of SUMO-modified proteins with proteins containing SIMs have been proposed to drive the dynamics and composition of NBs^23,41,43,53^. In line with this idea, we demonstrate that the attachment of SUMO2 to TDP-43 enhances its SIM-dependent interaction with PML, thereby facilitating compartmentalization in NBs and driving StUbL-mediated ubiquitylation within these structures.

It remains to be determined whether PML itself or a NB-associated SUMO ligase catalyzes SUMOylation of TDP-43. As a member of the TRIM family, PML harbors a RING finger domain^54^. TRIM proteins typically exert RING-dependent ubiquitin ligase activity, but distinct TRIMs, including PML, have also been reported to stimulate SUMO conjugation^40^. Here we show that the proximity-induced recruitment of PML to TDP-43 induces SUMOylation of TDP-43 in cells in a RING-dependent manner, supporting the view that PML directly promotes SUMOylation of TDP-43. However, because the RING mutants are also defective in PML NB assembly, targeting TDP-43 to the PML-associated SUMO machinery, but not ligase activity of PML itself, could be the driver for TDP-43 SUMOylation. In support of this idea, PML-induced SUMOylation of TDP-43 is impaired upon depletion of the NB-associated SUMO E3 ligase PIAS1. Furthermore, NB targeting of TDP-43 by the NB core protein Sp100 can recapitulate the protective effects of PML, favoring the interpretation that the recruitment of TDP-43 to PML brings TDP-43 into a protective SUMOylation–ubiquitylation-prone environment. Our data suggest that recruitment of TDP-43 engages the NB resident SUMOylation–ubiquitylation machinery, thereby amplifying a SUMOylation–ubiquitylation cascade and generating TDP-43 species that are comodified by SUMO2 and ubiquitin. We provide evidence that the NB-associated StUbLs RNF4 and Topors cooperate in catalyzing SUMO-dependent ubiquitylation of TDP-43. Interestingly, however, inhibition of SUMOylation does not fully abrogate PML-triggered ubiquitylation, indicating that proximity-induced recruitment of TDP-43 to PML NBs can at least partially circumvent the SUMO priming step that typically precedes StUbL-mediated ubiquitylation. Notably, PML-induced ubiquitylation limits the accumulation of insoluble TDP-43 upon exposure to proteotoxic insults. Our finding that both K63- and K48-linked ubiquitin are detected at TDP-43 together with the observation that proteasome inhibition partially impairs the solubility enhancement by PML suggests that both proteolytic and nonproteolytic ubiquitylation events are involved in solubility enhancement. Because the protective effect of PML recruitment is lost upon inhibition of the AAA ATPase p97, we propose a model in which ubiquitin chains or hybrid SUMO2–ubiquitin chains on TDP-43 functionalize the disaggregase activity of p97. p97 has a well-established function in protecting cells from cytotoxic aggregates, including TDP-43 inclusions, and, accordingly, p97 mutations are associated with ALS and FTD^55,56^. Typically, p97 functions as part of a protein segregase complex by a network of ubiquitin-binding adaptor molecules, such as the NPL4/UFD1 heterodimer. Work on Cdc48, the yeast counterpart of p97, has demonstrated that the Ufd1–Npl4–Cdc48 complex in yeast harbors binding modules for ubiquitin and SUMO, thereby connecting both modification pathways for protein degradation or other regulatory purposes^57,58^. Furthermore, it has been observed that the Ufd1–Npl4–Cdc48 complex in yeast prioritizes substrates dually modified with SUMO and polyubiquitin over substrates containing only polyubiquitin^59^. It is tempting to speculate that the combinatorial ubiquitin–SUMO signal that is generated on TDP-43 by its recruitment to PML engages the p97 machinery to TDP-43 in degradative or nondegradative pathways. In line with this idea, polySUMO-primed ubiquitylation of DNA repair factors triggers their extraction from chromatin by p97 (ref. ^28^). Notably, SUMO1 modification has recently been shown to cooperate with p97 in the clearance of a misfolded cytosolic model substrate, indicating a division of labor between SUMO1 and SUMO2/SUMO3 in spatial protein quality control^60^. We propose that hybrid ubiquitin–SUMO2 chains expand the repertoire of nuclear ubiquitin signaling events that orchestrate TDP-43 proteostasis in response to distinct signals^61^. Notably, we monitored the fate of SUMOylated/ubiquitylated TDP-43 at early time points after rapamycin/stress treatment, where p97 primarily exerts its diaggregase activity, while degradation might follow at the later time points. In addition to the scenario discussed here, p97 may also contribute to the removal of TDP-43 aggregates through its well-established role in autophagy^55^.

Altogether, our findings show that PML can be functionalized to trigger a SUMOylation–ubiquitylation cascade that activates the p97 disaggregase pathway or other protein quality control systems, such as molecular chaperones. Reminiscent of our findings, recent work has reported that the TRIM family member TRIM11 protects cells from tau aggregates^59^. TRIM11 promoted the proteasomal degradation of mutant tau in a SUMO–ubiquitin-dependent manner. Furthermore, TRIM11 enhanced tau solubility by acting as a molecular chaperone and a disaggregase. Based on these findings, we propose that PML (also known as TRIM19) or other TRIMs can be functionalized for the activation of disaggregase pathways to limit the formation of pathological inclusions in neurodegenerative disease. We anticipate that this expands the repertoire of Ub-/UbL-based therapeutics beyond PROTACs, which primarily target proteins for ubiquitin-mediated degradation.

## Methods

### Cell culture and cell line generation

HEK293T (female, American Type Culture Collection (ATCC) CRL-3216), HeLa (female, ATCC CCL-2) and U2OS (female, ATCC HTB-96) cells were purchased from ATCC and cultured in Dulbecco’s modified Eagle medium (DMEM) supplemented with 10% fetal calf serum (Gibco) and 1% penicillin–streptavidin mixture (10,000 U ml^−1^; Gibco).

The HeLa cell line stably expressing TDP-43–FKBP–HA (from the CRISPR–Cas12a-edited endogenous TDP-43 locus) in combination with FRB–PML–Myc (from a lentiviral transduced transgene) was generated as follows. Editing of the TDP-43 gene locus was performed following the protocol published in ref. ^62^. The Halo-Tag in a pMaCTag-P23 vector was replaced by a sequence encoding FKBP–HA. Primers (including homology to the repair site and the top-ranking guide RNA for LbCas12a) were designed using the online oligo design tool associated with the aforementioned protocol (http://pcr-tagging.com/). The repair cassette generated by PCR amplification was transfected together with an LBCas12a encoding helper plasmid. Twenty-four hours later, the cells were switched to the selection medium (DMEM containing 1 µg ml^−1^ puromycin). Single clones were expanded in 96-well plates, and recombination with the repair cassette was validated using PCR and western blotting. Next, lentiviral particles harboring FRB–PML–Myc and a hygromycin-resistance gene were transduced into the CRISPR–Cas12a-edited cells. Two days later, cells expressing FRB–PML–Myc were selected by changing the medium to the selection medium (DMEM containing 1 µg ml^−1^ puromycin and 200 µg ml^−1^ hygromycin B). Expression of FRB–PML–Myc was validated by western blotting.

The U2OS cell line harboring a PML knockout was created using the dual-guide RNA system described in ref. ^63^. Two SpyoCas9-specific guide RNA sequences targeting exons 3 and 5 of PML were selected using the CRISPick guide RNA design tool (portals.broadinstitute.org/gppx/crispick/public). The guide RNA sequences were cloned into the pKLV2.2-h7SKgRNA5(SapI)-U6gRNA5(BbsI)-PGKpruo2ABFP-W guide RNA expression vector. Next, U2OS cells were transduced with lentiviral particles harboring the guide expression vector, and 48 h later, the medium was switched to a selection medium (DMEM as described above but containing 4 µg ml^−1^ puromycin). Single clones were expanded in the selection medium in 96-well plates, and PML knockout was validated using western blotting.

The HeLa cell line expressing Flag–mClover3–TDP-43 and HA–mRuby3–PML was generated as follows. Flag–mClover3–TDP-43 and HA–mRuby3–PML were cloned into pCW57.1 vectors harboring a puromycin or blasticidin resistance gene, respectively. Infectious lentiviral particles were generated by transfecting HEK293T cells with these transgene-harboring plasmids together with pPax2 (packaging plasmid) and pMD2.G (VSV-G envelope-expressing plasmid) for 48 h. Supernatants containing lentiviral particles were collected, and HeLa cells were sequentially transduced with these lentiviral particles followed by puromycin (2.5 µg ml^−1^ for 5 days) and blasticidin (10 µg ml^−1^ for 10 days) selection. Surviving clones were expanded in selection medium (DMEM as described above, but containing 2.5 µg ml^−1^ puromycin and 10 µg ml^−1^ blasticidin) followed by fluorescence-activated cell sorting to select cells with homogenous expression levels of the transgenes similar to the expression levels of endogenous TDP-43 and PML.

The generation and cultivation of a HeLa cell line expressing RNF4-3×Flag from the endogenous RNF4 loci was described previously^18^.

### Guide RNA sequences and enzymes used for gene editing

The guide RNA sequences and enzymes used for gene editing of TDP-43 and the generation of PML knockout cells are listed in Supplementary Table 1.

### siRNA sequences

The sequences of all siRNAs used in this work are listed in Supplementary Table 2.

### qPCR primer

The qPCR primers used in this work are listed in Supplementary Table 3.

### Antibodies

All antibodies used for western blotting and immunofluorescence staining are listed in Supplementary Table 4 together with the respective dilutions.

### Solubility assay

HEK293T cells were grown in six-well plates and transfected with the indicated plasmids for 48 h. Before lysis, cells were either left untreated, treated with sodium arsenite (0.5 mM, 1 h) or exposed to heat stress (43 °C, 1 h). Cells were washed twice in cold PBS, followed by scraping in 300 µl modified radioimmunoprecipitation assay (RIPA) buffer on ice (50 mM Tris (pH 8), 150 mM NaCl, 1% NP-40, 5 mM EDTA, 0.5% sodium deoxycholate (SDC), 0.1% SDS, 1 mM phenylmethylsulfonyl fluoride (PMSF), 2 µg ml^−1^ aprotinin, 2 µg ml^−1^ leupeptin, 1 µg ml^−1^ pepstatin A, 10 mM N-ethylmaleimide and 1 tablet PhosStop per 10 ml (Roche)). Lysis was performed by sonication (10 s at 30% of maximum amplitude; Sonics Vibra Cell) followed by rotation at 4 °C for 15 min. In total, 30 µl of the lysates were mixed with Laemmli buffer, boiled (95 °C, 10 min) and kept as input samples and 150 µl of the lysates were fractionated by centrifugation (20,800*g*, 4 °C, 20 min). In total, 130 µl of the supernatant were mixed with Laemmli buffer and boiled (=soluble fraction). The pellet was washed in 1 ml of modified RIPA buffer (20,800*g*, 4 °C, 15 min), and the supernatant was discarded. Pelleted proteins were resolubilized by sonication (10 s at 25% maximum amplitude) in 56.25 µl of resolubilization buffer (7 M urea, 2 M thiourea, 4% Chaps and 30 mM Tris (pH 8.5)). Insoluble material was pelleted by centrifugation (20,800*g*, room temperature, 15 min) and 40 µl of the supernatant was mixed with Laemmli buffer and boiled (=insoluble fraction).

### Identification of TDP-43-interacting proteins by MS

For each replicate, HEK293T cells were grown in 10 cm plates and transfected with either Flag-TDP-43, Flag-SUMO2–TDP-43 or Flag-4×SUMO2–TDP-43 for 72 h. Cells were washed twice in ice-cold PBS and scraped in 1 ml of IP lysis buffer (50 mM HEPES, 150 mM NaCl, 1.5 mM MgCl_2_, 1 mM EGTA, 10% glycerol, 1% Triton, 1 mM PMSF, 2 µg ml^−1^ aprotinin, 2 µg ml^−1^ leupeptin, 1 µg ml^−1^ pepstatin A and 1 tablet PhosStop per 10 ml). Samples were rotated (15 min, 4 °C) and cleared of insoluble material by centrifugation (20,800*g*, 4 °C, 15 min). A Lowry assay was used to determine the protein concentrations in the soluble supernatant fractions. Samples were adjusted to a concentration of 1 mg ml^−1^. For each replicate sample, 20 µl of anti-FLAG M2 magnetic beads (Merck) were washed twice in IP lysis buffer, and 1 ml of lysate was added to the beads.

The samples were rotated for 2 h at 4 °C, and the supernatant was discarded. Beads were first washed three times in IP lysis buffer and then washed three times in wash buffer (50 mM Tris–HCl (pH 7.5)). In total, 5% of the beads were boiled in Laemmli buffer and used for IP controls by western blotting. The remaining beads were mixed with 25 µl of elution buffer (3% SDC and 50 mM Tris (pH 8.5)) and heated to 60 °C for 5 min. To reduce and alkylate the samples, the supernatant was adjusted to 1% SDC, 1 mM tris(2-carboxyethyl)phosphine (TCEP) and 4 mM chloroacetamide and heated to 95 °C for 10 min. Next, 500 ng of trypsin and 500 ng of LysC (diluted in 25 µl of 50 mM Tris–HCl (pH 8.5)) were added, and samples were digested at 37 °C overnight. Digestion was stopped by adding 150 µl of isopropanol/1% trifluoroacetic acid (TFA). Digested samples were purified on STAGE-tips plugged with two styrenedivinylbenzene reveres-phase sultanate (SDB-RPS) disks (total binding capacity 28 µg). Samples were passed through the disks by centrifugation (750*g*, room temperature, 8 min), and the disks were sequentially washed with 200 µl of 1% TFA in isopropanol and 200 µl of 0.2% TFA (750*g*, room temperature, 3 min). Samples were eluted by passing 60 µl of elution buffer (80% acetonitrile and 1.25% NH_4_OH) through each stage tip (3,500*g*, room temperature, 8 min). Eluates were dried by centrifugation in a SpeedVac (30 min, 60 °C). Dried peptides were resuspended in 10 µl of 2% acetonitrile and 0.1% TFA.

Proteomic analyses were performed on an Easy nLC 1200 system (Thermo Fisher Scientific) coupled to Q Exactive HF mass spectrometer (Thermo Fisher Scientific). Peptides were eluted by nonlinear gradient over 75 min. The injection volume was 1 µl.

The mass spectrometer was operated in a data-dependent mode (MS1 scan range = 300–1650 *m*/*z*). Full-scan MS spectra of IP samples were acquired using 3E6 as an AGC target with a resolution of 60,000 at 200 *m*/*z* with a maximum injection time of 20 ms.

The ten most intense ions were fragmented by high collision-induced dissociation. Resolution for MS/MS spectra was set to 30,000 at 200 *m*/*z*, AGC target to 1E5 and maximal injection time was set to 54 ms.

The acquired raw files of mass spectra were analyzed using MaxQuant Software (v.1.6.17.0) and the implemented Andromeda database search engine^64^. Extracted ion spectra were searched against the UniProt human database (v. 2021). The false discovery rate was set to 1%, and the minimal label-free quantification (LFQ) ratio count was set to 2. Oxidation of methionine residues and acetylation of protein N termini were set as variable modifications.

### Ni-NTA pull down

Purification of His-SUMO2 and His-Ub conjugates on magnetic Ni-NTA beads (Qiagen) was performed as described previously^65^. In brief, cells were transfected to express His-SUMO2 alone or cotransfected as indicated. Cells were lysed in Ni-NTA-pull-down lysis buffer (6 M guanidine–HCl, 100 mM NaH_2_PO_4_, 0.05 % Tween 20 and 100 mM Tris–HCl (pH 8.0)), and His-SUMO2 conjugates were purified on Ni-NTA beads which were then and boiled in Laemmli buffer for 10 min.

### Co-IP

Flag-IPs under nondenaturing conditions were performed as follows. HEK293T cells were transfected with plasmids encoding Flag-TDP-43, Flag-SUMO2–TDP-43, Flag-4×SUMO2–TDP-43 or with an empty vector for 48 h. Cells were washed twice in cold PBS and scraped in 1 ml IP lysis buffer per 10 cm dish (50 mM HEPES, 150 mM NaCl, 1,5 mM MgCl_2_, 1 mM EGTA, 10% glycerol, 1% Triton, 1 mM PMSF, 2 µg ml^−1^ aprotinin, 2 µg ml^−1^ leupeptin, 1 µg ml^−1^ pepstatin A and 1 tablet PhosStop per 10 ml (Roche)). Lysates were rotated for 20 min at 4 °C and cleared of insoluble material by centrifugation (20,800*g*, 4 °C, 20 min). In total, 8% of the supernatant was mixed with Laemmli buffer, boiled and kept as an input control. The remaining supernatants were mixed with anti-Flag agarose beads (equilibrated in IP lysis buffer). IPs were rotated for 2–3 h at 4 °C and washed four times in IP lysis buffer. To elute bound proteins, beads were mixed with elution buffer (100 µg ml^−1^ Flag peptide in PBS) and rotated for 30 min at 4 °C. Eluates were mixed with Laemmli buffer and boiled for 10 min.

IPs of endogenous TDP-43 from HEK293T cells were performed as described above, but TDP-43-specific antibodies or rabbit IgG were bound to magnetic beads coated with protein A/G and separated from supernatants in a magnetic rack. TDP-43 and coprecipitated proteins were eluted by boiling the washed beads in Laemmli buffer for 10 min.

### Denaturing co-IP followed by Ni-NTA pull down

For each sample, three 10 cm dishes of HEK293T cells were grown. Cells were transfected with plasmids encoding FRB–PML–Myc and HA–TDP-43–FKBP together with either a plasmid encoding His-Ub or with an empty vector control for 72 h. Before lysis, rapamycin (100 nM, 4 h) or DMSO was added to the growth medium, and cells were treated with heat stress (43 °C, 1 h). Cells were washed twice in cold PBS, and 0.5 ml of denaturing IP lysis buffer was added per 10 cm dish (IP lysis buffer supplemented with 1% SDS). Lysates were sonicated (10 s at 30% of maximum amplitude; Sonics Vibra Cell) and heated to 97 °C for 10 min. Insoluble material was precipitated by centrifugation (20,800*g*, room temperature, 15 min). In total, 40 µl of the lysate was mixed with Laemmli buffer, boiled (97 °C, 10 min) and kept as an input control.

The remaining lysate was diluted with IP lysis buffer to an SDS concentration of 0.1%. In total, 70 µl of magnetic anti-HA-beads (Thermo Fisher Scientific; equilibrated in IP lysis buffer) were added to each lysate, followed by 2 h of rotation at 4 °C. Beads were washed four times in IP lysis buffer, and precipitated proteins were eluted by boiling in Ni-NTA pull-down lysis buffer, followed by the purification of His-ubiquitin conjugates on Ni-NTA beads.

### Immunofluorescence staining and imaging

Cells were grown on glass coverslips. Before fixation, cells were treated as indicated in the figure legends (Figs. 3c and 5d,h and Extended Data Figs. 2c, 3b,c, 4c, 7c,d and 8a). Cells were washed twice in cold PBS and fixed for 15 min in 4% paraformaldehyde in PBS (Morphisto), followed by three washes in PBS. Cells were then permeabilized for 10 min in 0.5% Triton X-100 in PBS, washed three times in PBS and then blocked for 20 min in blocking buffer (2% BSA and 0.1% Tween 20 in PBS). Blocked cells were washed once in PBS before primary antibodies (diluted in blocking buffer) were added at 4 °C overnight. The excess primary antibodies were washed out by three washes in PBS before the secondary antibodies and 1 mg ml^−1^ DAPI were added to the cells for 30 min. If no immunofluorescence staining was performed, fixed cells were incubated for 1 min with 50 µl of NucBlue (Invitrogen) at room temperature. To assay the colocalization of p97 and PML, cells were pre-extracted as described previously^66^. In brief, cells were washed twice with PBS followed by exposure to pre-extraction buffer (25 mM HEPES (pH 7.4), 50 mM NaCl, 3 mM MgCl_2_, 0.5% Triton X-100 and 0.3 M sucrose) for 10 min. Stained cells were washed three times in PBS and once in ddH_2_O before the coverslips were mounted face down on microscope slides using Pro-Long gold antifade reagent (Invitrogen). Images were taken with a Leica TCS SP8 confocal microscope using Leica LAS X software (v.2.0.2.15022).

### Quantification and statistical analysis of immunofluorescence microscopy data

Raw images were converted to grayscale images using the Fiji-BioVoxxel bundle in ImageJ (v.1.52i) followed by analysis with CellProfiler software (v.4.2.6). Only transfected cells were analyzed. Cells without detectable nuclear PML or Sp100 foci or cytoplasmic SGs were excluded from the analysis. To determine NB-to-nucleus ratios (of HA–TDP-43–FKBP, mClover3–TDP-43 or endogenous TDP-43), the respective signal was measured within detected NBs (foci of FRB–PML–Myc, FRB–Sp100–Myc, mRuby3–PML or endogenous PML) and the nucleus of the same cell. The ratio of both measurements was calculated per cell. The nuclear area was determined based on the DAPI signal. The Flag-TDP-43-to-PML and p97-to-PML signal ratios were determined within the boundaries of detected nuclear PML foci. Mean signal intensities of Flag-TDP-43, p97 and PML were used for these calculations. The Flag-TDP-43-to-PML signal ratio was normalized to the Flag-TDP-43 expression levels in each cell to avoid the introduction of biases by uneven expression levels. Signal correlation analysis was performed within the nuclear area of transfected cells. For all measurements, one data point per cell was generated. Enrichment of TDP-43–GFP within SGs was performed within the area of SGs as determined based on the G3BP2 signal. Generation of violin plots or graphs and calculation of *P* values of two-tailed, unpaired Student’s *t* tests were performed with GraphPad Prism software (v.9.5.1).

### Quantification and statistical analysis of Western blot data

Blots and gels of the same experiment were always processed in parallel. For the western blots shown in Fig. 1b,c (and for the respective replicates analyzed in Extended Data Fig. 1a), chemiluminescence detection on film was used. These western blots were quantified using ImageJ2 software (v.2.14.0). For all other blots, the LI-COR Biosciences ODYSSEY DLx system was used to detect signals, and LI-COR Biosciences Image Studio software (v.5.2.5) was used for signal quantification.

The insoluble fraction of SUMO–TDP-43 and FKBP–TDP-43 fusion proteins was calculated as follows. For each treatment condition, the band intensity of the insoluble fraction was divided by the sum of the respective band intensities in the soluble and insoluble fractions.

The rescue of HA–TDP-43–FKBP solubility by rapamycin treatment was calculated by analyzing the percentage of insoluble HA–TDP-43–FKBP in DMSO and rapamycin-treated samples (for example, if the insoluble fraction of TDP-43 in heat-stressed and DMSO-treated cells is 70% and the insoluble fraction in heat-stressed and rapamycin-treated cells is 28%, the rescue value would be 100 − 28/70 = 60%).

When cells were additionally treated with the inhibitors MG132, DBeQ or TAK-243 (as analyzed in Extended Data Fig. 6b,c and Extended Data Fig. 7a), the relative rescue by rapamycin treatment between control cells (treated with heat stress or sodium arsenite and rapamycin) and inhibitor-treated cells was calculated by setting the mean rescue in control cells to 100%.

Three to five independent replicates were analyzed for each of the experiments. Generation of bar graphs and calculation of *P* values of Student’s *t* tests were performed with GraphPad Prism software (v.9.5.1).

### Statistical analysis of MS data

Perseus 1.6.15.0 software^67^ was used for statistical analysis of MS data. Volcano plots were created in RStudio and displayed partially imputed data. Missing LFQ intensities for a given protein in the 4×SUMO2–TDP-43 versus TDP-43 WT volcano plot were imputed if intensity values were measured in all three replicates of 4×SUMO2–TDP-43 IPs. Missing LFQ intensities for a given protein in the SUMO2–TDP-43 versus TDP-43 WT volcano plot were imputed if intensity values were measured in all three replicates of TDP-43 IPs.

For GO cellular component (GOCC) and GO biological process enrichment analysis, gene lists were created as follows. Gene names of significantly enriched (>2-fold enrichment with a *P* < 0.05) and exclusively detected proteins (in all three replicates of A, in A versus B) were combined in the enrichment list, and the full list of all proteins detected was used as a background list. The database for annotation, visualization and integrated discovery (DAVID, version December 2021, knowledgebase version v2023q4) and R studio (v.4.1.2) were used for GO term enrichment analysis using the aforementioned gene lists. The list of proteins significantly enriched in IPs from Flag-4×SUMO2–TDP-43 expressing cells (versus IPs from Flag-TDP-43 expressing cells) and proteins exclusively detected in all three IPs from Flag-4×SUMO2–TDP-43 expressing cells was used for the generation of the STRING network using the STRING database (v.11.5, https://string-db.org) and Cytoscape (v.3.9.1). Only the highest confidence interactions (interaction score >0.9) were considered. Experiments, databases and text mining were used as interaction sources. Proteins associated with the GOCC term ‘PML body’ were filtered and are displayed. Disconnected nodes are hidden.

### Statistics and reproducibility

Four (sodium arsenite treatment and associated controls) or five (heat stress and associated controls) independent replicates were used for the statistical analysis of solubility assays shown in Extended Data Fig. 6c (associated with Fig. 5c). Three independent replicate experiments were used for the statistical analysis of all other solubility assays shown in Extended Data Figs. 1a,c,d, 6a–e, 7a and 8c (associated with Figs. 1b–e, 5a–c and 5f,g,i). If western blots were not analyzed statistically, a representative example of three independent experiments is shown. All microscopy experiments were performed in three independent replicates, and representative images are displayed. If microscopy data were analyzed statistically, a representative replicate was used for the measurements described above. MS was performed with three replicate experiments. qPCR was performed in three independent replicates, and four measurements were performed per replicate.

### Preparation of model figures

The graphical abstract, Figs. 3a and 6 and elements of Fig. 1a and Extended Data Fig. 4b were created with BioRender.com.

### Reporting summary

Further information on research design is available in the Nature Portfolio Reporting Summary linked to this article.

## Online content

Any methods, additional references, Nature Portfolio reporting summaries, source data, extended data, supplementary information, acknowledgements, peer review information; details of author contributions and competing interests; and statements of data and code availability are available at 10.1038/s41589-025-01886-4.

## Supplementary information

Supplementary Information Supplementary Tables 1–4.

Reporting Summary

Supplementary Data Summary of MS data shown in Fig. 2 and Extended Data Fig. 2.

## Source data

Source Data Figs. 1–5 and Extended Data Figs. 1, 4 and 5 Western blot (uncropped, unedited blots) images for Figs. 1b–e, 2d–g, 3b,d–g, 4a–g and 5a–c,e–g,i and Extended Data Figs. 1b, 4a,b and 5a–d.

Source Data Fig. 5 and Extended Data Figs. 1–3 and 6–8 Statistical source data for Fig. 5d and Extended Data Figs. 1a,c,d, 2d,e, 3a, 6a–e, 7a–d and 8b,c.

## Data Availability

The MS data generated in this study are deposited in the proteomics identification database (PRIDE) database (https://www.ebi.ac.uk/pride) and will be made public before the publication of this manuscript. Project name: Interactome of Flag-TDP-43, Flag-SUMO2–TDP-43 and Flag-4×SUMO2–TDP-43; project accession PXD050322. Reviewer account details—username: reviewer_pxd050322@ebi.ac.uk; password: NJN2H6j6. Raw data of Figs. 1–6 and Extended Data Figs. 1–8 are supplied in the source data files. Figures and figure parts created with BioRender were made publicly available under a publication license and can be accessed via the following links: https://BioRender.com/x96y005 (graphical abstract and Fig. 6), https://BioRender.com/q31l104 (left side of Fig. 1a), https://BioRender.com/t36c052 (right side of Fig. 1a), https://BioRender.com/g84q656 (Fig. 3a) and https://BioRender.com/y86a395 (Extended Data Fig. 4b) Source data are provided with this paper.
