# Supplementary material for: Induced proximity to PML protects TDP-43 from aggregation via SUMO–ubiquitin networks
Source: Nat Chem Biol. 2025 Apr 17;21(9):1408–19. doi: 10.1038/s41589-025-01886-4 (PMC12394070; doi:10.1038/s41589-025-01886-4)
Supplement: Supplementary file 1 — Supplementary Tables 1–4. [file 41589_2025_1886_MOESM1_ESM.pdf]

nature chemical biology

Supplementary information

https://doi.org/10.1038/s41589-025-01886-4

Induced proximity to PML protects TDP-43 from aggregation via SUMO–ubiquitin networks

In the format provided by the authors and unedited

Supplementary Table 1: Sequences of guideRNAs

guideRNA	Sequence
TDP-43<br>(exon 6, LbCas12a crRNA, direct repeat and spacer)	AAGUGUAGAUGCUGGGGAAUGUAGACAGUG
PML<br>(exon 3, SpyoCas9 sgRNA)	GCGGTACCAGCGCGACTACG
PML<br>(exon 5, SpyoCas9 sgRNA)	AGAAGGCGTACACTG GCACG

Supplementary Table 2: Sequences of siRNAs

siRNA	Sequence
siControl	CGUAGCCGGAAUACUUCG A(TT)
siPIAS1	CCGGAUCAUUCUAGAGCUU(TT)
siPML	GGGGAAAGAUGCAGCUGUA(TT)
siRNF4	GGGCAUGAAAGGUUGAGAA(TT)
SiTopors-1	CAAGGAGCCUGUCUAGUAA (TT)
SiTopors-2	GUCCUAAGGCCUUCGUAUAAU(TT)

Supplementary Table 3: Sequences of qPCR Primers

Primer	Sequence
GAPDH for	TCGGAGTCAACGGATTTG
GAPDH rev	CAACAATATCCACTTTACCCAGAG
TOPORS for	ATCCTCCGAGATAATGGC
TOPORS rev	AGCTGGTACTGTCTGTTG

Supplementary Table 4: Antibodies used in this publication

Antibody	Supplier	Product number	RRID	Host species	Dilution
Anti-β-Tubulin	Developmental Studies Hybridoma Bank	clone E7	AB_2315513	Mouse	WB: 1:3000
Anti-DAXX	Cell Signaling Technology	4533	AB_2088778	Rabbit	WB: 1:1000
Anti-FLAG M2	Sigma Aldrich	F1804	AB_262044	Mouse	WB: 1:1000<br>IF: 1:1000
Anti-G3BP2	Cell Signalling Technology	31799	AB_2920540	Rabbit	IF: 1:250
Anti-His	Santa Cruz Biotechnology	sc-53073	AB_783791	Mouse	WB: 1:1000
Anti-HA	Novus Biologicals	NB600-362	AB_10124937	Goat	IF: 1:800
Anti-HA	Abcam	ab9110	AB_307019	Rabbit	WB: 1:1000<br>IF: 1:250
Anti-Myc	Cell Signaling Technology	2276	AB_331783	Mouse	WB: 1:5000
Anti-Myc	Cell Signaling Technology	2272	AB_10692100	Rabbit	IF: 1:400
Anti-PIAS1	Cell Signaling Technology	3550	AB_1904090	Rabbit	WB: 1:1000<br>IF: 1:200
Anti-PML	Santa Cruz Biotechnology	sc-5621	AB_2166848	Rabbit	IF: 1:200
Anti-PML	Abcam	ab179466	AB_2891128	Rabbit	WB: 1:2000
Anti-RGS-His	Qiagen	34650	AB_2687898	Mouse	WB: 1:1000
Anti-RNF4	Proteintech	17810-1-AP	AB_2878443	Rabbit	WB: 1:1000
Anti-TDP-43	EnCor Biotechnology	MCA-3H8	AB_2572387	Mouse	WB 1:5000<br>IF: 1:1000
Anti-SP100	Abcam	ab167605	Not listed	Mouse	IF: 1:300
Anti-VCP/p97	Thermo Fisher Scientific	MA3-004	AB_221463	Mouse	IF: 1:200
IRDye® 680RD anti-Mouse IgG Secondary Antibody	Li-Cor	926-68070	AB_10956588	Goat	WB: 1:10000
IRDye® 680RD anti-Rabbit IgG Secondary Antibody	Li-Cor	926-68071	AB_10956166	Goat	WB: 1:10000
IRDye® 800CW anti-Mouse IgG Secondary Antibody	Li-Cor	926-32210	AB_621842	Goat	WB: 1:10000
IRDye® 800CW anti-Rabbit IgG Secondary Antibody	Li-Cor	926-32211	AB_621843	Goat	WB: 1:10000
Alexa Fluor® 488 Cross-Absorbed anti-goat IgG (H+L)	Thermo Fisher Scientific	A11055	AB_2534102	Donkey	IF: 1:1000
Cy3-AffiniPure Anti-Mouse IgG (H+L)	Jackson ImmunoResearch Labs	715-165-150	AB_2340813	Donkey	IF: 1:1000
Cy5-AffiniPure Anti-Rabbit IgG (H+L)	Jackson ImmunoResearch Labs	711-175-152	AB_2340607	Donkey	IF: 1:1000
