# Supplementary material for: Induced proximity to PML protects TDP-43 from aggregation via SUMO–ubiquitin networks
Source: Nat Chem Biol. 2025 Apr 17;21(9):1408–19. doi: 10.1038/s41589-025-01886-4 (PMC12394070; doi:10.1038/s41589-025-01886-4)
Supplement: Supplementary file 2 — Reporting Summary [file 41589_2025_1886_MOESM2_ESM.pdf]

nature portfolio

Corresponding author(s): Stefan Muller and Jan Keiten-Schmitz

Last updated by author(s): Feb 28, 2025

# Reporting Summary

Nature Portfolio wishes to improve the reproducibility of the work that we publish. This form provides structure for consistency and transparency in reporting. For further information on Nature Portfolio policies, see our Editorial Policies and the Editorial Policy Checklist.

## Statistics

For all statistical analyses, confirm that the following items are present in the figure legend, table legend, main text, or Methods section.

| n/a | Confirmed | |
|---|---|---|
| ☐ | ☒ | The exact sample size (*n*) for each experimental group/condition, given as a discrete number and unit of measurement |
| ☐ | ☒ | A statement on whether measurements were taken from distinct samples or whether the same sample was measured repeatedly |
| ☐ | ☒ | The statistical test(s) used AND whether they are one- or two-sided<br>*Only common tests should be described solely by name; describe more complex techniques in the Methods section.* |
| ☐ | ☒ | A description of all covariates tested |
| ☐ | ☒ | A description of any assumptions or corrections, such as tests of normality and adjustment for multiple comparisons |
| ☐ | ☒ | A full description of the statistical parameters including central tendency (e.g. means) or other basic estimates (e.g. regression coefficient) AND variation (e.g. standard deviation) or associated estimates of uncertainty (e.g. confidence intervals) |
| ☐ | ☒ | For null hypothesis testing, the test statistic (e.g. *F*, *t*, *r*) with confidence intervals, effect sizes, degrees of freedom and *P* value noted<br>*Give P values as exact values whenever suitable.* |
| ☒ | ☐ | For Bayesian analysis, information on the choice of priors and Markov chain Monte Carlo settings |
| ☒ | ☐ | For hierarchical and complex designs, identification of the appropriate level for tests and full reporting of outcomes |
| ☒ | ☐ | Estimates of effect sizes (e.g. Cohen's *d*, Pearson's *r*), indicating how they were calculated |

*Our web collection on statistics for biologists contains articles on many of the points above.*

## Software and code

Policy information about availability of computer code

Data collection

Western Blot Data: Licor Image Studio Lite (version 5.2.5)

Microscopy data: Leica LAS X (version 2.0.2.15022)

Mass spectrometry raw data processing: MaxQuant (version 1.6.17.0)

Data analysis

Licor Image Studio Lite (version 5.2.5) and ImageJ2 (version 2.14.0) were used for quantification of Western Blots.

Fiji-BioVoxxel bundle in ImageJ (version 1.52i) and CellProfiler (version 4.2.6) were used for preparation of IF figures and IF data analysis, respectively.

For MS data analysis and data presentation/figure creation the following programms were used:
MaxQuant (version 1.6.17.0 and the implemented Andromeda database search engine, in combination with the Uniprot human database (version 2021), Perseus (version 1.6.15.0) , R studio (version 4.1.2), STRING database (version 11.5, https://string-db.org), Cytoscape (version 3.9.1) and the database for annotation visualization and integrated discovery (DAVID, version Dec. 2021, knowledgebase version v2023q4)

GraphPad PRISM (version 9.5.1) was used for statistical analysis of Western Blot and immunofluorescence data and creation figures presenting the statistical anlysis of these data.

STRING database (version 11.5)

Models in the figures and the graphical abstract were created using the BioRender online tool (biorender.com).

For manuscripts utilizing custom algorithms or software that are central to the research but not yet described in published literature, software must be made available to editors and reviewers. We strongly encourage code deposition in a community repository (e.g. GitHub). See the Nature Portfolio guidelines for submitting code & software for further information.

## Data

Policy information about availability of data

All manuscripts must include a data availability statement. This statement should provide the following information, where applicable:

- Accession codes, unique identifiers, or web links for publicly available datasets
- A description of any restrictions on data availability
- For clinical datasets or third party data, please ensure that the statement adheres to our policy

MS data are uploaded on PRIDE and will be publicly available following publication.

PRIDE dataset identifier: PXD050322
Username: reviewer_pxd050322@ebi.ac.uk
Password: NJN2H6j6
Unprocessed and uncropped Western Blots and statistical raw data are supplied in the Source Data files.

## Human research participants

Policy information about studies involving human research participants and Sex and Gender in Research.

| | |
|---|---|
| Reporting on sex and gender | N/A |
| Population characteristics | N/A |
| Recruitment | N/A |
| Ethics oversight | N/A |

Note that full information on the approval of the study protocol must also be provided in the manuscript.

# Field-specific reporting

Please select the one below that is the best fit for your research. If you are not sure, read the appropriate sections before making your selection.

☒ Life sciences ☐ Behavioural & social sciences ☐ Ecological, evolutionary & environmental sciences

For a reference copy of the document with all sections, see nature.com/documents/nr-reporting-summary-flat.pdf

# Life sciences study design

All studies must disclose on these points even when the disclosure is negative.

| | |
|---|---|
| Sample size | Sample sizes were chosen to ensure proper statsitical analysis as explained in the material & methods section and figure legends.<br>Statistical Analysis of western Blot data was performed using all of the three to five independent replicates of the respective experiments. |
| Data exclusions | Cells that were cut of at the edge of the image were excluded from image analysis. |
| Replication | All experiments (Western blots, IPs, immunofluorescence, qPCR) were perfomed as sufficient independent biological replicates to ensure correctness of the data. The number of independent replicates is listed in the respective figure legends.<br>Statistical analysis of Western blots is based on three to five independent replicates.<br>In order to avoid biases associated with adjustments of the microscopy analysis pipeline (in order to adjust for differences in cell density, fixation, staining etc.) an experimental replicate which represented the observed effects of all independent replicates was chosen and analyzed. Large scale experiments as mass spectrometry and automated image analysis were performed with three replicates.<br>qPCR experiments were performed in three independent biological replicates with four technical replicates per biological replicate.<br><br>All replicates confirmed the observations stated in the manuscript. |
| Randomization | In all cell biological experiments cells were randomised to wells, and then wells randomized to treatments. Areas for microscopic imaging followed by statistical analysis were chosen radomly. |

| Blinding | Initial microscopic image analysis was performed in a blinded manner. Furthermore, software was used for statistical calculations and to analyse MS data and IF images. The same analysis parameters were applied to all samples which rules out analytical biases introduced by the experimentors. |
|---|---|

# Reporting for specific materials, systems and methods

We require information from authors about some types of materials, experimental systems and methods used in many studies. Here, indicate whether each material, system or method listed is relevant to your study. If you are not sure if a list item applies to your research, read the appropriate section before selecting a response.

## Materials & experimental systems

| n/a | Involved in the study |
|---|---|
| ☐ | ☒ Antibodies |
| ☐ | ☒ Eukaryotic cell lines |
| ☒ | ☐ Palaeontology and archaeology |
| ☒ | ☐ Animals and other organisms |
| ☒ | ☐ Clinical data |
| ☒ | ☐ Dual use research of concern |

## Methods

| n/a | Involved in the study |
|---|---|
| ☒ | ☐ ChIP-seq |
| ☒ | ☐ Flow cytometry |
| ☒ | ☐ MRI-based neuroimaging |

## Antibodies

Antibodies used

Anti-β-Tubulin, Developmental Studies Hybridoma Bank, clone E7, RRID:AB_2315513, WB: 1:3000

Anti-FLAG M2, Sigma Aldrich, Cat# F1804, RRID: AB_262044, WB: 1:1000, IF: 1:1000

Anti-RGS-His, Qiagen ,Cat# 34650, RRID:AB_2687898, WB: 1:1000

Anti-His, Santa Cruz Biotechnology, Cat# sc-53073, RRID:AB_783791, WB: 1:1000

Anti-HA, Abcam, Cat# ab9110, RRID:AB_307019, WB: 1:1000, IF: 1:250

Anti G3BP2, Cell Signalling Technology, Cat# 31799, RRID:AB_2920540, IF: 1:250

Anti-Myc, Cell Signaling Technology, Cat# 2276, RRID:AB_331783, WB: 1:5000

Anti-Myc, Cell Signaling Technology Cat# 2272, RRID:AB_10692100, IF: 1:400

Anti-TDP-43, EnCor Biotechnology Cat# MCA-3H8, RRID:AB_2572387, WB 1:5000, IF: 1:1000

Anti PML, Abcam, Cat# ab179466, RRID:AB_2891128, WB: 1:2000

Anti-SP100, Abcam, ab167605, No RRID, IF: 1:300

Anti-DAXX, Cell Signaling Technology, Cat# 4533, RRID:AB_2088778, WB: 1:1000

Anti-RNF4, Proteintech, Cat# 17810-1-AP, RRID:AB_2878443, WB: 1:1000

Anti-PIAS1, Cell Signaling Technology, Cat# 3550, RRID:AB_1904090, WB: 1:1000, IF: 1:200

Anti-VCP/p97, Thermo Fisher Scientific, Cat# MA3-004, RRID:AB_221463, IF: 1:200

IRDye® 800CW Goat anti-Mouse IgG Secondary Antibody, Li-Cor, 926-32210, RRID: AB_621842, WB: 1:10000

IRDye® 800CW Goat anti-Rabbit IgG Secondary Antibody, Li-Cor, 926-32211, RRID: AB_621843, WB: 1:10000

Alexa Fluor® 488 Cross-Absorbed donkey anti-goat IgG (H+L), Thermo Fisher Scientific, Cat# A11055, RRID:AB_2534102, IF: 1:1000

Cy3-AffiniPure Donkey Anti-Mouse IgG (H+L), Jackson ImmunoResearch Labs Cat# 715-165-150, RRID:AB_2340813, IF: 1:1000

Cy5-AffiniPure Donkey Anti-Rabbit IgG (H+L) Jackson ImmunoResearch Labs Cat# 711-175-152, RRID:AB_2340607, IF: 1:1000

Anti-HA, Novus Biologicals, Cat# NB600-362, RRID: AB_10124937, IF 1:800

Anti-PML, Santa Cruz Biotechnology, Cat# sc-5621, RRID: AB_2166848, IF: 1:200

IRDye® 680RD anti-Mouse IgG Secondary Antibody, Li-Cor, Cat#926-68070, RRID: AB_10956588, WB: 1:10000

IRDye® 680RD anti-Rabbit IgG Secondary Antibody, Li-Cor, Cat#926-68071, RRID: AB_10956166, WB: 1:10000

Validation

Anti-β-Tubulin, Developmental Studies Hybridoma Bank, clone E7, RRID:AB_2315513: validation PMID: 32200800 PMID: 31833223 PMID: 31328806

Anti-FLAG M2, Sigma Aldrich,Cat# F1804, RRID: AB_262044: validation PMID: 31399583 PMID: 31900387 PMID: 32034124

Anti-RGS-His, Qiagen, 34610: validation PMID: 11591650

Anti-His, Santa Cruz Biotechnology, Cat# sc-53073, RRID:AB_783791: validation PMID: 35427781

Anti-HA, Abcam, Cat# ab9110, RRID:AB_307019: validation on manufacturer's website

Anti G3BP2, Cell Signalling Technology, Cat# 31799, RRID:AB_2920540: validation PMID: 37983241

Anti-Myc, Cell Signaling Technology, Cat# 2276, RRID:AB_331783 (Western blots): validation on manufacturers' website

Anti-Myc, Cell Signaling Technology Cat# 2272, RRID:AB_10692100, (immunofluorescence): validation on manufacturer's website

Anti-TDP-43, EnCor Biotechnology Cat# MCA-3H8, RRID:AB_2572387: validation PMID: 17023659

Alexa Fluor® 488 Cross-Absorbed donkey anti-goat IgG (H+L), Thermo Fisher Scientific, Cat# A11055, RRID:AB_2534102 validation on manufacturers website

Alexa Fluor® 488 Cross-Absorbed donkey anti-goat IgG (H+L), Thermo Fisher Scientific, Cat# A11055, RRID:AB_2534102

Anti PML, Abcam, Cat# ab179466, RRID:AB_2891128: validation on manufacturer's website

Anti-SP100, Abcam, ab167605:  validation on manufacturer's website

Anti-DAXX, Cell Signaling Technology, Cat# 4533, RRID:AB_2088778 validation PMID: 34408321

Anti-RNF4, Proteintech, Cat# 17810-1-AP, RRID:AB_2878443: validation PMID: 36587767

Anti-PIAS1, Cell Signaling Technology, Cat# 3550, RRID:AB_1904090: validation PMID: 26257066

Anti-VCP/p97, Thermo Fisher Scientific, Cat# MA3-004, RRID:AB_221463: validation PMID: 33966597

IRDye® 800CW Goat anti-Mouse IgG Secondary Antibody, Li-Cor, 926-32210, RRID: AB_621842: validation on manufacturer's website

IRDye® 800CW Goat anti-Rabbit IgG Secondary Antibody, Li-Cor, 926-32211, RRID: AB_621843: validation on manufacturer's website

Alexa Fluor® 488 Cross-Absorbed donkey anti-goat IgG (H+L), Thermo Fisher Scientific, Cat# A11055, RRID:AB_2534102: validation PMID: 39933923

Cy3-AffiniPure Donkey Anti-Mouse IgG (H+L), Jackson ImmunoResearch Labs Cat# 715-165-150, RRID:AB_2340813: validation: PMID: 32521226

Cy5-AffiniPure Donkey Anti-Rabbit IgG (H+L) Jackson ImmunoResearch Labs Cat# 711-175-152, RRID:AB_2340607: validation: PMID: 32521226

Anti-HA, Novus Biologicals, Cat# NB600-362, RRID: AB_10124937: validation on manufacturer's website

Anti-PML, Santa Cruz Biotechnology, Cat# sc-5621, RRID: AB_2166848: validation: PMID: 26566030

IRDye® 680RD anti-Mouse IgG Secondary Antibody, Li-Cor, Cat#926-68070, RRID: AB_10956588: validation on manufacturer's website

IRDye® 680RD anti-Rabbit IgG Secondary Antibody, Li-Cor, Cat#926-68071, RRID: AB_10956166: validation on manufacturer's website

## Eukaryotic cell lines

Policy information about cell lines and Sex and Gender in Research

Cell line source(s)

HeLa (human, female, ATCC, CCL-2)

HeLa RNF4-3xFlag (human, female, 3xFlag-Tag was added to C-terminus of endog. RNF4 in the HeLa cell line listed above using CRISPR/Cas9)

HeLa TDP-43-GFP (HeLa cells expressing TDP-43-GFP from a BAC transgene, kindly provided by Simon Alberti, PMID: 28377462)

HeLa TDP-43-FKBP-HA/FRB-PML-Myc was created by genome editng of the endogenous TDP-43 gene locus in the HeLa cells mentioned above. A FRB-PML-Myc expression cassette was integrated by infection with lentiviral particles. For details see methods section of the associated manuscript.)

HEK293T (human,female, ATCC, CRL-3216)

U-2 OS (human, female, ATCC HTB-96)

U-2 OS PML knockout (PML was knocked out in the cell line mentioned above using the CRISPR/Cas system.)

Authentication

HeLa and HEK293T and U 2-OS cells were authenticated by STR profiling.

HeLa RNF4-3xFlag was created by genome editing into authenticated HeLa cells.

HeLa TDP-43-GFP was created by integration of a BAC transgene into authenticated HeLa cells.

HeLa TDP-43-FKBP-HA/FRB-PML-Myc was generated by genome engineering using the CRISPR Cas system and lentiviral transduction in authenticated HeLa cells.

U 2-OS PML knockout cells were generated in authenticated U 2-OS cells.

Mycoplasma contamination

All cell lines were tested negative for mycoplasma contamination.

Commonly misidentified lines
(See ICLAC register)

No commonly misidentified cell line was used in this study
