# Supplementary material for: Induced proximity to PML protects TDP-43 from aggregation via SUMO–ubiquitin networks
Source: Nat Chem Biol. 2025 Apr 17;21(9):1408–19. doi: 10.1038/s41589-025-01886-4 (PMC12394070; doi:10.1038/s41589-025-01886-4)

1b

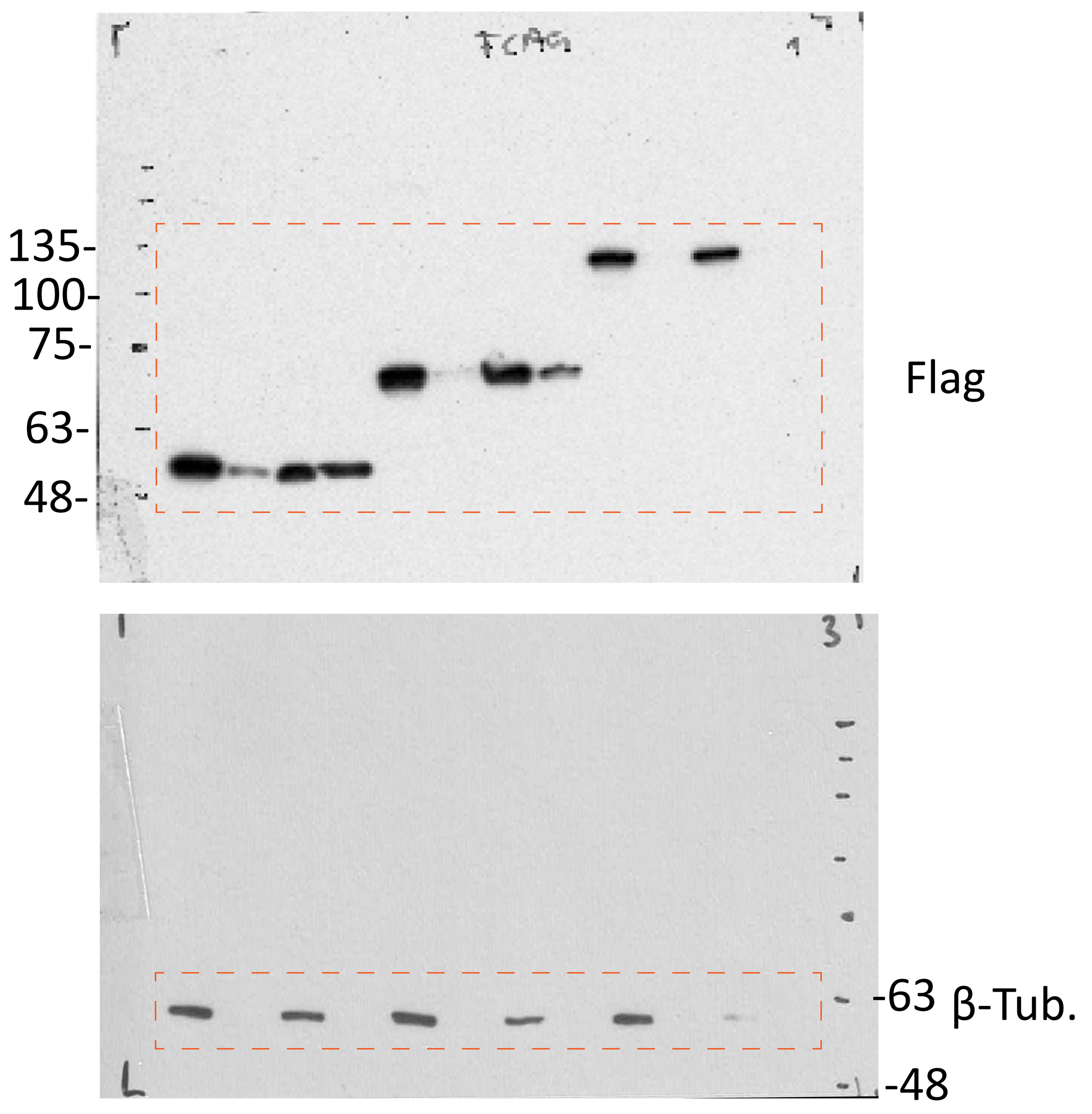
135-
100-
75-
63-
48-
Flag
-63 β-Tub.
-48

1c

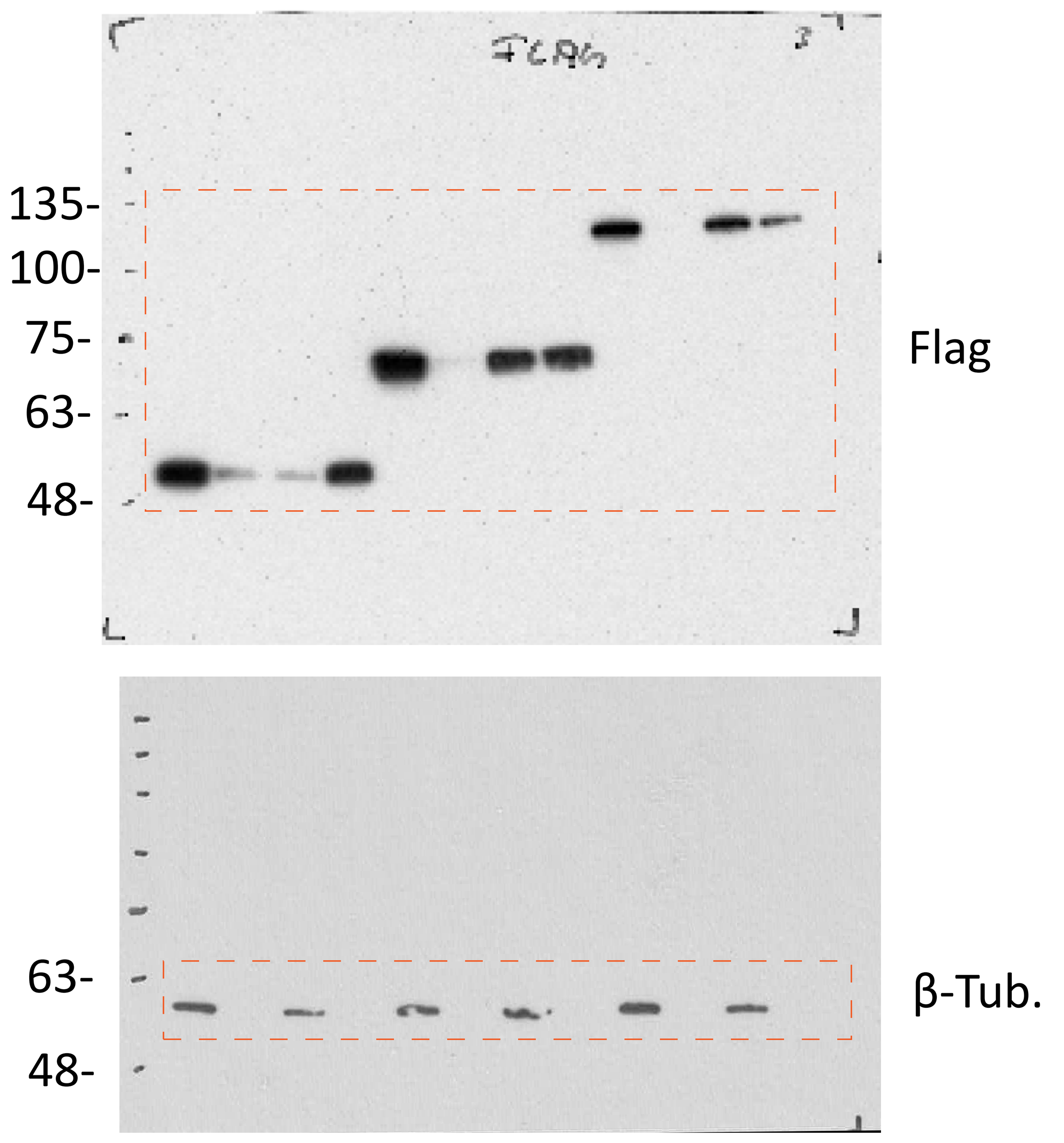
135-
100-
75-
63-
48-
Flag
63-
48-
β-Tub.

1d

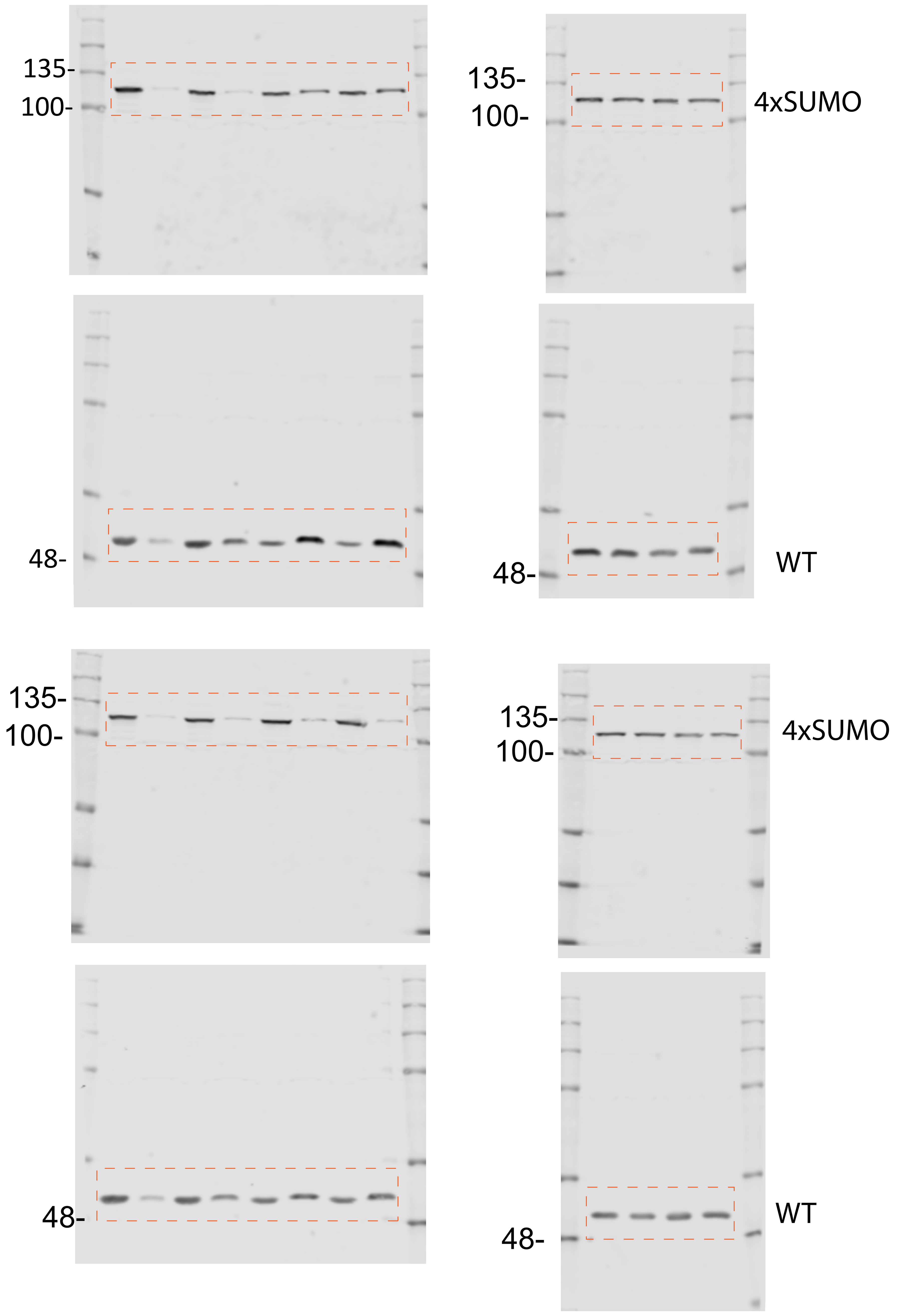
135-
100-
4xSUMO
48-
WT
135-
100-
4xSUMO
48-
WT

1e

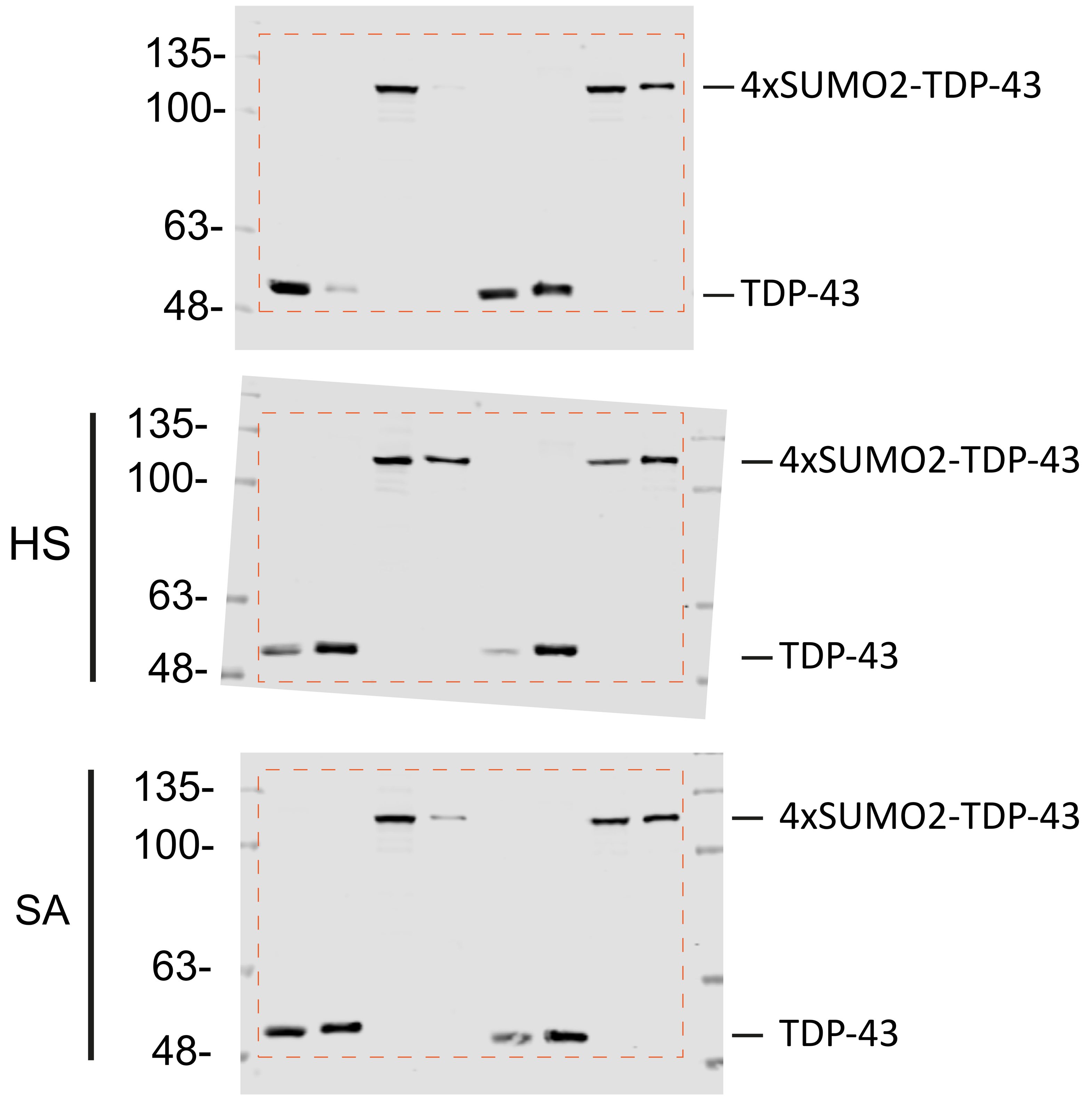
135-
100-
63-
48-
4xSUMO2-TDP-43
TDP-43
HS
135-
100-
63-
48-
4xSUMO2-TDP-43
TDP-43
SA
135-
100-
63-
48-
4xSUMO2-TDP-43
TDP-43

2d

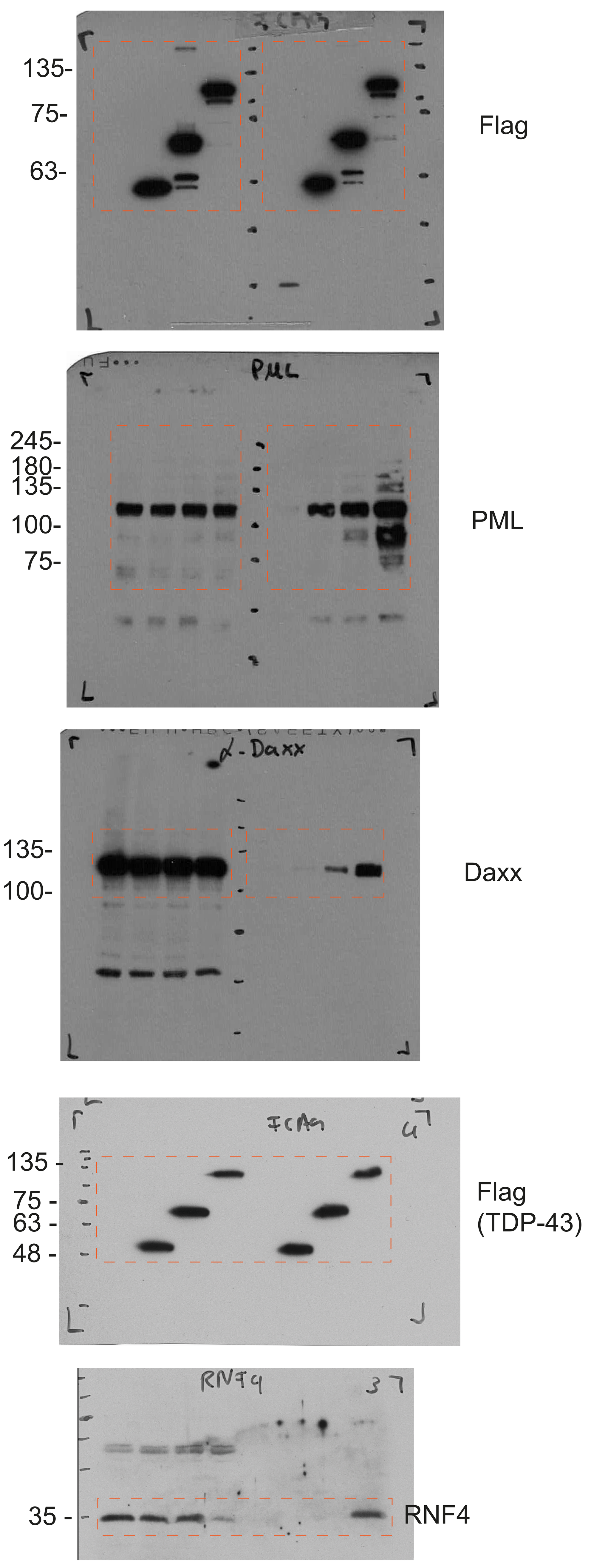
135-
75-
63-
Flag
245-
180-
135-
100-
75-
PML
135-
100-
Daxx
135
75
63
48
Flag
(TDP-43)
35
RNF4

2e

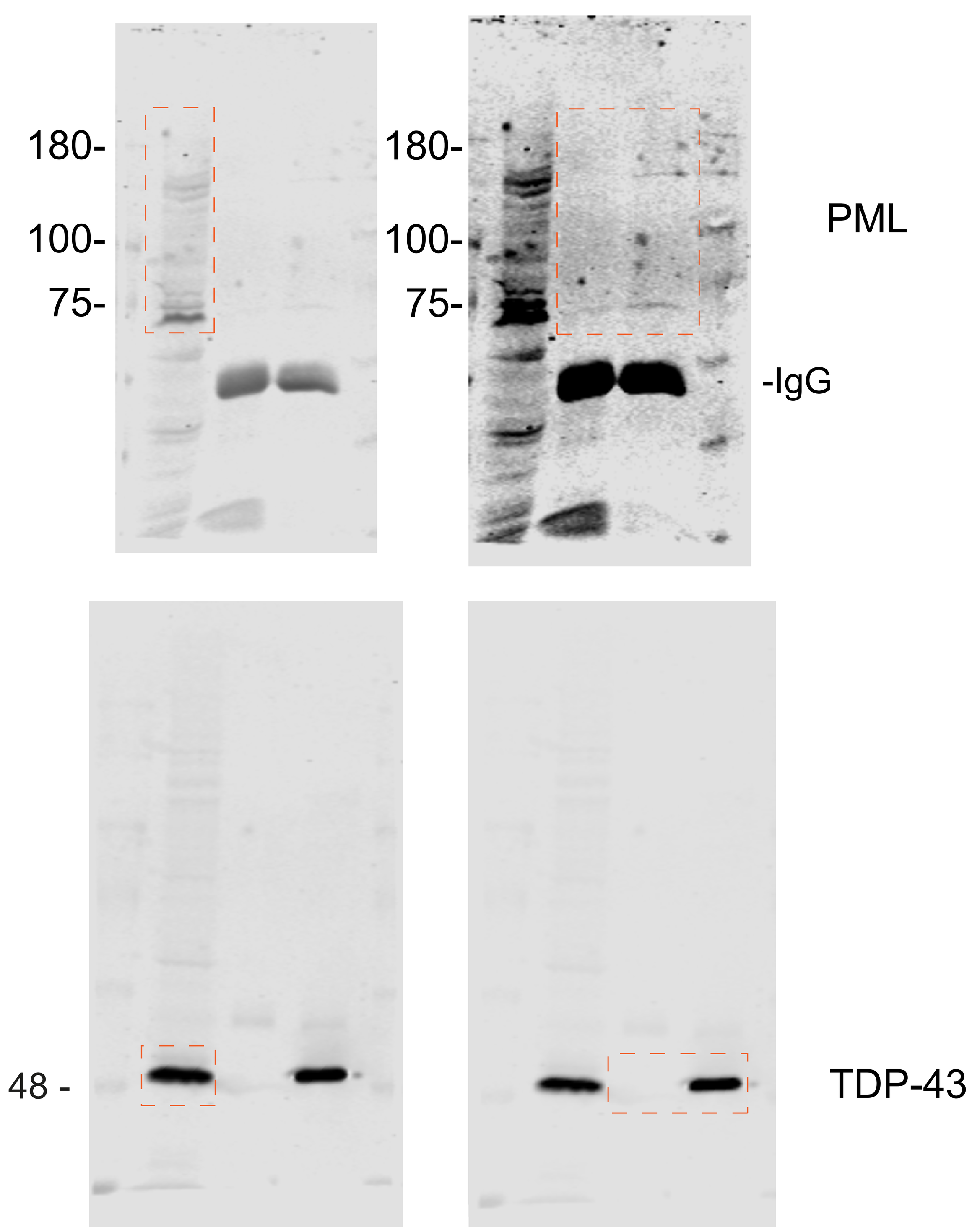
180-
100-
75-
PML
-IgG
48 -
TDP-43

2f

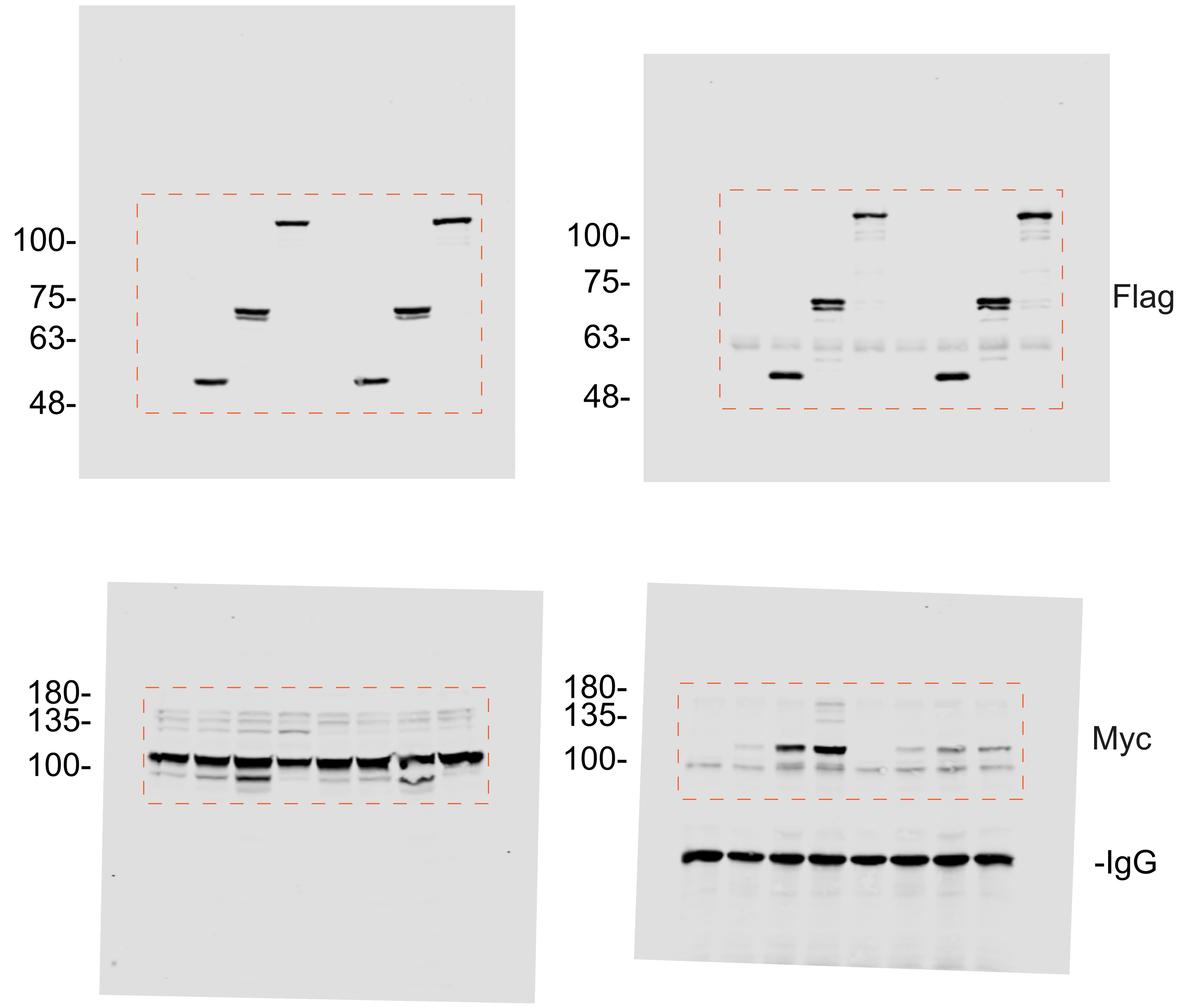
100-
75-
63-
48-
Flag
180-
135-
100-
Myc
-IgG

2g

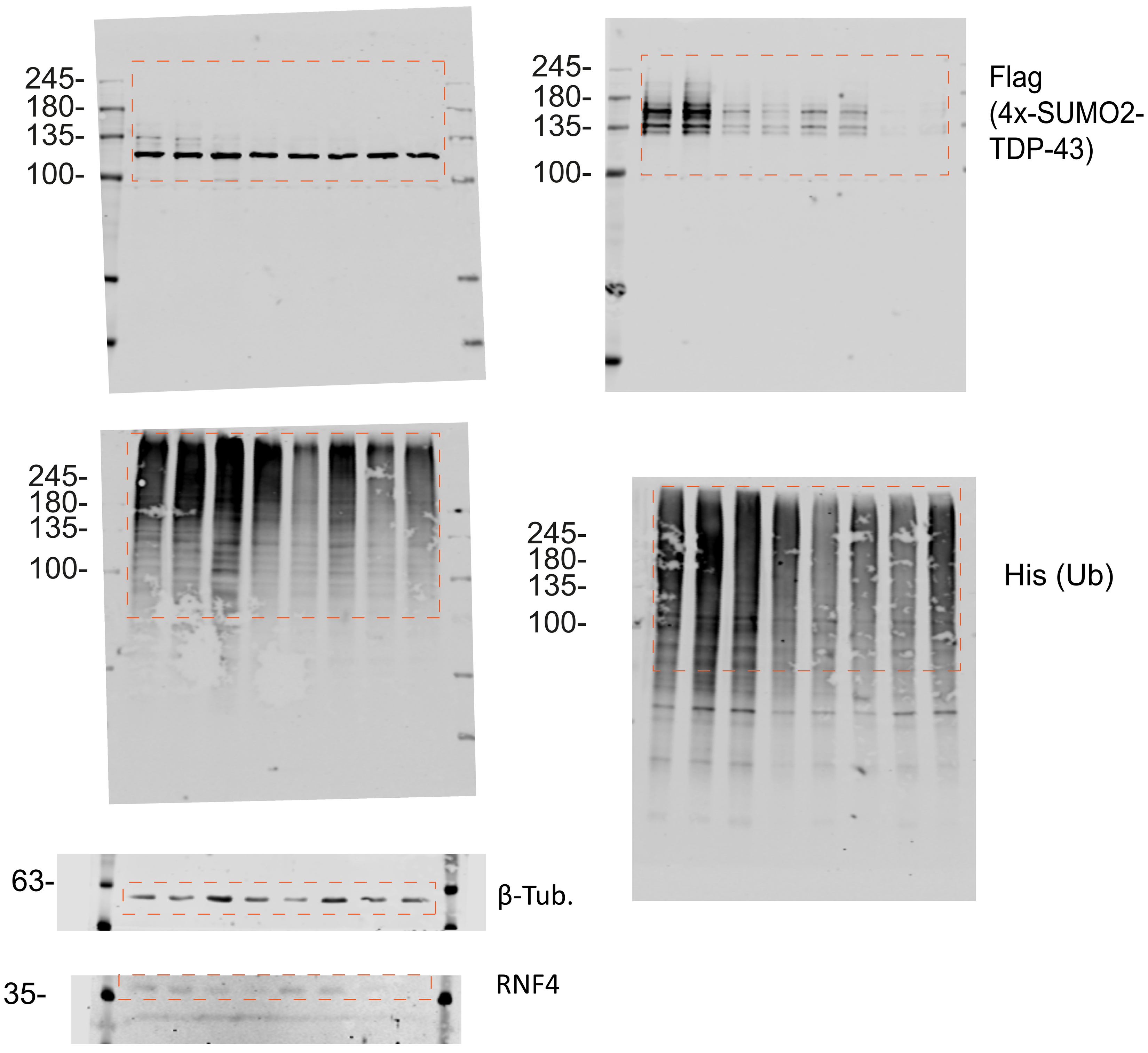
245-
180-
135-
100-
Flag
(4x-SUMO2-
TDP-43)
His (Ub)
63-
β-Tub.
35-
RNF4

3b

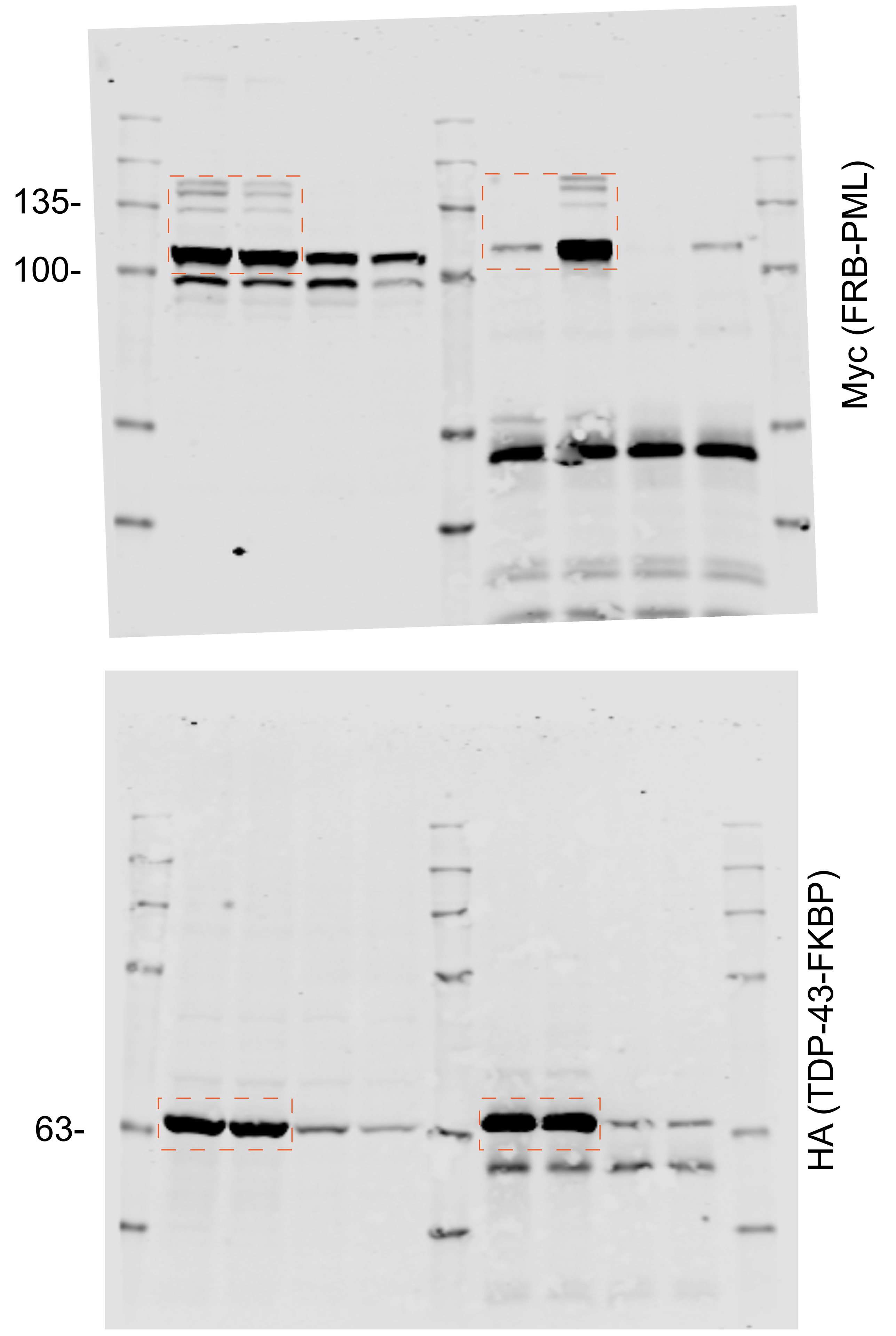
135-
100-
Myc (FRB-PML)
63-
HA (TDP-43-FKBP)

3d

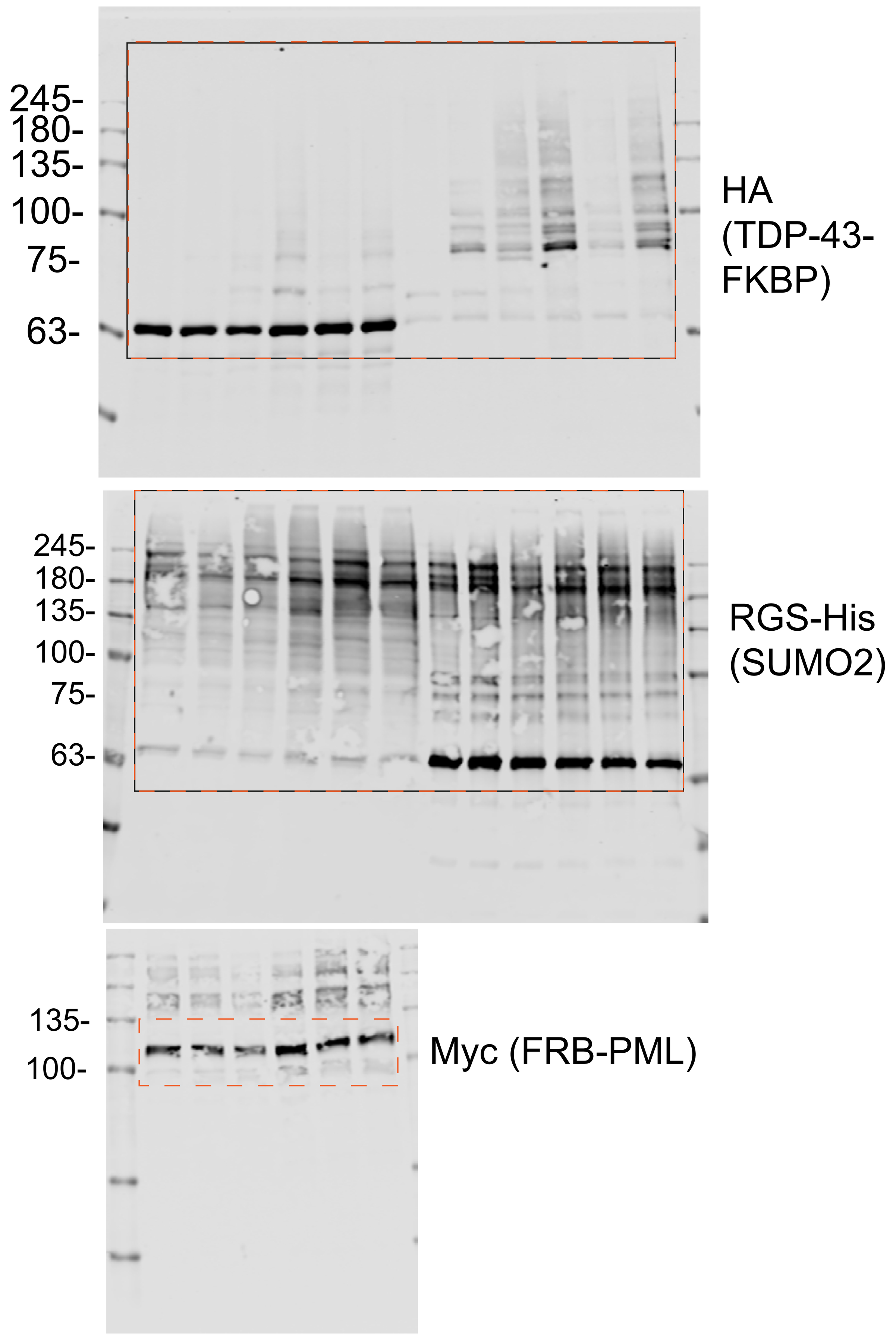
245-
180-
135-
100-
75-
63-
HA
(TDP-43-
FKBP)
245-
180-
135-
100-
75-
63-
RGS-His
(SUMO2)
135-
100-
Myc (FRB-PML)

3e

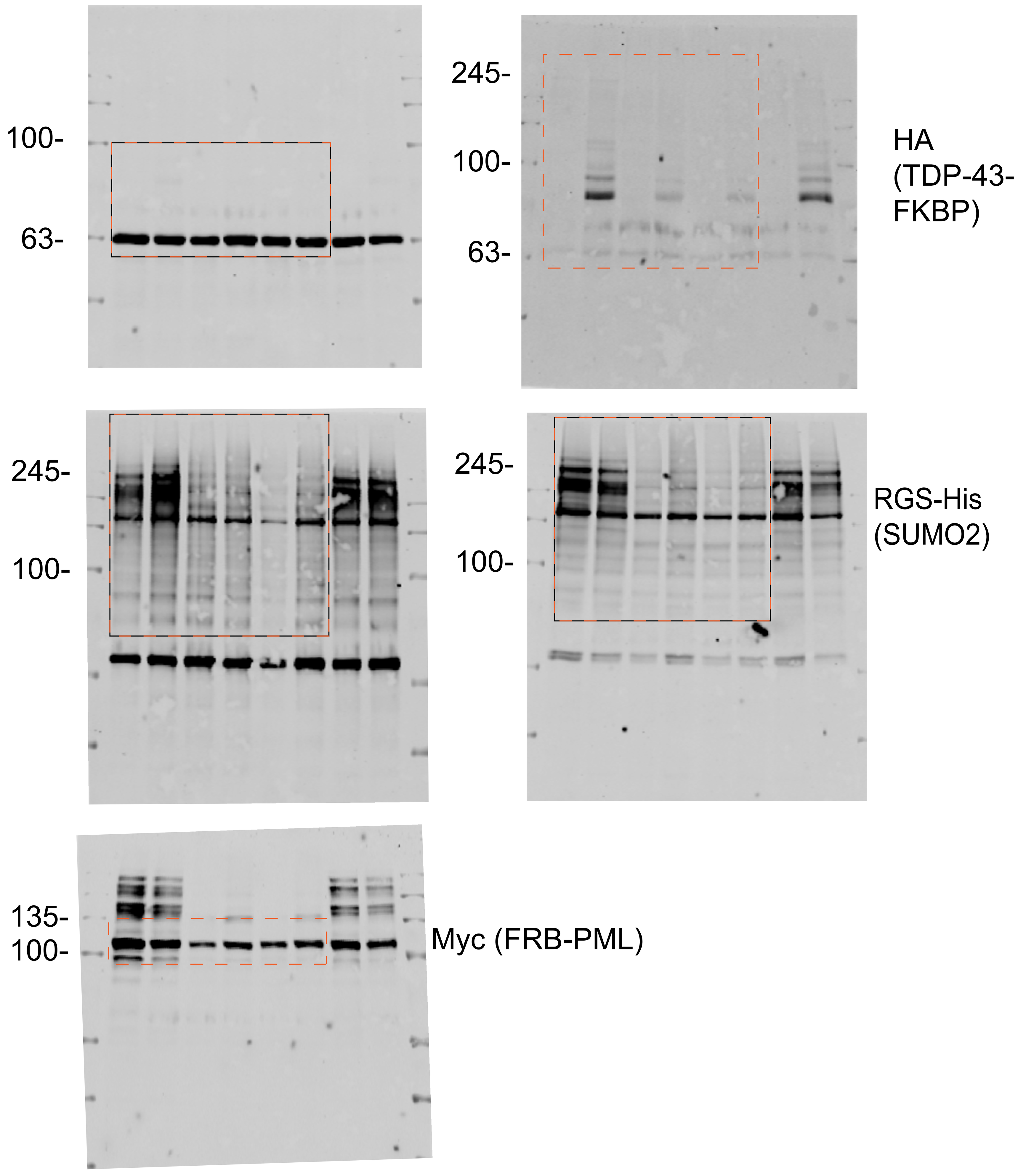
100-
63-
245-
100-
63-
HA
(TDP-43-
FKBP)
245-
100-
245-
100-
RGS-His
(SUMO2)
135-
100-
Myc (FRB-PML)

3f

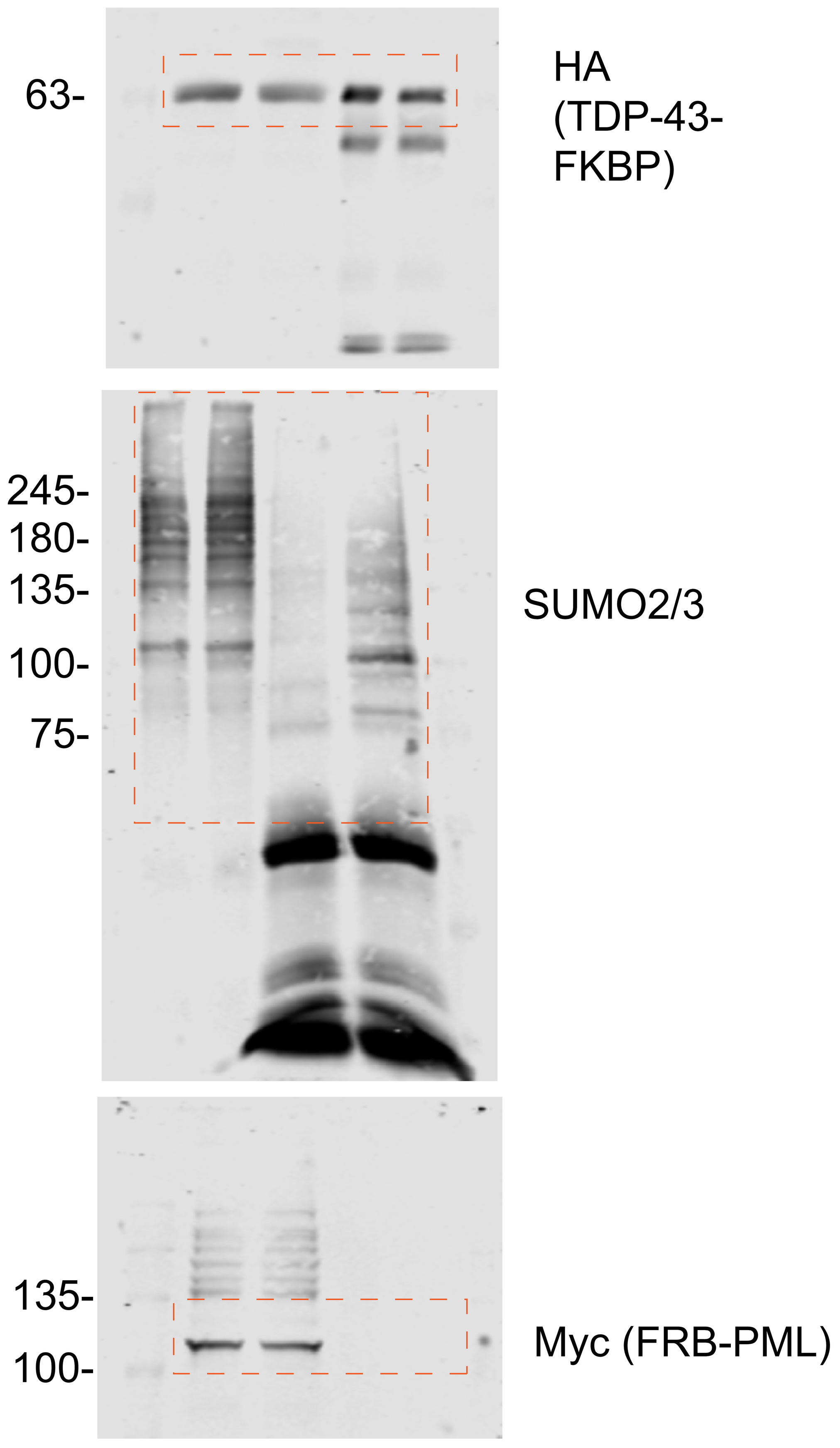
63-
HA
(TDP-43-
FKBP)
245-
180-
135-
100-
75-
SUMO2/3
135-
100-
Myc (FRB-PML)

3g

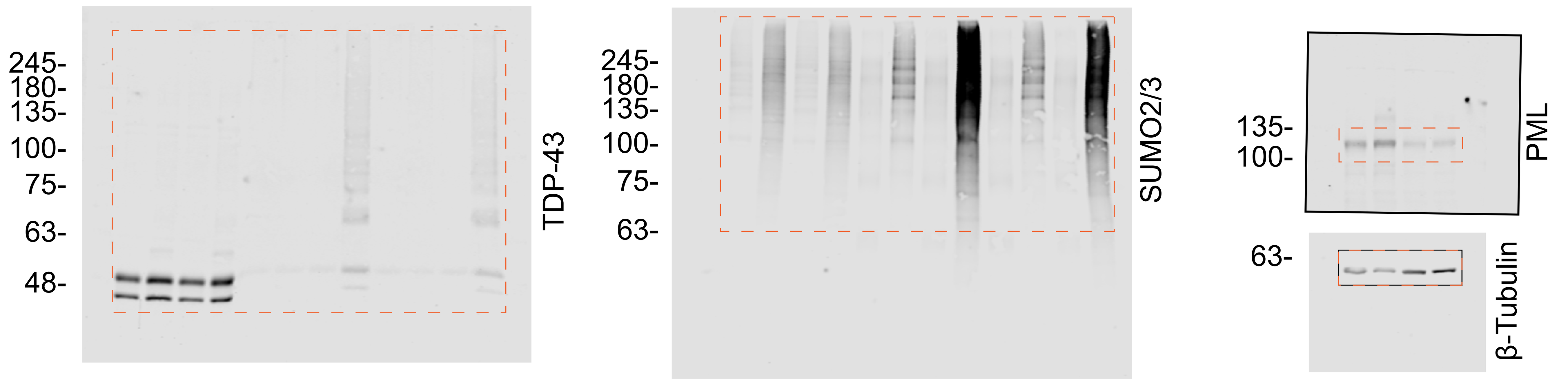
245-
180-
135-
100-
75-
63-
48-
TDP-43
245-
180-
135-
100-
75-
63-
SUMO2/3
135-
100-
PML
63-
β-Tubulin

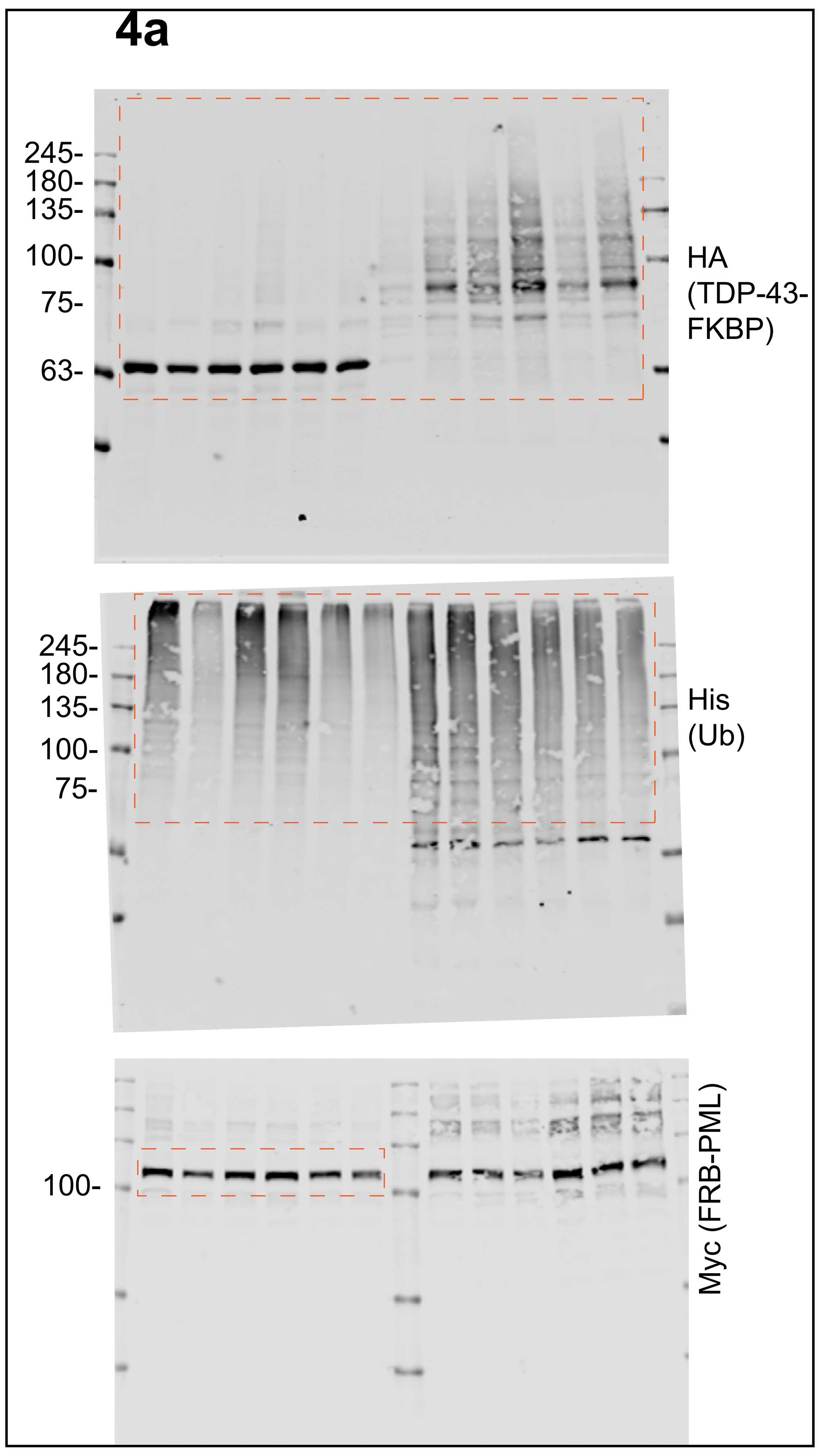
4a
245-
180-
135-
100-
75-
63-
HA (TDP-43-FKBP)
245-
180-
135-
100-
75-
His (Ub)
100-
Myc (FRB-PML)

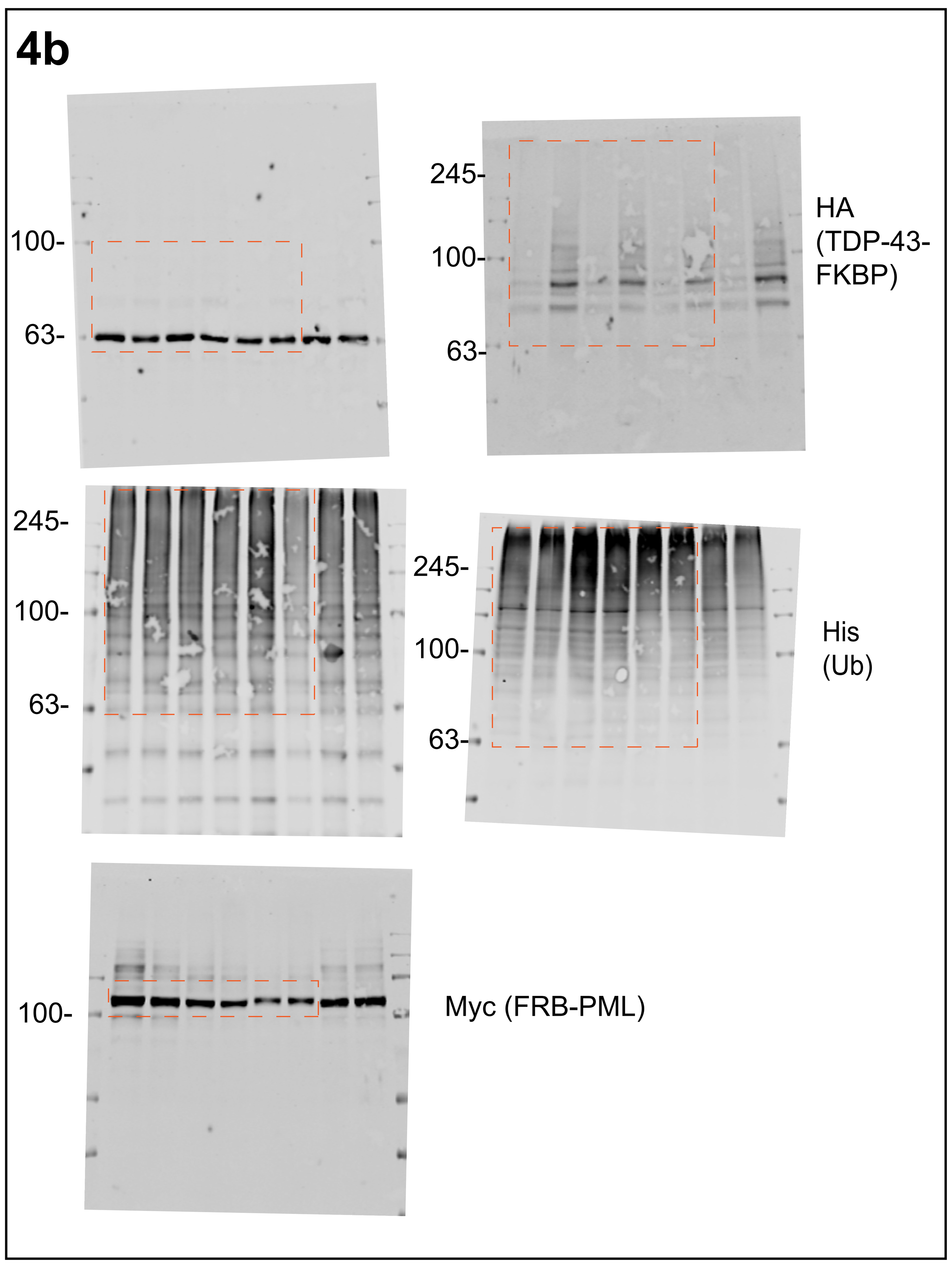
4b
100-
63-
245-
100-
63-
HA (TDP-43-FKBP)
245-
100-
63-
245-
100-
63-
His (Ub)
100-
Myc (FRB-PML)

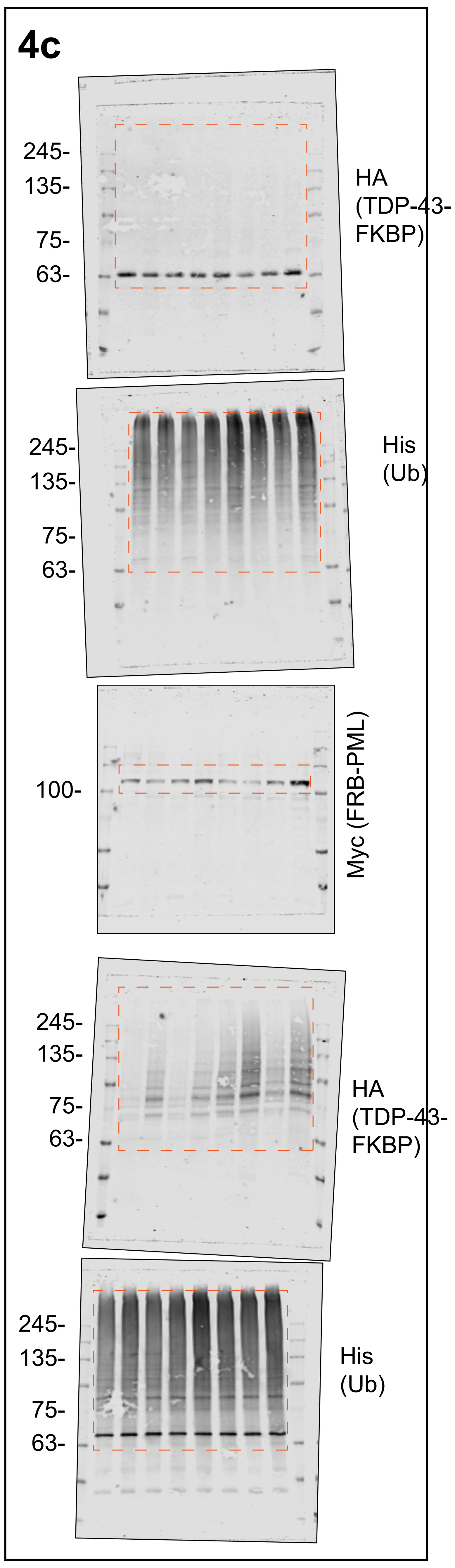
4c
245-
135-
75-
63-
HA (TDP-43-FKBP)
245-
135-
75-
63-
His (Ub)
100-
Myc (FRB-PML)
245-
135-
75-
63-
HA (TDP-43-FKBP)
245-
135-
75-
63-
His (Ub)

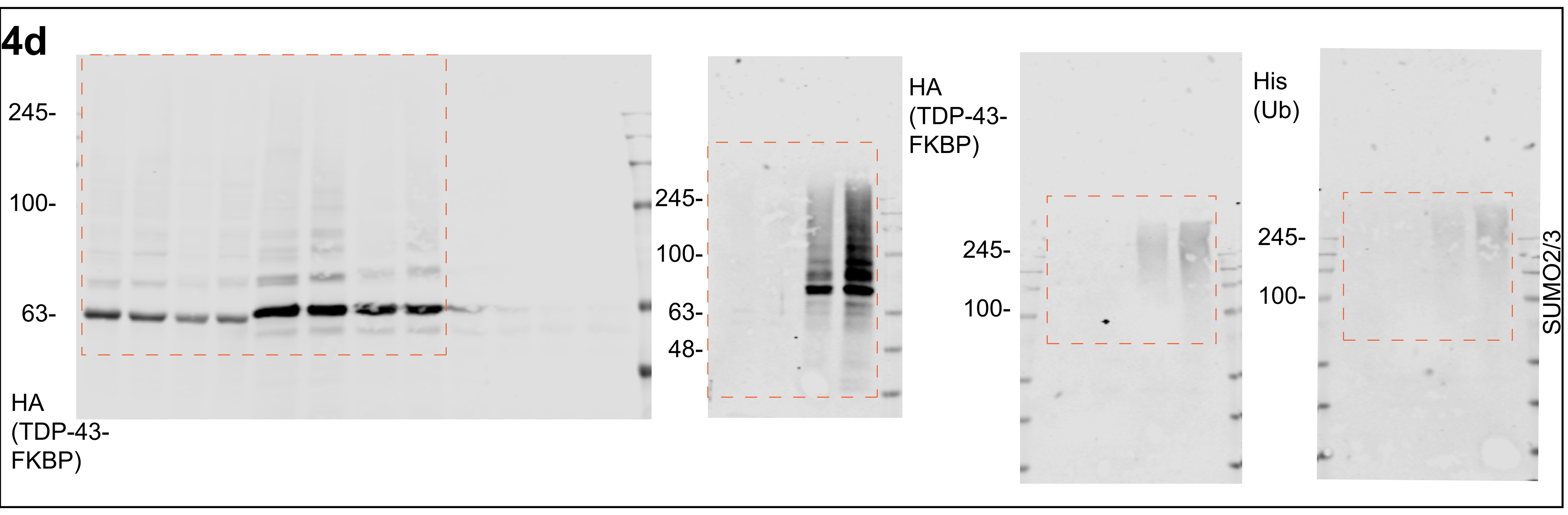
4d
245-
100-
63-
HA (TDP-43-FKBP)
245-
100-
63-
48-
HA (TDP-43-FKBP)
245-
100-
His (Ub)
245-
100-
SUMO2/3

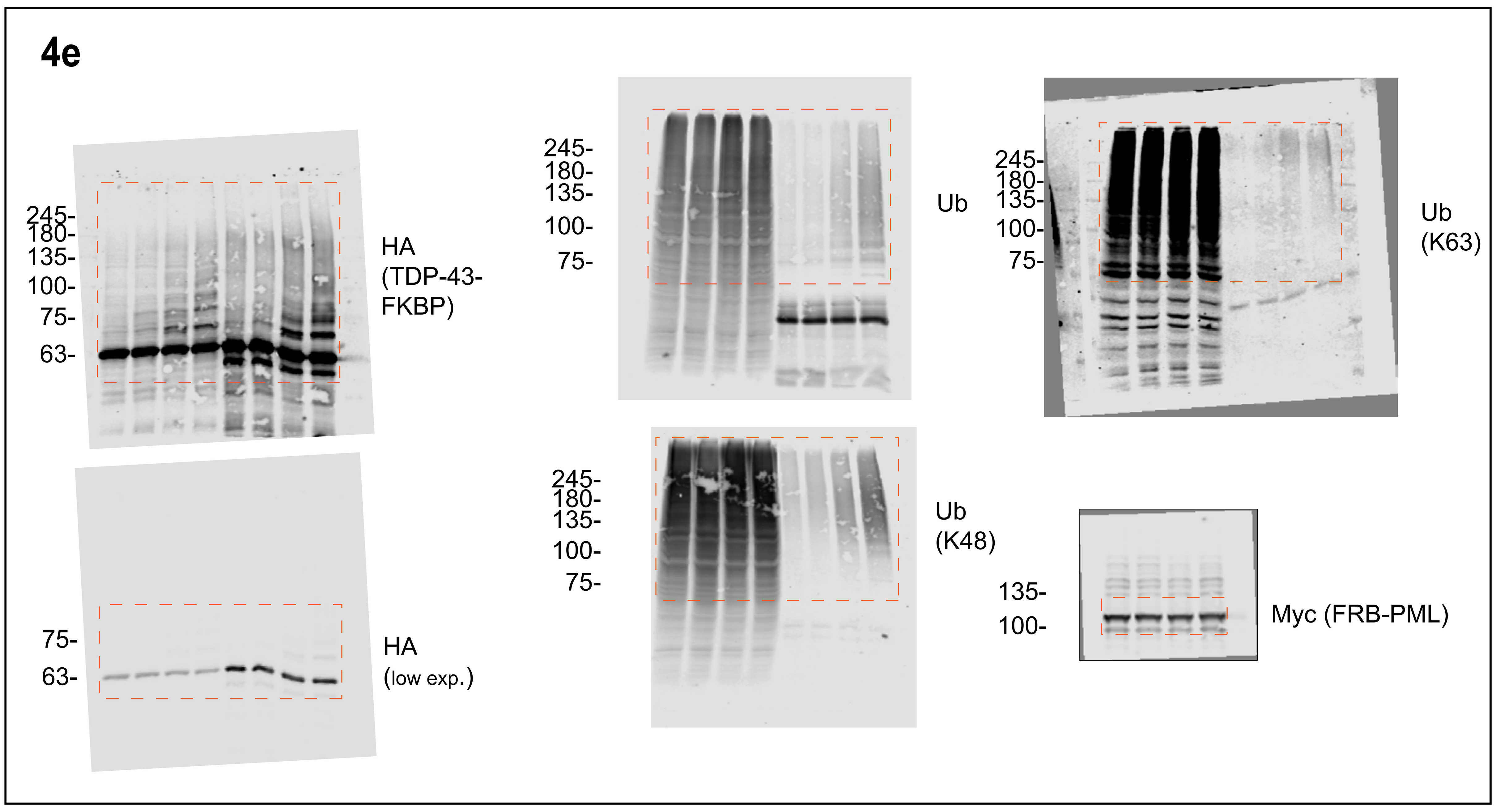
4e
245-
180-
135-
100-
75-
63-
HA (TDP-43-FKBP)
75-
63-
HA (low exp.)
245-
180-
135-
100-
75-
Ub
245-
180-
135-
100-
75-
Ub (K48)
245-
180-
135-
100-
75-
Ub (K63)
135-
100-
Myc (FRB-PML)

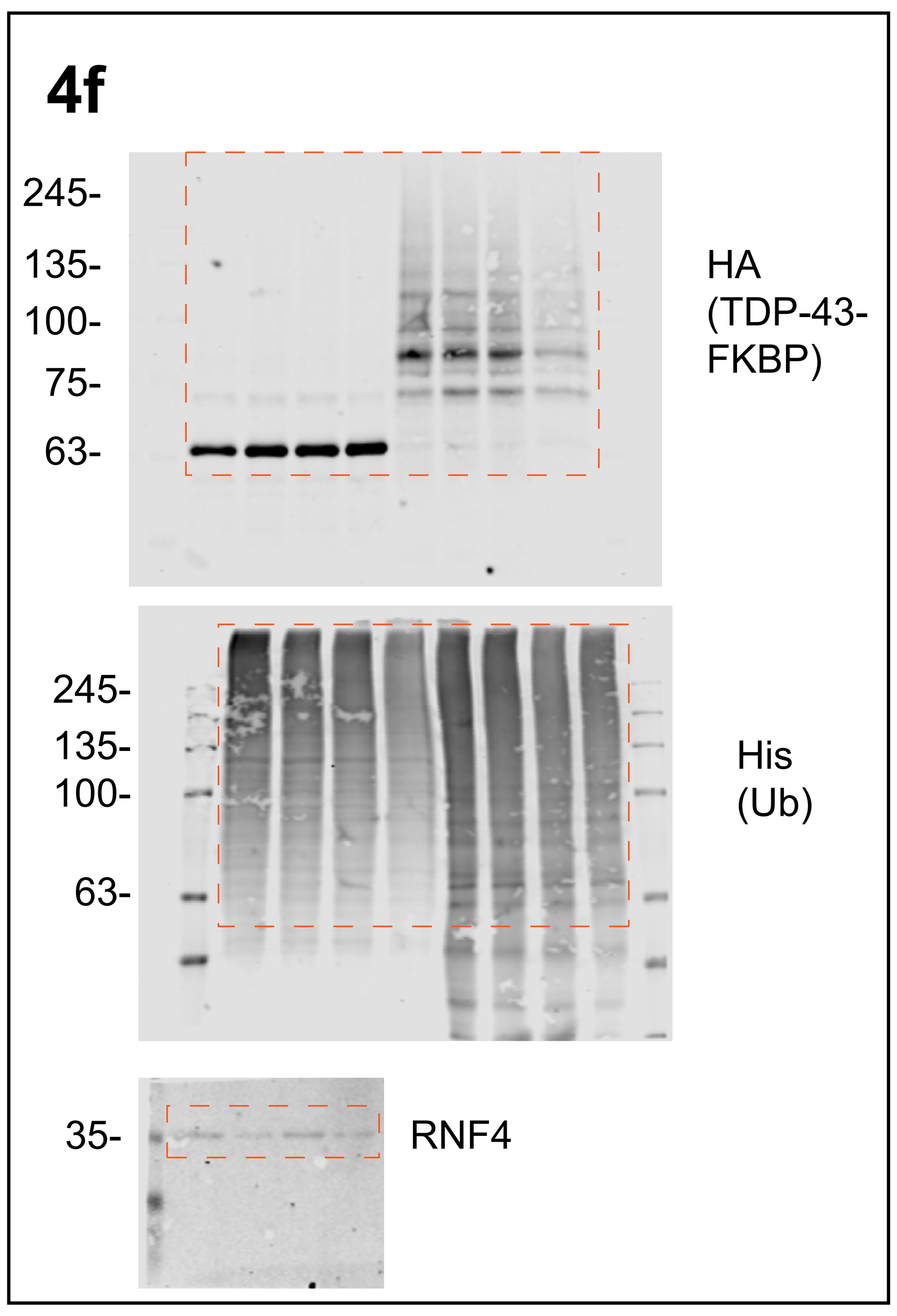
4f
245-
135-
100-
75-
63-
HA (TDP-43-FKBP)
245-
135-
100-
63-
His (Ub)
35-
RNF4

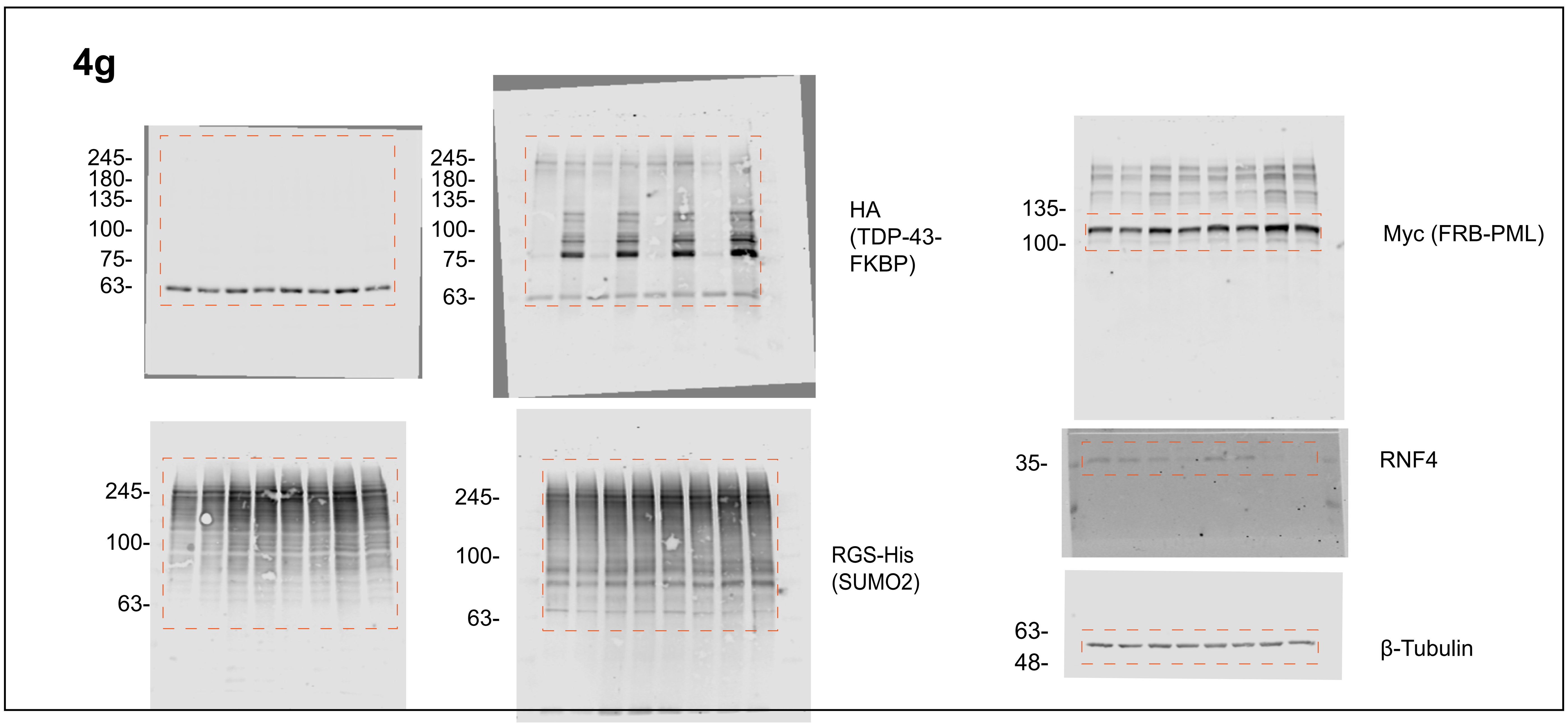
4g
245-
180-
135-
100-
75-
63-
245-
180-
135-
100-
75-
63-
HA (TDP-43-FKBP)
245-
100-
63-
245-
100-
63-
RGS-His (SUMO2)
135-
100-
Myc (FRB-PML)
35-
RNF4
63-
48-
β-Tubulin

5a

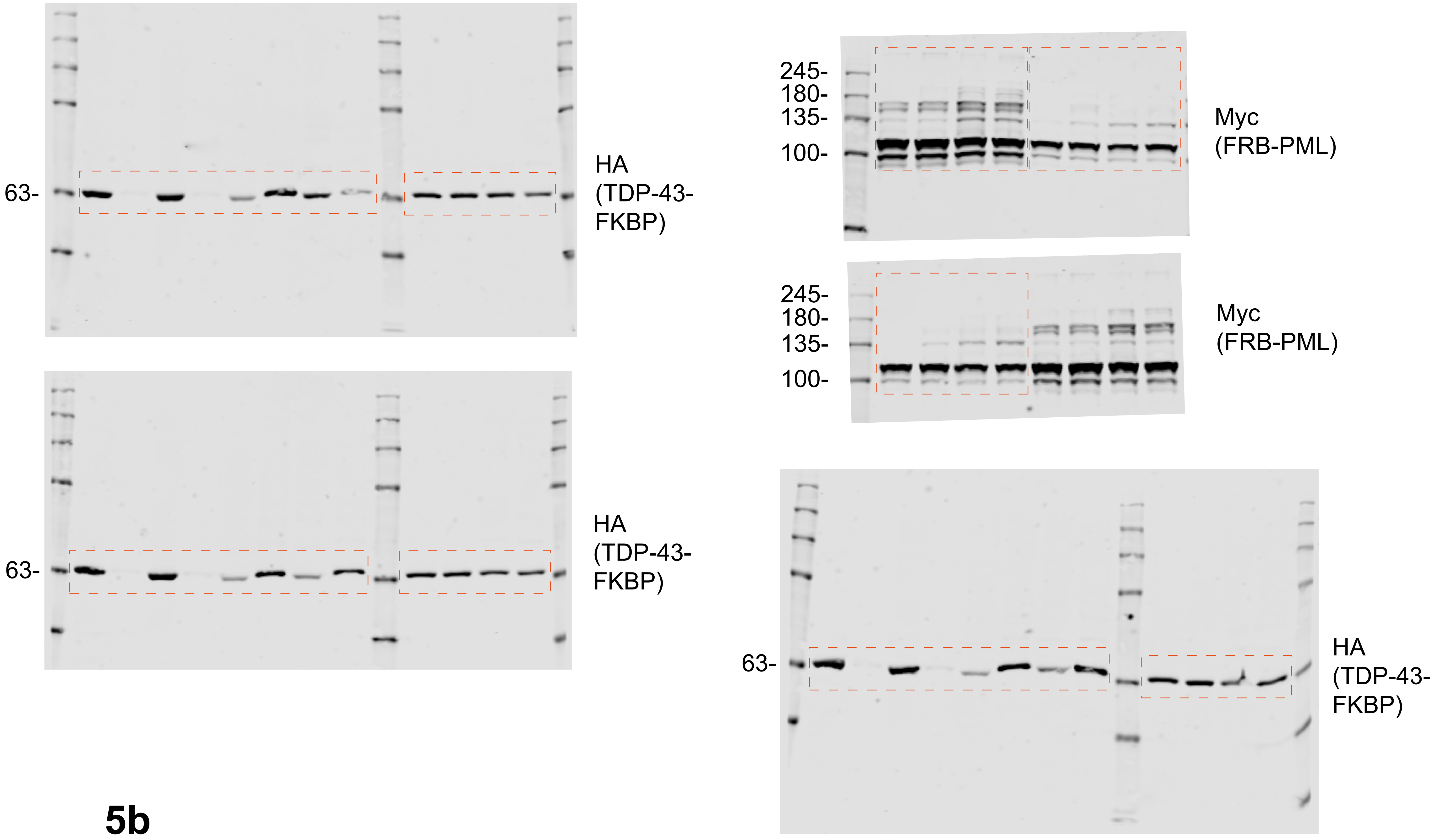
63-
HA
(TDP-43-
FKBP)
63-
HA
(TDP-43-
FKBP)
245-
180-
135-
100-
Myc
(FRB-PML)
245-
180-
135-
100-
Myc
(FRB-PML)
63-
HA
(TDP-43-
FKBP)

5b

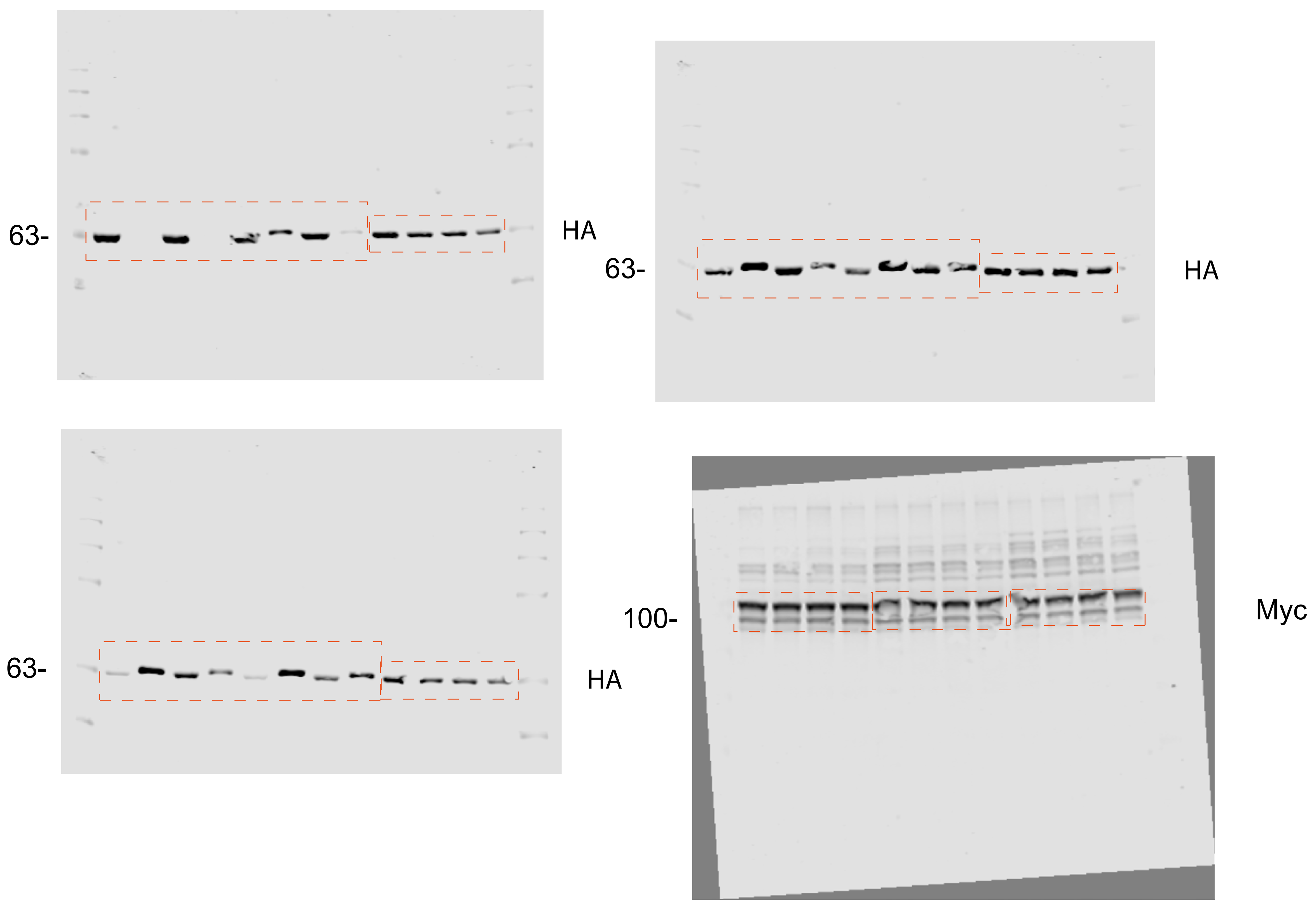
63-
HA
63-
HA
63-
HA
100-
Myc

5c

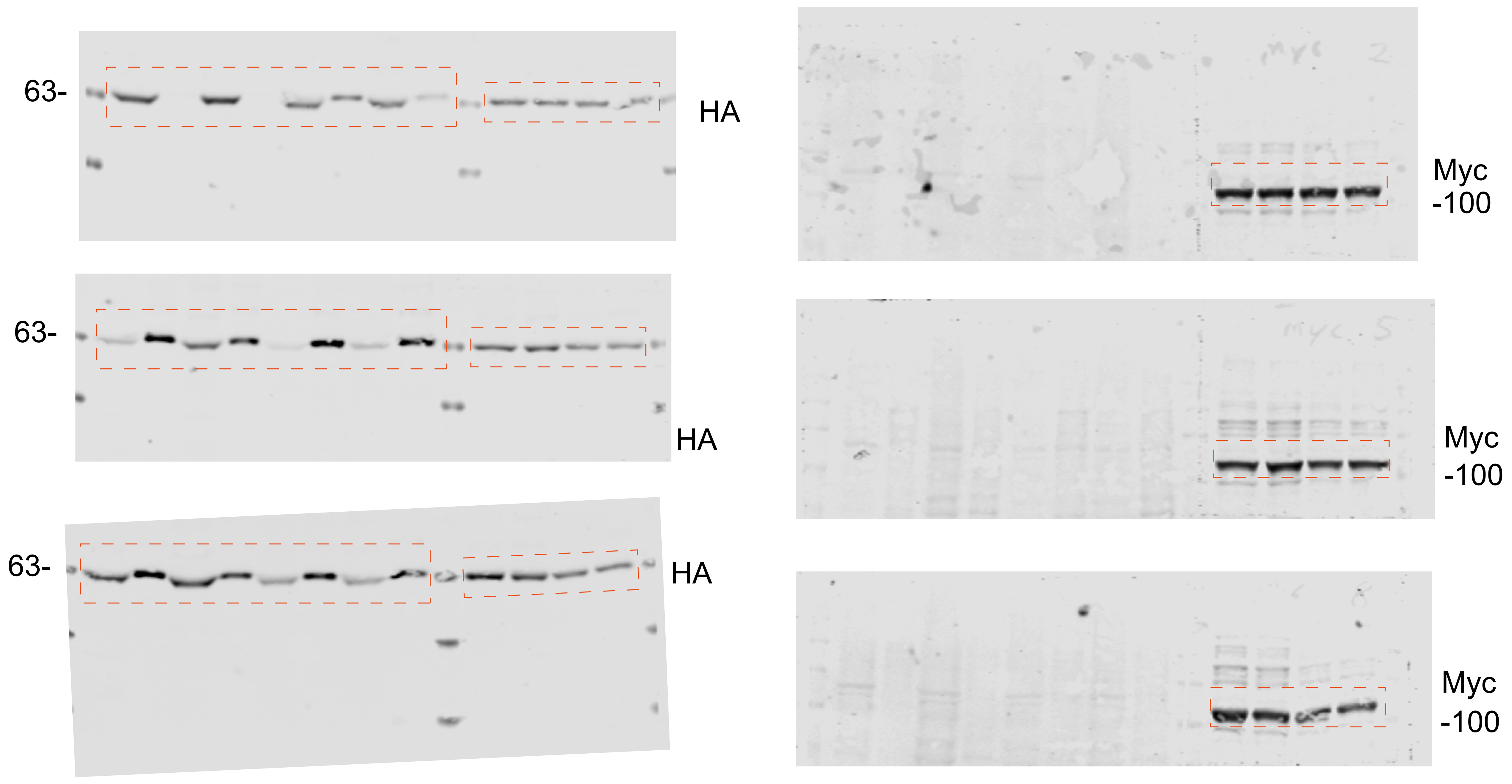
63-
HA
63-
HA
63-
HA
Myc
-100
Myc
-100
Myc
-100

5e

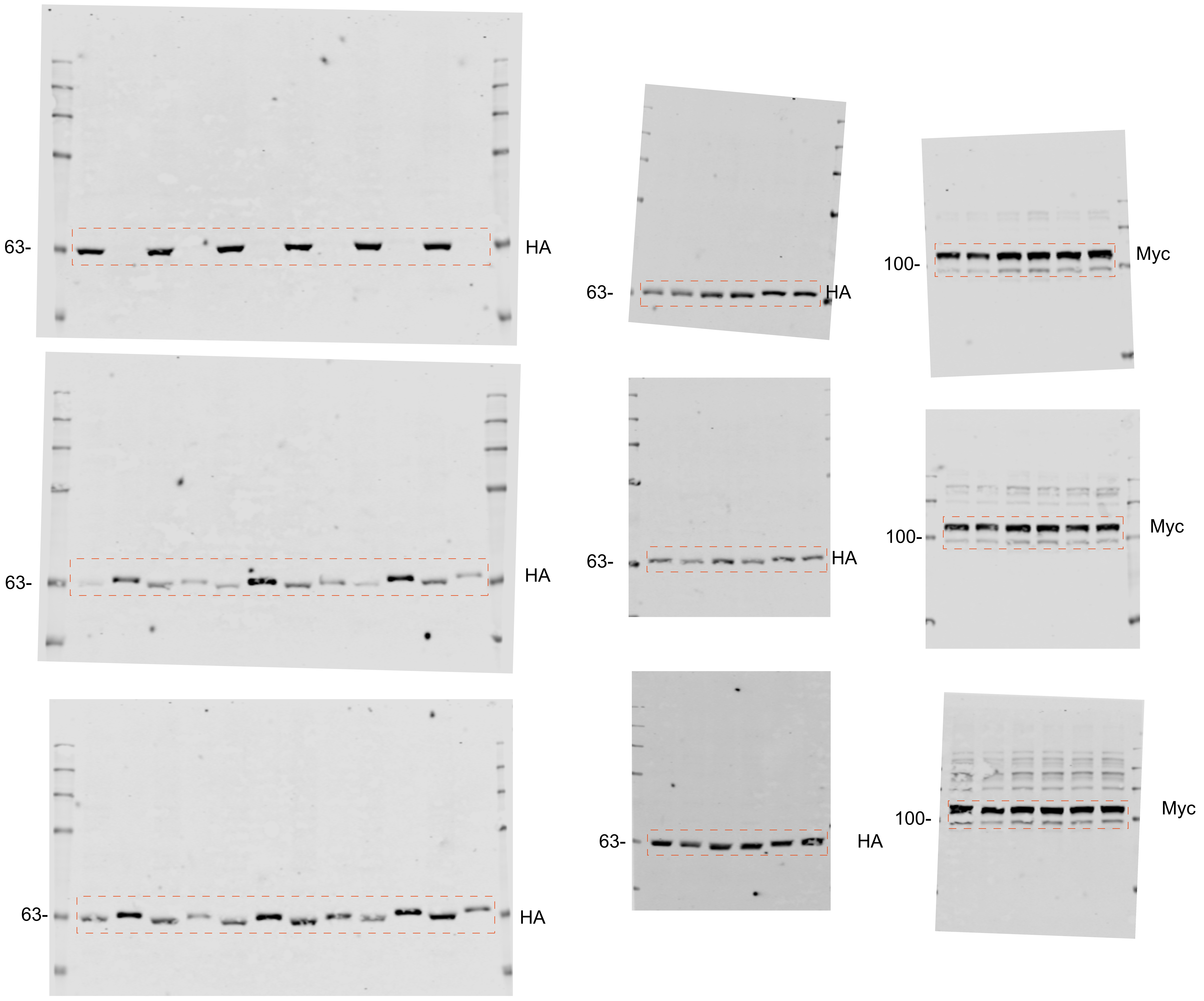
63-
HA
63-
HA
63-
HA
63-
HA
63-
HA
63-
HA
100-
Myc
100-
Myc
100-
Myc

5f

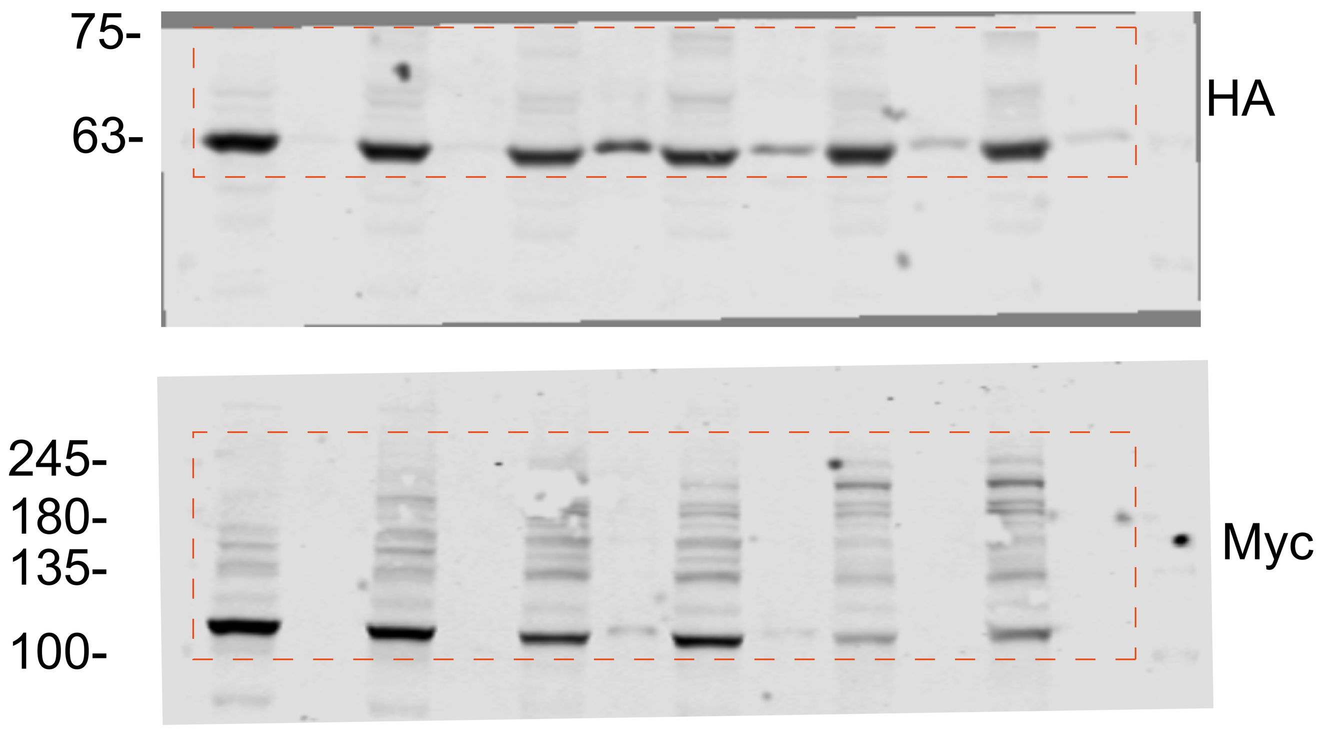
75-
63-
HA
245-
180-
135-
100-
Myc

5i

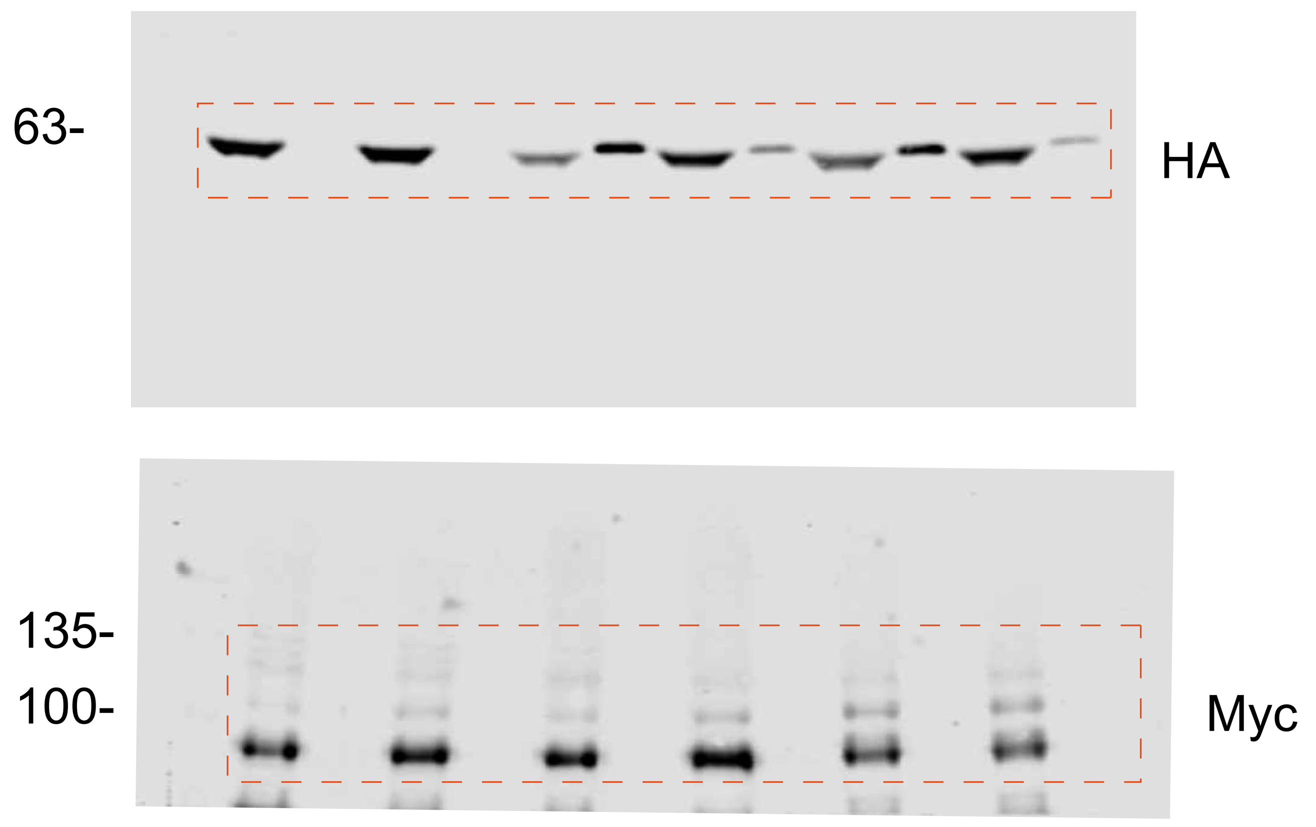
63-
HA
135-
100-
Myc

5g

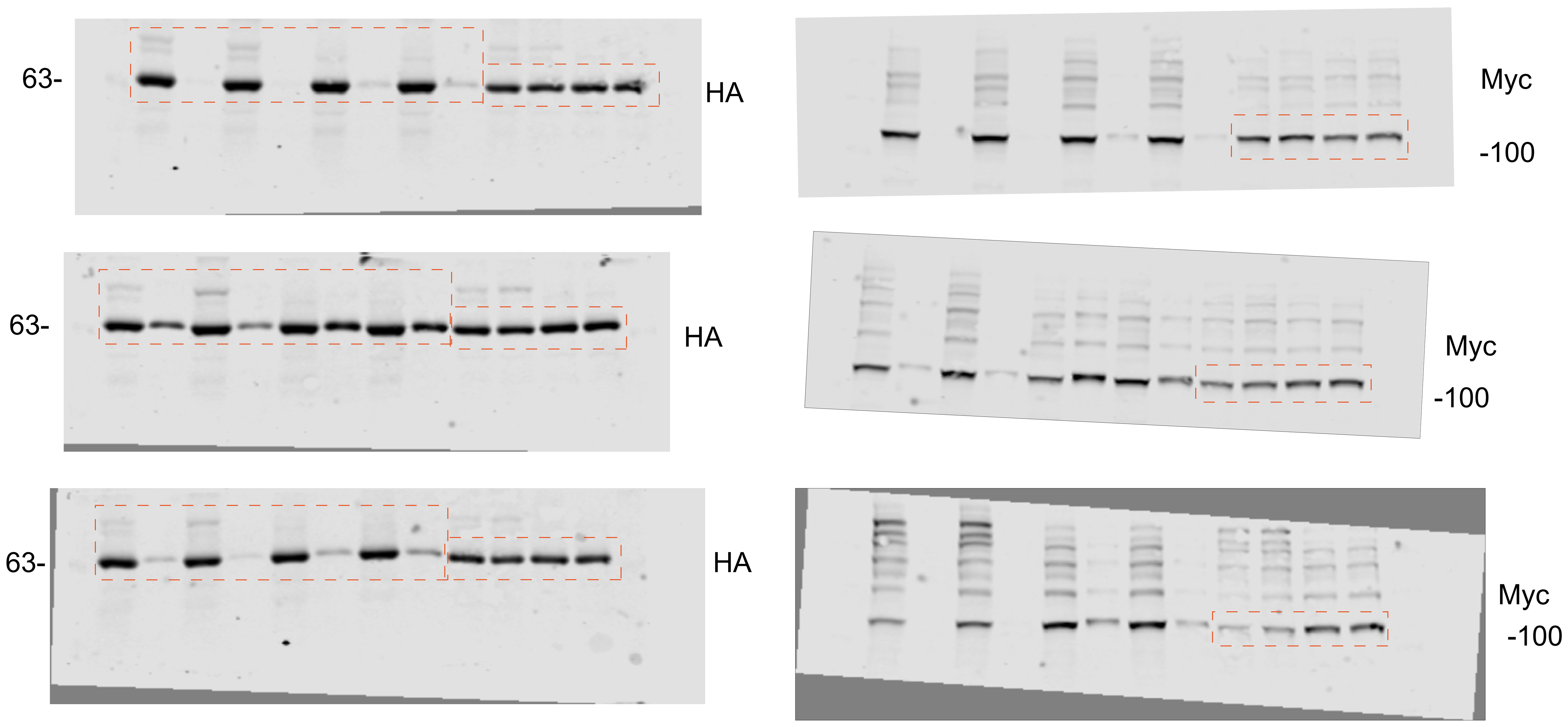
63-
HA
Myc
-100
63-
HA
Myc
-100
63-
HA
Myc
-100

Extended Data Fig. 1b

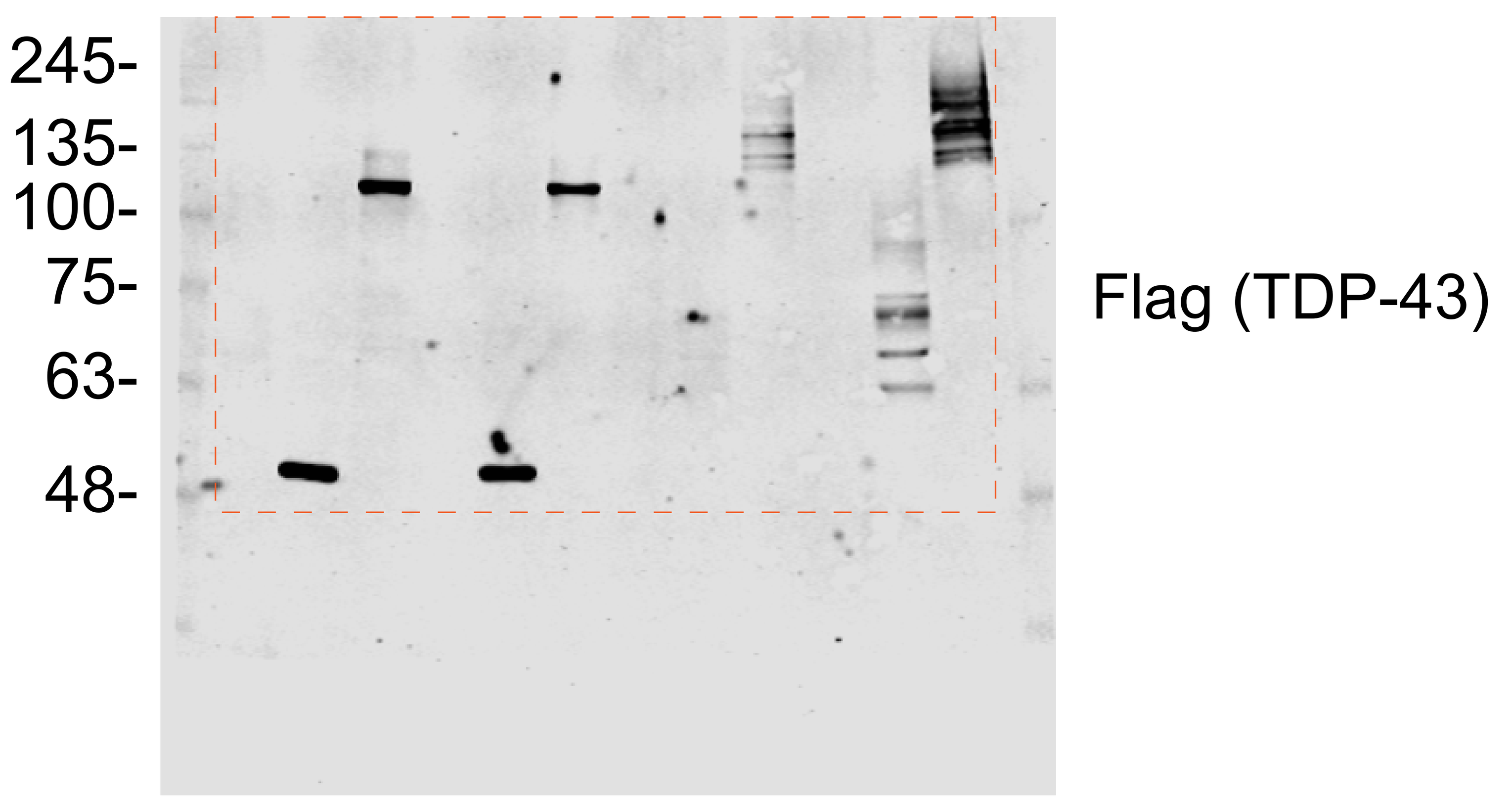

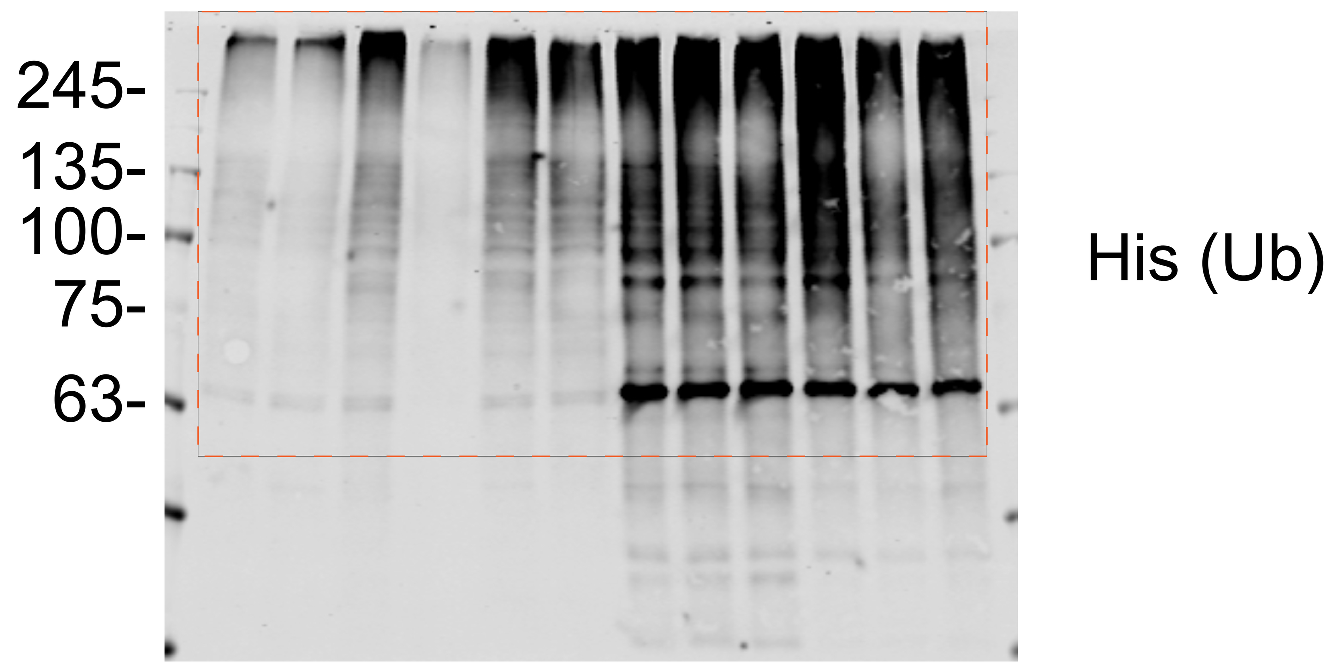

Extended Data Fig. 4a

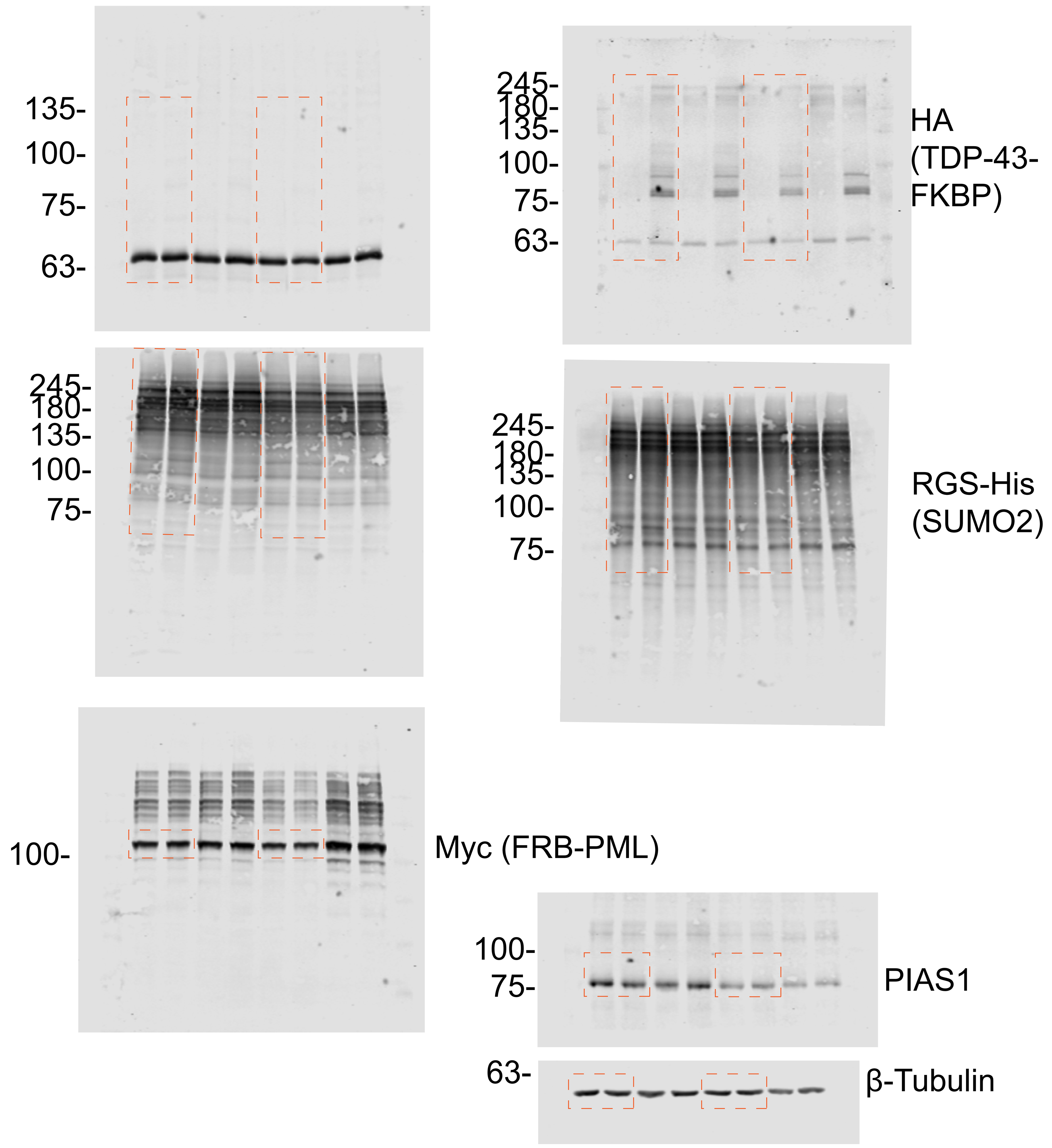

Extended Data Fig. 4b

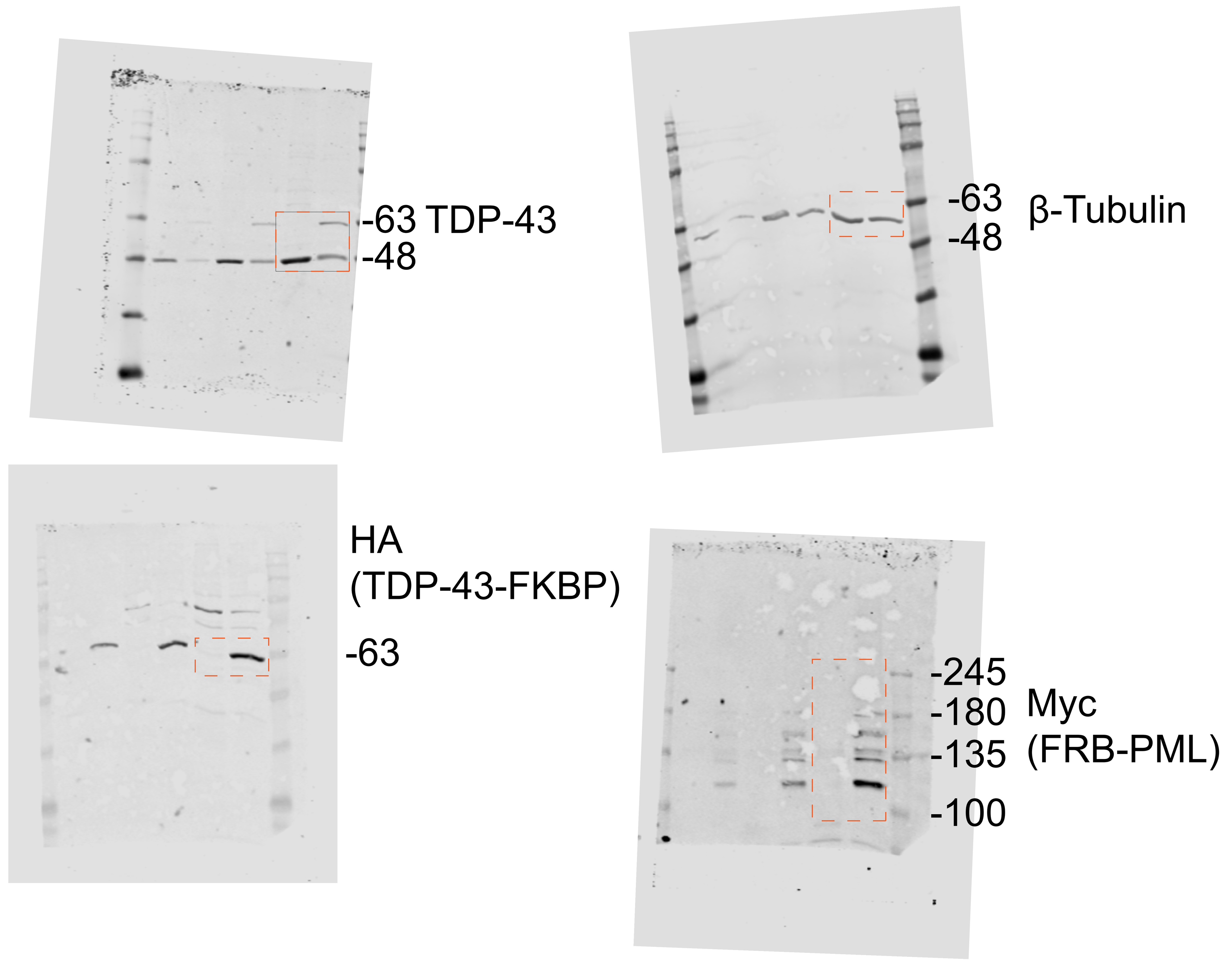

Extended Data Fig. 5a

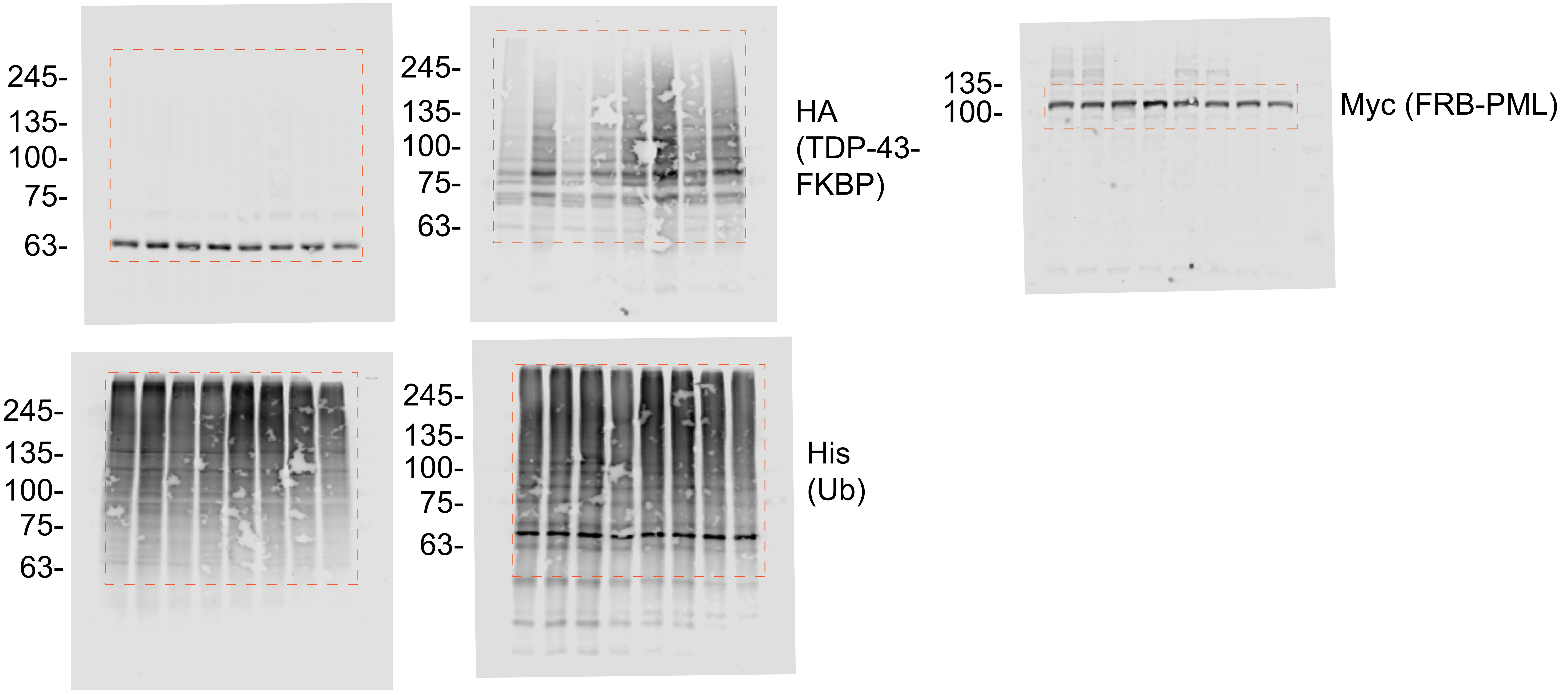

Extended Data Fig. 5b

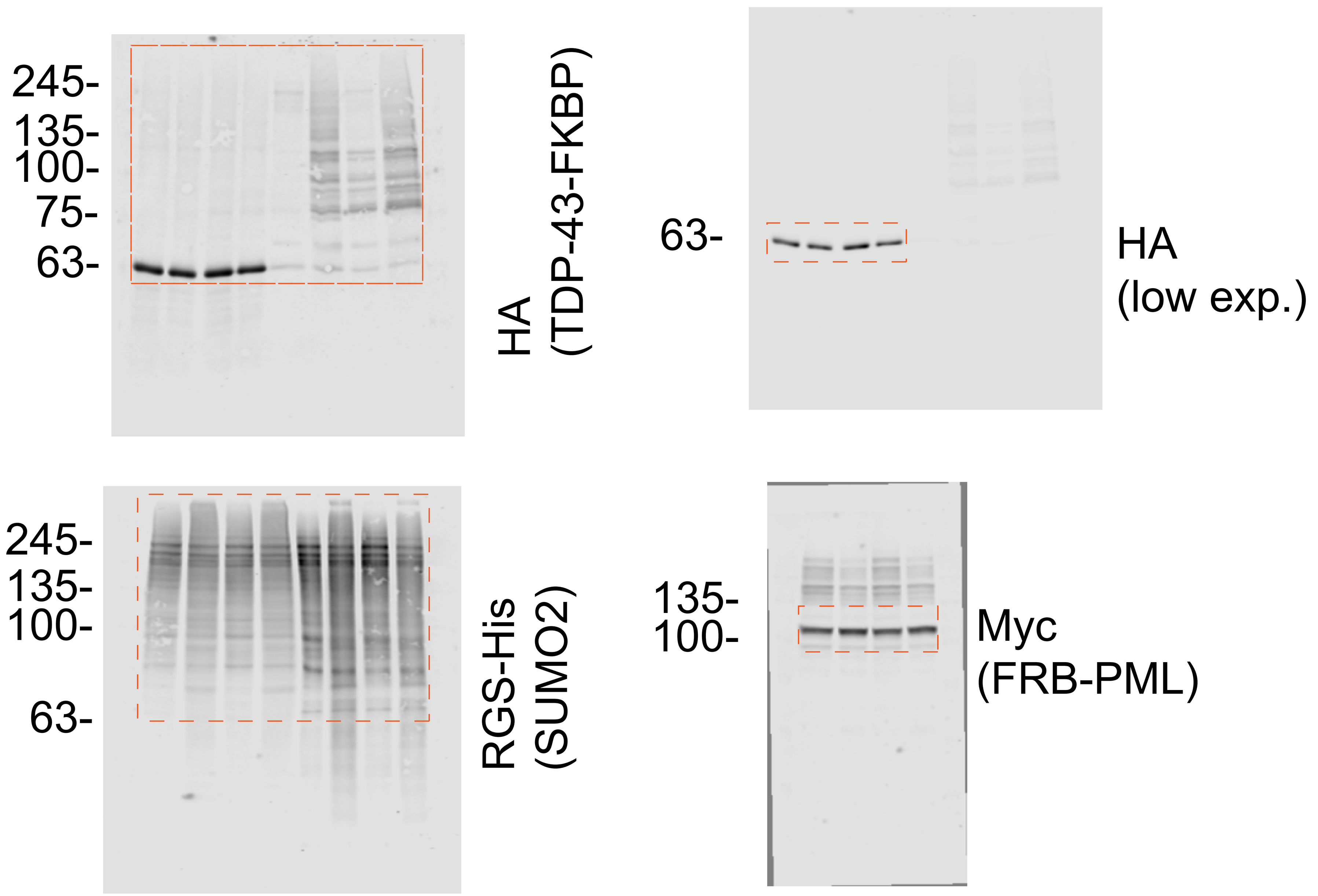

Extended Data Fig. 5c

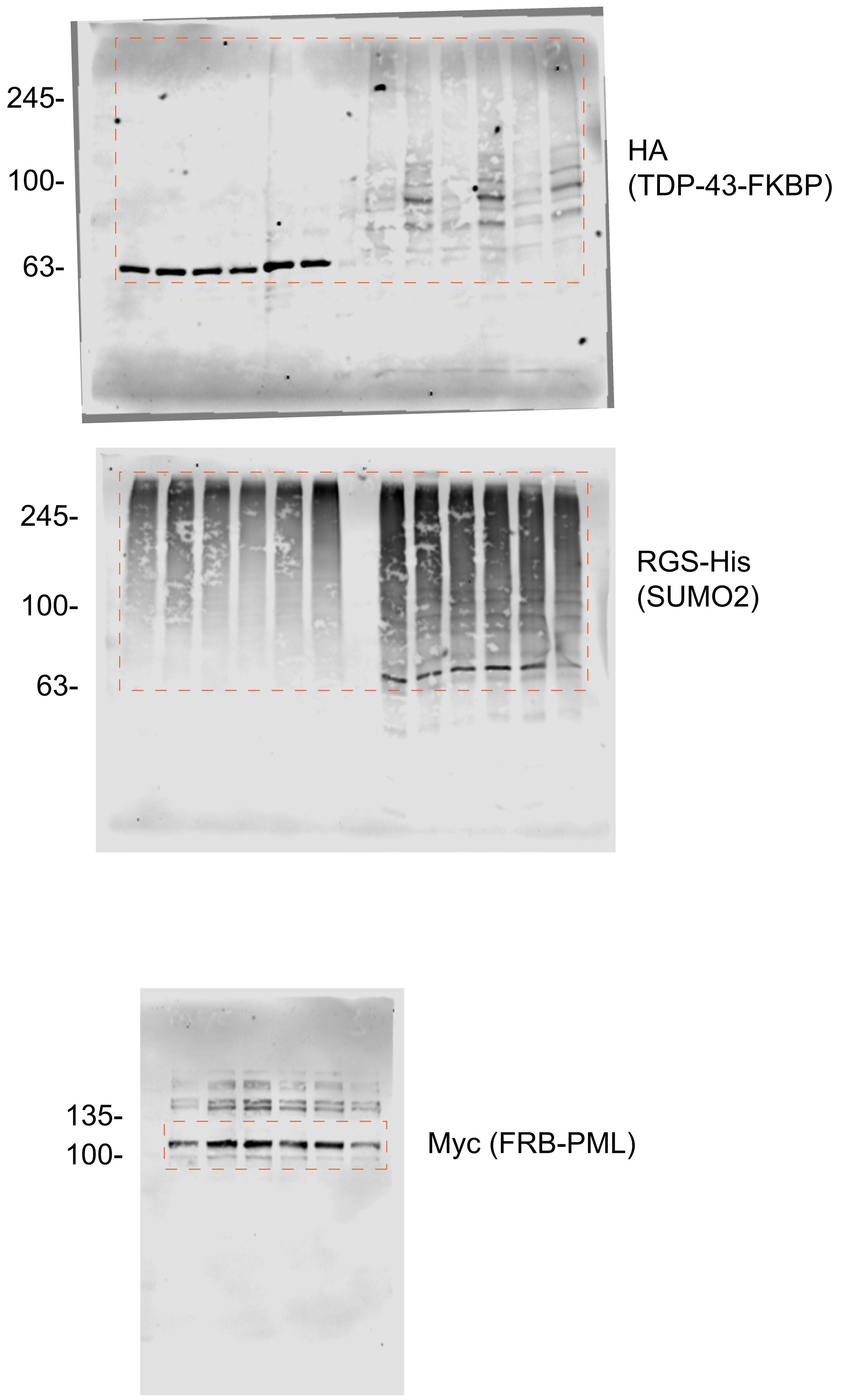

Extended Data Fig. 5d

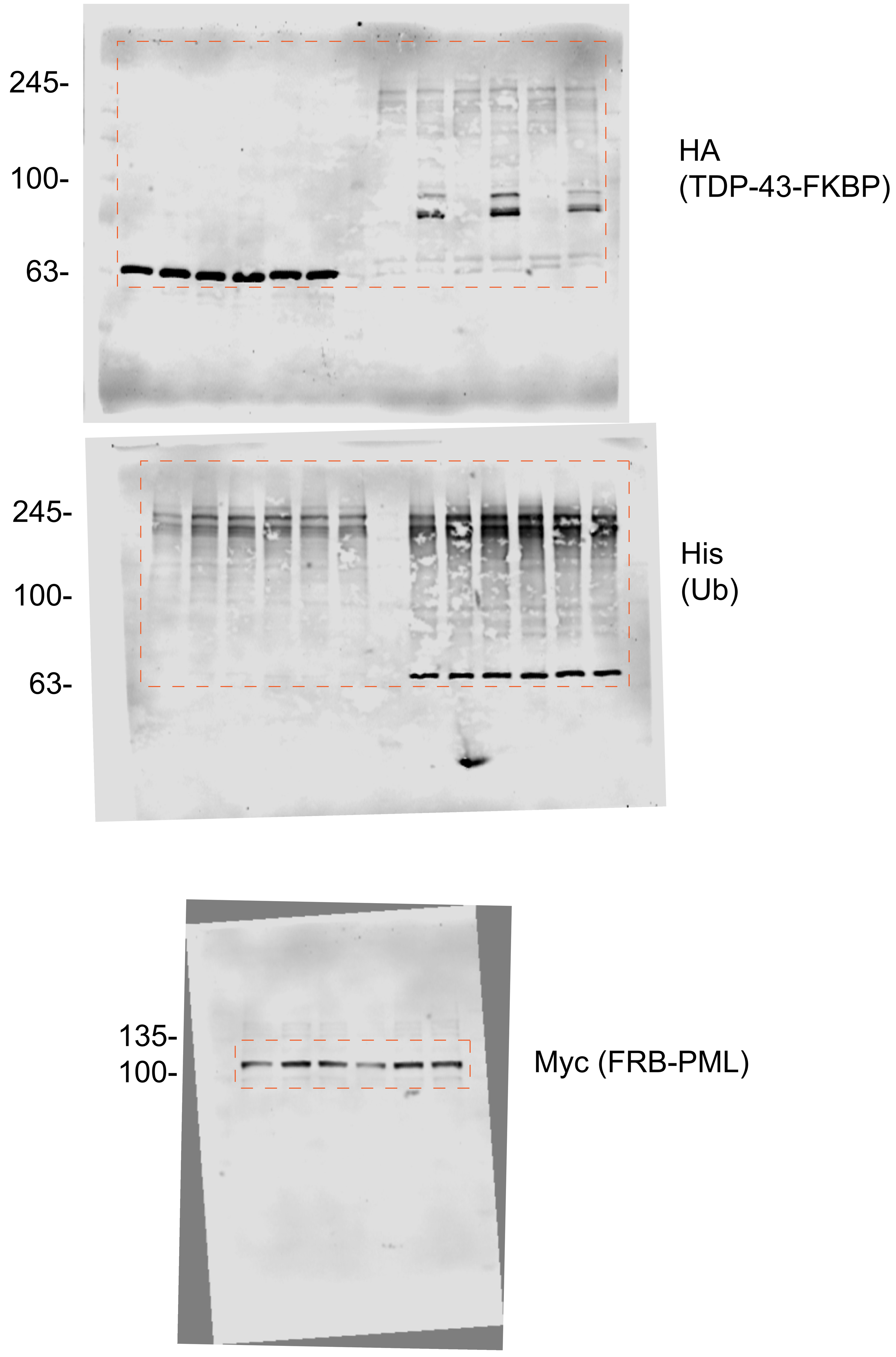

Supplement: Supplementary file 4 — Western blot (uncropped, unedited blots) images for Figs. 1b–e, 2d–g, 3b,d–g, 4a–g and 5a–c,e–g,i and Extended Data Figs. 1b, 4a,b and 5a–d. [file 41589_2025_1886_MOESM4_ESM.pdf]
